# Protective effect of oxytocin on vincristine-induced gastrointestinal dysmotility in mice

**DOI:** 10.3389/fphar.2024.1270612

**Published:** 2024-04-09

**Authors:** Shuang Li, Yao Shi, Jianchun Zhu, Jingxin Li, Shuanglian Wang, Chuanyong Liu

**Affiliations:** ^1^ Department of Physiology, School of Basic Medical Sciences, Cheeloo Medical College, Shandong University, Jinan, China; ^2^ Ministry of Education Key Laboratory of Protein, School of Life Sciences, Tsinghua University, Beijing, China; ^3^ Medical Science and Technology Innovation Center, Shandong First Medical University and Shandong Academy of Medical Sciences, Jinan, China; ^4^ Provincial Key Lab of Mental Disorders, Shandong University, Jinan, China

**Keywords:** vincristine, oxytocin, gastrointestinal motility, myenteric neurons, oxidative stress, MAPK pathways

## Abstract

**Aims:** Vincristine (VCR), an antineoplastic drug, induces peripheral neuropathy characterized by nerve damage, limiting its use and reducing the quality of life of patients. VCR causes myenteric neuron damage, inhibits gastrointestinal motility, and results in constipation or paralytic ileus in patients. Oxytocin (OT) is an endogenous neuropeptide produced by the enteric nerve system, which regulates gastrointestinal motility and exerts neuroprotective effects. This study aimed to investigate whether OT can improve VCR-induced gastrointestinal dysmotility and evaluate the underlying mechanism.

**Methods:** Mice were injected either with saline or VCR (0.1 mg/kg/d, i. p.) for 14 days, and OT (0.1 mg/kg/d, i.p.) was applied 1 h before each VCR injection. Gastrointestinal transit and the contractile activity of the isolated colonic segments were assessed. The concentration of OT in plasma was measured using ELISA. Immunofluorescence staining was performed to analyze myenteric neurons and reactive oxygen species (ROS) levels. Furthermore, the indicators of oxidative stress were detected. The protein expressions of Nrf2, ERK1/2, P-ERK1/2, p38, and P-p38 in the colon were tested using Western blot.

**Results:** VCR reduced gastrointestinal transit and the responses of isolated colonic segments to electrical field stimulation and decreased the amount of neurons. Furthermore, VCR reduced neuronal nitric oxide synthase and choline acetyltransferase immunopositive neurons in the colonic myenteric nerve plexus. VCR increased the concentration of OT in plasma. Exogenous OT pretreatment ameliorated the inhibition of gastrointestinal motility and the injury of myenteric neurons caused by VCR. OT pretreatment also prevented the decrease of superoxide dismutase activity, glutathione content, total antioxidative capacity, and Nrf2 expression, the increase of ROS levels, and the phosphorylation of ERK1/2 and p38 MAPK following VCR treatment.

**Conclusion:** Our results suggest that OT pretreatment can protect enteric neurons from VCR-induced injury by inhibiting oxidative stress and MAPK pathways (ERK1/2, p38). This may be the underlying mechanism by which it alleviates gastrointestinal dysmotility.

## 1 Introduction

One of the primary characteristics of vincristine (VCR) is its antineoplastic properties, which are beneficial for patients. VCR has been used to treat various cancers. The second property includes its undesirable side effects, which cause pain, limiting the treatment and substantially reducing the quality of life of patients (van de Velde et al., 2021). VCR binds to β-tubulin, inhibits microtubule polymerization at low concentrations or promotes microtubule depolymerization at high concentrations, prevents the formation of mitotic spindles, arrests mitotic progression, and prevents cell division. This is the mechanism underlying the anti-tumor properties of VCR (Triarico et al., 2021). Peripheral neuropathy is the most common dose-restricting side effect of VCR, affecting not only the peripheral sensorimotor nerves but also the autonomic nervous system, increasing the likelihood of damage to visceral organs (Legha, 1986; Dorchin et al., 2013; Triarico et al., 2021). Gastrointestinal toxicity is a common complication of VCR treatment (Moudi et al., 2013; Escalante et al., 2017). Most patients receiving VCR chemotherapy suffer from gastrointestinal diseases, which usually manifest as constipation or even a paralytic ileus, which is life-threatening in severe cases (Tomomasa et al., 1999; Yasu et al., 2016; Escalante et al., 2017; Adil et al., 2021). Experimental studies have reported that VCR delays gastric emptying and inhibits gastrointestinal motility (Sninsky, 1987; Sharma, 1988; Peixoto Júnior et al., 2009; Lopez-Gomez et al., 2018), which may be related to the damage of the gastrointestinal wall and myenteric plexus by VCR ((Sninsky, 1987; Lopez-Gomez et al., 2018; Smith, 1967)). The myenteric nerve plexus within the enteric nervous system (ENS) mainly controls gastrointestinal motility, and its injury usually leads to motility disorders (Spencer and Hu, 2020). Previous results from our lab have shown that VCR can damage myenteric neurons in mice (Gao et al., 2021). This may explain why VCR impairs gastrointestinal motor function.

The classical concept suggests that oxytocin (OT) acts as a neuro-pituitary hormone that is generated in the supraoptic and paraventricular nuclei of the hypothalamus and promotes milk ejection during lactation and uterine contraction during childbirth (Soloff et al., 1979; Jurek and Neumann, 2018). In addition, OT is involved in the regulation of many physiological functions, such as social behavior, emotion, food intake, and pain perception, and exerts anti-inflammatory, antioxidative, analgesic, and neuroprotective effects in many diseases (Jurek and Neumann, 2018). At present, OT is increasingly recognized as a gastrointestinal hormone or neuropeptide that regulates the function of the gastrointestinal tract. OT is endogenously produced and contained within enteric neurons, and its receptors are widely expressed throughout the digestive tract (Monstein et al., 2004; Ohlsson et al., 2006; Shi et al., 2021). OT has been reported to regulate the development and function of enteric neurons and modulate intestinal inflammation, motility, permeability, and mucosal homeostasis (Welch et al., 2008; Qin et al., 2009; Welch et al., 2014; Chen et al., 2015; Tang et al., 2019; Hollenberg, 2021). Published studies have demonstrated the protective effect of OT on VCR-induced sciatic nerve injury (Erdogan et al., 2020; Zhu et al., 2021). Therefore, we believe that OT may ameliorate VCR-induced gastrointestinal motor dysfunction by protecting the ENS from VCR damage.

In addition to the direct damage of the nervous system, characterized by VCR-induced peripheral neuropathy, the pathological mechanisms of VCR-induced neurotoxicity are also related to changes in ion channel activity, oxidative stress, and inflammation (Tay et al., 2022). VCR treatment leads to oxidative stress in the nervous system, including the sciatic nerve, spinal cord, and brain (Chen et al., 2020; Khan et al., 2021). However, whether VCR causes oxidative stress in the intestine has not been reported yet. The inflammation-related pathways ERK1/2 and p38 MAPK are involved in VCR-induced neuropathy (Shen et al., 2015; Fumagalli et al., 2020; Gao et al., 2021; Khan et al., 2021), and the phosphorylation of ERK 1/2 and p38 MAPK kinases in the spinal cord and colonic myenteric nerve plexus is enhanced following VCR treatment (Shen et al., 2015; Gao et al., 2021; Khan et al., 2021). Oxidative stress and MAPK pathway activation contribute to myenteric neuron loss (McQuade et al., 2016; Gao et al., 2021), which may be correlated with VCR-induced enteric neuron damage. OT suppresses oxidative stress and MAPK pathway activation. It reduces the expression of genes that code the oxidative stress response in the inflammatory gut (Welch et al., 2014), inhibits the LPS-induced activation of ERK 1/2 and p38 MAPK (Yuan et al., 2016), and alleviates cisplatin-induced nephrotoxicity by inhibiting NADPH oxidase and p38 MAPK (Rashed et al., 2011). Accordingly, we hypothesized that OT might protect enteric neurons from VCR-induced injury by inhibiting oxidative stress and MAPK activation. This might be the underlying mechanism by which it alleviates gastrointestinal dysmotility.

## 2 Materials and methods

### 2.1 Experimental chemicals

VCR was ordered from Selleck Chemicals (Houston, TX, United States). OT, carmine red, and carboxymethylcellulose were ordered from Sigma-Aldrich Corp (St Louis, MO, United States). Isoflurane was ordered from RWD (Shenzhen, China). VCR and OT were dissolved and attenuated in normal saline (NS). Carmine red (6%) was suspended in 0.5% carboxymethylcellulose. Primary antibodies against NeuN, β Ⅲ Tubulin, nNOS, choline acetyltransferase (ChAT), Nrf2, p44/42 MAPK, phospho-p44/42 MAPK, p38 MAPK, phospho-p38 MAPK, and GAPDH were ordered from Gene Tex (Irvine, United States), Cell Signaling Technology (Danvers, MA, United States), Abcam (Cambridge, UK), and Proteintech (Chicago, United States). Fluorescent secondary antibodies of Alexa Fluor 568-conjugated donkey anti-rabbit and Alexa Fluor 488-conjugated donkey anti-mouse were ordered from Invitrogen Life Technology (Foster City, CA, United States). DAPI was ordered from Beyotime Biotechnology (Shanghai, China). Dihydroethidium (DHE) was ordered from Sigma-Aldrich Corp. HRP-conjugated goat anti-rabbit secondary antibody was ordered from Zhongshan Golden Bridge Biotechnology (Beijing, China). Stripping buffer was ordered from CWBIO (Taizhou, China).

### 2.2 Experimental animals

Male C57BL/6J mice, aged 8–10 weeks, were ordered from Beijing Vital River Laboratory Animal Technology. The mice were kept in the Animal Center of Shangdong University with natural lighting and unconstrained access to food and water. The Medical Ethics Committee for Experimental Animals of Shandong University approved all the animal experiments (ECSBMSSDU 2020-2-006).

### 2.3 Experimental subgroups

**FIGURE 1:**
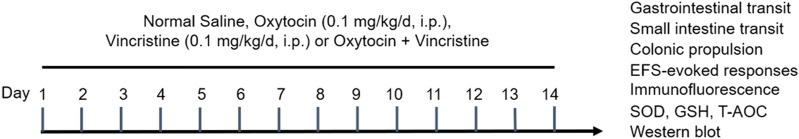
Schematic presentation of the experimental protocol. Mice were injected with normal saline (NS) or vincristine (VCR, 0.1 mg/kg/d, i. p.) for 14 consecutive days. Oxytocin (OT, 0.1 mg/kg/d, i. p.) was administered 1 h before each VCR injection.

The mice were randomly divided into the following four groups (Figure 1):

1) NS group: mice injected with NS (10 mL/kg/d, i. p.) for 14 days
2) OT group: mice injected with OT (0.1 mg/kg/d, i. p.) for 14 days (Zhu et al., 2021)
3) VCR group: mice injected with VCR (0.1 mg/kg/d, i. p.) for 14 days
4) OT + VCR group: mice injected with OT 1 h before each VCR injection

The mice were injected with the drugs between 8 and 11 a.m. and used for different experiments 24 h after the last injection (Supplementary Material S1: Table 1).

**TABLE 1:** Experimental subgroups.

Cohort of animals	Experimental groups	Animal numbers	Experiments
1	4	6/group	Total gastrointestinal transit time and organ-bath study
2	4	8–9/group	Intestinal propulsion rate and colonic propulsion
3	2 (NS and VCR)	4–6/group	Measure of OT concentrations
4	4	4–5/group	β Ⅲ Tubulin immunofluorescence staining and Western blot study
5	4	13–15/group	NeuN, nNOS, and ChAT immunofluorescence staining
6	4	3–4/group	Measure of ROS levels
7	4	4–7/group	Measure of oxidative stress indicators

### 2.4 *In vivo* analysis of gastrointestinal tract motility

#### 2.4.1 Total gastrointestinal transit time

Four groups of mice with six mice in each group were used to measure the total gastrointestinal transit time. At the end of the model, carmine red (6%), which was suspended in 0.5% carboxymethylcellulose, was administered to determine the total gastrointestinal transit time (GITT). The mice were fasted for 12 h prior to the analysis, but water was freely provided. Carmine red (0.2 mL/10 g) was intragastrically administered to each mouse. The GITT was the time interval between the gavage and the first red stool defecation. If no red stool was excreted until the end of the experiment (more than 10 h), the mouse was excluded.

#### 2.4.2 Small intestinal transit

After 12 h of fasting, four groups of mice (eight mice per group) were intragastrically administered the same amount of carmine red and euthanized 30 min later by cervical dislocation. All the intestinal tubes from the pylorus to the caecum were removed immediately. The total length of the small intestine and propulsive distance of carmine red in the intestine were measured without tension, and the percentage of carmine red propulsion distance relative to the total length of the small intestine was calculated and reported as the small intestine propulsion rate.

#### 2.4.3 Colonic propulsion

Four groups of mice (eight to nine mice per group) were anesthetized with isoflurane, and then, a glass ball with a diameter of 3 mm was placed 2 cm from the anus into the distal colon with a smooth glass pole. Subsequently, the mice were kept separately in a cage for observation. The expelling time of the glass ball was documented, which was represented as bead latency.

The GITT and glass ball expulsion time from the colon were reported as the calculated time. The small intestine propulsion rate was documented as the ratio of the carmine red propulsion distance to the length of the small intestine.

### 2.5 Enzyme-linked immunosorbent assay

At the end of the model, the whole blood samples from two groups of mice (four to six mice in the NS and VCR groups) were taken by eyeball extirpating, placed at room temperature for 30 min, and centrifuged at 1,000 *g* for 15 min. The liquid supernatant was carefully pipetted and used to measure the concentration of OT using an enzyme-linked immunosorbent assay (ELISA) kit following the manufacturer’s instructions.

### 2.6 Recording the contractile activity of the colonic segments *in vitro*

After a 12-h fast without food but with water, four groups of mice (six mice per group) were euthanized by cervical dislocation. Approximately 1.5 cm of the distal colon without mesentery was removed. Without removing the mucosa and either muscular layer, each end of the longitudinally mounted bowel segment (equivalent to the longitudinal muscle) was then tied with a thin and inelastic thread. The lower end of the segment was fixed on a hook at the bottom of the bath, and the other end was connected to a tension transducer (JH-2B, Chengdu Instrument Factory, Chengdu, China). The whole bowel segment was soaked in fresh Krebs solution (pH 7.4), which was aerated continuously with carbogen (95% O_2_ + 5% CO_2_) and kept at 37°C using a thermostatic water pump (ZH-Z, Zhenghu Biological Equipment Ltd., Co., Huaibe, China). The ingredients of Krebs solution were as follows (in mmol/L): NaCl 118, KC1 4.8, NaHCO_3_ 25, NaH_2_PO_4_ 1.0, MgSO_4_ 1.2, glucose 11.1, and CaCl_2_ 2.5. The change in the tension of the isolated colonic segments was recorded using a multichannel physiological recorder system (ML785-PowerLab, ADI, Sydney, Australia), and the data were visualized on a computer by Chart 5 software.

#### 2.6.1 Response to electrical field stimulation

The colonic segments were balanced for approximately 1 h at a preload of 1 g (rinsed with fresh Krebs solution at 37°C every 15 min), and the experiment was started after their spontaneous contraction stabilized. Two platinum electrodes connected with a stimulator (SEN-7203, Nihon Kohden, Tokyo, Japan) were placed around the colonic segments. Electrical field stimulation (EFS) with a voltage of 80 V, frequency of 8 Hz, pulse width of 0.6 ms, and duration of 6 s was applied to elicit neural responses. EFS was reduplicated thrice at 10-min intervals.

EFS induced biphasic responses consisting of relaxation and contraction in our experiment. The extent of relaxation and contraction in response to EFS was evaluated by calculating the average of the area under the curve (AUC) after performing EFS thrice. The AUC was reported as g.s. The spontaneous contractions were assessed by calculating the AUC of basal contractile activity within 1 min before any stimulation.

### 2.7 Immunofluorescence staining

#### 2.7.1 Cross sectioning of paraffin-embedded tissues

After modeling, the distal colon tissues were removed from four groups of mice (five to six mice per group) euthanized by cervical dislocation, rinsed with phosphate-buffered saline (PBS), and fixed with 4% paraformaldehyde all night at 4°C. After dehydration, the tissues were embedded in paraffin and dissected into serial coronal slices with a thickness of 4 µm. The paraffin sections were dewaxed in xylol, hydrated in a gradient of alcohol solutions, and then, incubated in sodium citrate buffer for 20 min for antigen retrieval. Nonspecific binding was blocked by incubating the tissues with 10% donkey serum at room temperature for 60 min. Afterward, the solution was replaced with mouse anti-β Ⅲ Tubulin [2G10] (cat: ab78078, 1 μg/mL, Abcam) primary antibody and incubated at 4°C lasting all night. After washing with PBS thrice the following day, the tissues on glass slides were incubated with Alexa Fluor 488-conjugated donkey anti-mouse (cat: A-21202, 1 μg/mL, Invitrogen) fluorescent secondary antibody at room temperature under dark light for 60 min. After rinsing again, the tissue sections were stained with DAPI (cat: C1006, Beyotime) for 5 min at the ambient temperature keeping away from light. Finally, the sections on glass slides were sealed with Antifade Mounting Medium (Beyotime) and viewed with a fluorescence microscope (IX71, OLYMPUS).

#### 2.7.2 Whole-mount myenteric plexus

Myenteric plexus (MP) whole mounts were prepared for immunofluorescence staining to assess the changes in myenteric nervous plexus. The 2-cm distal colons were removed from four groups of mice (thirteen to fifteen mice per group), cleared with cooled Krebs solution, cut longitudinally along the edge of mesentery, and pinned to a Sylgard™-lined dissecting dish. MP was prepared by eliminating the mucosal, submucosal, and circummuscular layers, followed by digestion with papain (10 mg/mL) dissolved in Krebs solution for 50 min at 37°C. Then, the tissue was rinsed thrice with PBS and stretched to twice its size under a microscope. The next step was to fix the preparation with 4% paraformaldehyde for 30 min, followed by rinsing with PBS thrice and blocking with 10% donkey serum for 60 min. These operations were performed at room temperature. After blotting the blocking solution with filter paper, the tissue was incubated with rabbit anti-NeuN [EPR12763] (cat: ab177487, 8.5 μg/mL, Abcam), mouse anti-β Ⅲ Tubulin [2G10] (cat: ab78078, 1 μg/mL, Abcam) and rabbit anti-nNOS [EP 1855Y] (cat: ab76067, 6 μg/mL, Abcam), or mouse anti-β Ⅲ Tubulin [2G10] (cat: ab78078, 1 μg/mL, Abcam) and rabbit anti- ChAT [N1N3] (cat: GTX113164, 6.15 μg/mL, Gene Tex) primary antibodies at 4°C lasting all night. The following day, the tissue was rinsed thrice with PBS and then incubated with the same fluorescent secondary antibodies (Alexa Fluor 488-conjugated donkey anti-mouse or Alexa Fluor 568-conjugated donkey anti-rabbit) at room temperature under dark light for 60 min. After three rinses, the tissue was stained with DAPI for 5 min. Then, the tissue was rinsed again, transferred to and flattened on a glass slide, sealed with Antifade Mounting Medium, and viewed with a fluorescence microscope.

#### 2.7.3 Measurement of reactive oxygen species

DHE was used to assess the production of reactive oxygen species (ROS). Tissue preparation was performed in the same manner as the paraffin sections. The fixed samples of colons from four groups of mice (three to four mice per group) were treated with graded dehydration and then embedded in OCT (Servicebio, China). After that, the tissues were dissected into serial frozen sections with a thickness of 10 µm and stored at −20°C for future use. Frozen sections were taken out and rewarmed at room temperature. Afterward, the colonic sections were incubated with DHE dye (cat: D7008, 5 μg/mL, Sigma-Aldrich) at 37°C under dark light for 30 min, followed by a 5 min rinse with PBS thrice. After water drying, DAPI was applied to the sections for 5 min. Then, the slides were rinsed thrice with PBS, sealed with Antifade Mounting Medium, and viewed under a fluorescence microscope.

In the immunofluorescence assay, the number of β Ⅲ Tubulin-immunopositive neurons in the paraffin section and the number of NeuN-, nNOS-, and ChAT-immunopositive neurons in the MP were measured by counting 5–10 or 6–8 non-overlapping images captured from each MP or paraffin section with a ×40 objective. For ROS levels, the mean fluorescence intensity was analyzed in three non-overlapping images, which were captured with a ×20 objective.

### 2.8 Measurement of superoxide dismutase, glutathione, and total antioxidative capacity

After modeling, the distal colons were isolated from four groups of mice (four to seven mice per group) euthanized by cervical dislocation, rinsed with cold PBS, and stored at −80°C. The activity of superoxide dismutase (SOD), content of glutathione (GSH), and total antioxidative capacity (T-AOC) were examined with assay kits (Nanjing Jiancheng Bioengineering Institute, Nanjing, China) following the manufacturer’s instructions.

### 2.9 Western blot analysis

Distal colon samples from four groups of mice (four to five mice per group) were homogenized with cold RIPA lysis solution (Boster Bio, Pleasanton, CA, United States) including 1% PMSF (protease inhibitor) and 1% phosphatase inhibitor (Boster Bio, Pleasanton, CA, United States), centrifuged at 13,800 *g* for 15 min at 4°C, and the liquid supernatant was gathered for quantitating protein concentration using a BCA assay kit (Boster, United States).

Loading buffer (5×) was added to the supernatants, followed by denaturation via seething at 100°C for 10 min. After that, the proteins were isolated with 12% polyacrylamide gel electrophoresis and then shifted to polyvinylidene difluoride (PVDF) membranes. The membranes were blocked with 5% non-fat milk at room temperature for 60 min and then cut according to the molecular weight of the target protein to incubate with rabbit anti-Nrf2 (cat: ab137550, 1 μg/mL, Abcam), rabbit anti-p44/42 MAPK (ERK1/2) (137F5) (cat: #4695, 0.084 μg/mL, CST), rabbit anti-Phospho-p44/42 MAPK (ERK1/2) (cat: #9101, 0.191 μg/mL, CST), rabbit anti-p38 MAPK (cat: #9212, 0.023 μg/mL, CST), rabbit anti-Phospho-p38 MAPK (cat: #9211, 0.046 μg/mL, CST), and rabbit anti-GAPDH (cat: 10494-1-AP, 0.3 μg/mL, Proteintech) primary antibodies at 4°C lasting all night. On the second day, the membranes were immersed in HRP-conjugated goat anti-rabbit (cat: ZB-2306, 0.2 μg/mL, Zhongshan Golden Bridge Biotechnology) secondary antibody at room temperature for 60 min. After rinsing with TBST buffer thrice, the membranes were exposed to ECL chemiluminescence reagent. Then, immunoreactive bands were visualized with the Tanon Imaging System (Tanon-4600) and analyzed using ImageJ software. The gray values of the two bands of Nrf2 were quantified (Chaudhary et al., 2021; Lan et al., 2021; Lin et al., 2021), but the absence of positive controls or antagonists was a flaw in our methodology. For proteins with close molecular weight, the developed membrane was treated with Stripping Buffer (CWBIO, Taizhou, China) and then re-incubated with new primary antibodies (Litovchick, 2020). The procedure was as follows: first, the membranes were immersed in Stripping Buffer for 30 min at 37°C with shaking to remove the primary and secondary antibodies bound to the membranes; second, they were washed thrice with TBST for 5 min at room temperature; and third, they were blocked again and subsequently incubated with new primary antibodies for the next round of Western blot experiments. The rule of preferentially detecting the target protein with low expression levels (such as phosphorylated proteins) was followed.

### 2.10 Data analysis

All data are expressed as the means ± SEM. Two-tailed Student’s t-test or one-way ANOVA combined with the Newman–Keuls test was used to compare the differences among two or multiple groups. GraphPad Prism version 5 (La Jolla, CA, United States) was used for statistical analysis. *p* < 0.05 was identified as a statistically significant difference.

## 3 Results

### 3.1 OT pretreatment relieves the inhibitory effect of VCR on gastrointestinal transit in mice *in vivo*

The total gastrointestinal transit time, small intestinal propulsion rate, and the time of glass ball discharge from the colon were measured and compared among the four groups of mice to assess global gastrointestinal transit.

#### 3.1.1 OT pretreatment shortens the total gastrointestinal transit time compared with VCR administration alone

**FIGURE 2:**
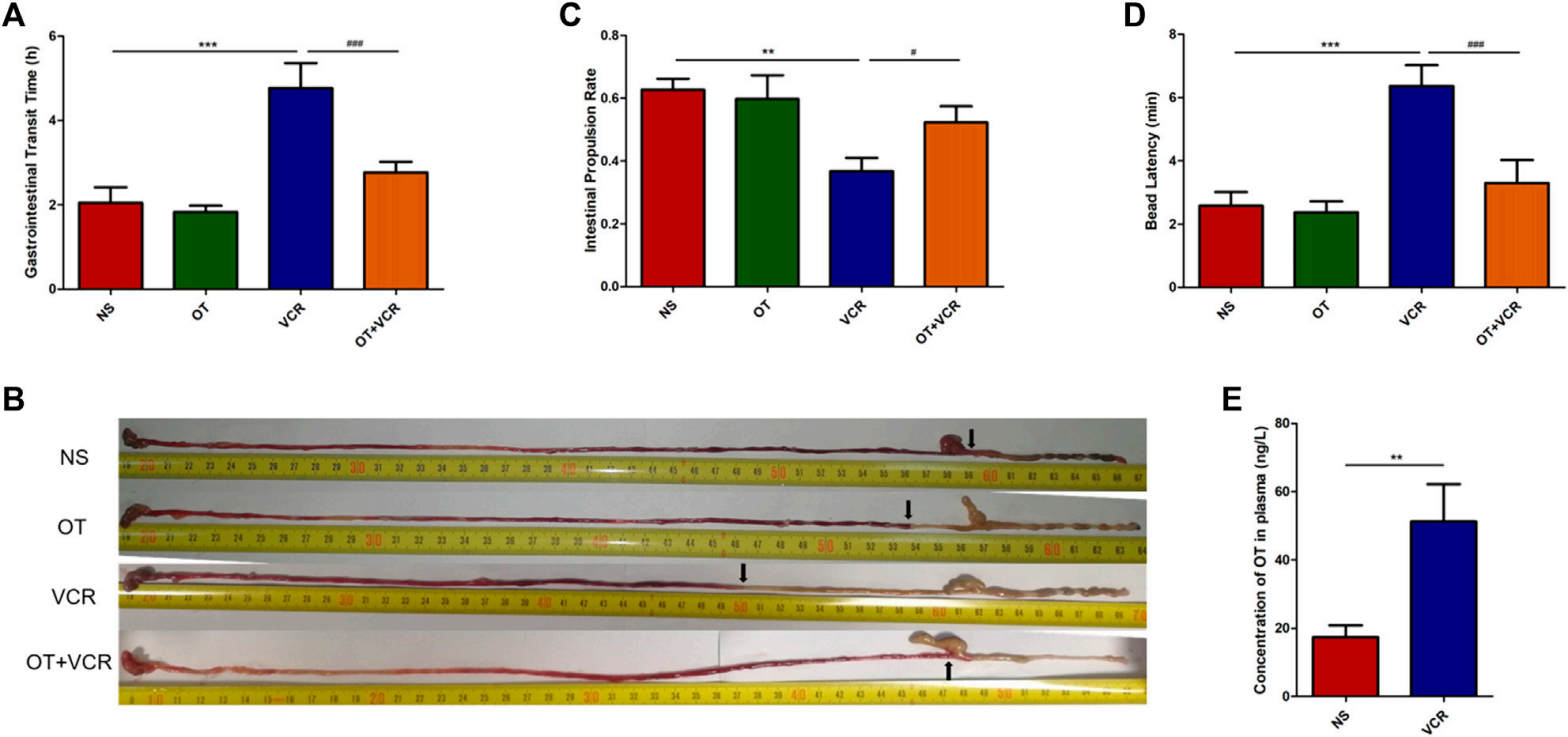
OT relieves the inhibitory effect of VCR on the gastrointestinal transit in mice. **(A)** Total gastrointestinal transit time. Mice were intragastrically administered carmine red, and the time of the first appearance of red feces were recorded (n = 5–6). **(B–C)** Small intestine transit. Mice were intragastrically administered carmine red and sacrificed 30 min later. The small intestine was removed, and the rate of carmine red propulsion was calculated (n = 8). **(B)** Original images of carmine red transport along the intestine. Arrows represent the farthest point at which carmine red is transported along the intestine. **(C)** Histogram shows the small intestinal propulsion rate. **(D)** Colonic propulsion. Mice were anaesthetized with isoflurane, and then, a 3-mm glass ball was placed 2 cm from the anus into the colon with a smooth glass pole. The expelling time of the glass ball was documented (n = 7–9). **(E)** Concentration of OT in plasma was increased in VCR-treated mice as measured by ELISA (n = 4–6). The data are expressed as the means ± SEM, and one-way ANOVA combined with Newman–Keuls or Student’s t-test is used to compare the differences among two or multiple groups. ^*^ *p* < 0.05, ^**^ *p* < 0.01, and ^***^ *p* < 0.001 *versus* the NS group; ^#^ *p* < 0.05 and ^###^ *p* < 0.001 *versus* the VCR group.

One mouse was excluded from the VCR group because it did not excrete red stool at the end of the experiment. The time of the first passage of red stool in the VCR group was 4.77 ± 0.53 h, which was significantly longer than that in the NS control group (2.05 ± 0.33 h) (*p* < 0.01, Figure 2A), and it was shortened to 2.77 ± 0.23 h in the OT + VCR group compared with the VCR group (*p* < 0.01, Figure 2A). This result indicated that OT prevented the slowing of total gastrointestinal transit caused by VCR.

#### 3.1.2 OT pretreatment increases the small intestinal propulsion rate compared with VCR administration alone

There was no significant difference in the length of the small intestine among the four groups (Supplementary Figure S1). The intestinal propulsion rate in mice from the VCR group was 0.37 ± 0.04, which was significantly lower than that of 0.63 ± 0.03 in the vehicle controls (*p* < 0.01, Figures 2B and C). Conversely, it was increased to 0.52 ± 0.05 in mice from the OT + VCR group (*p* < 0.05, Figures 2B and C). This result indicated that OT prevented the weakness of small intestinal propulsion caused by VCR.

#### 3.1.3 OT pretreatment improves the colonic propulsion compared with VCR administration alone

The time of glass ball discharge from the colon was substantially increased from 2.59 ± 0.40 min to 6.36 ± 0.61 min in mice from the VCR group compared with the NS controls (*p* < 0.01, Figure 2D), while it was decreased to 3.30 ± 0.68 min by prior OT administration (*p* < 0.01, Figure 2D). This result indicated that OT prevented the diminution of colonic propulsion caused by VCR.

All the results from the *in vivo* study showed that OT relieved the inhibitory effect of VCR on gastrointestinal transit.

### 3.2 VCR treatment increases the concentration of OT in plasma

The concentration of OT in plasma was measured by ELASA, and the result showed that it was significantly increased in VCR groups (51.20 ± 0.53 ng/L) compared with NS controls (17.32 ± 3.17 ng/L) (*p* < 0.01, Figure 2E). This indicated that VCR promoted the synthesis and secretion of endogenous OT.

### 3.3 OT pretreatment mitigates the inhibitory effect of VCR on neuro-evoked responses in colonic segments *in vitro*

The movement of the digestive tract is mainly controlled by the ENS within the wall and regulated by the central nervous system (CNS) (Furness et al., 2014). To exclude the control of the movement by neural pathways originating outside the alimentary tract, we recorded the contractile activity of colonic segments *in vitro* to detect neurogenic responses by applying EFS to the colon.

**FIGURE 3:**
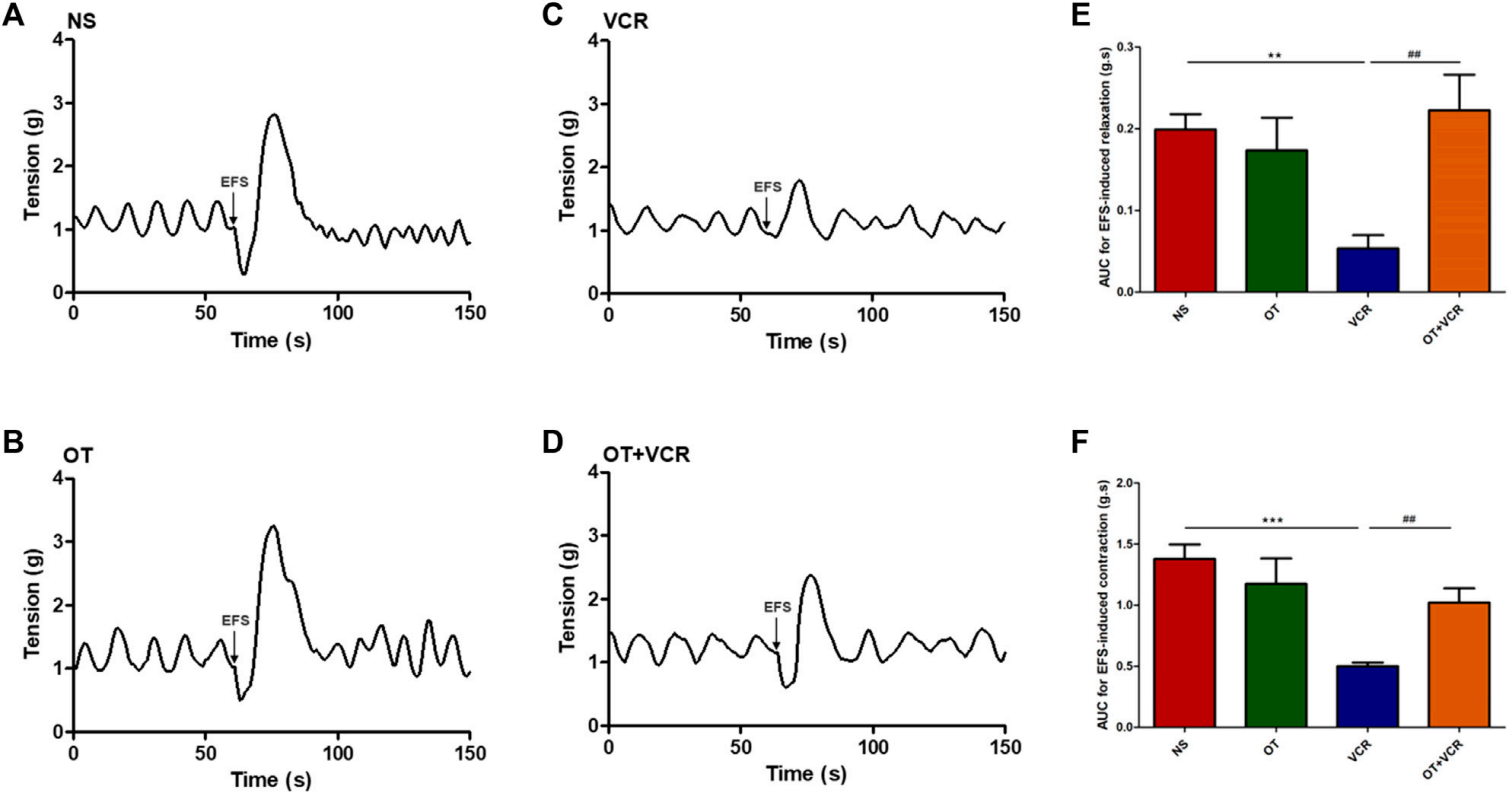
OT mitigates the restrained effect of VCR on electrical field stimulation (EFS)-induced responses of the isolated colonic segments. **(A–D)** Representative tension recordings show the EFS-induced biphasic responses consisting of relaxation and contraction in the four groups of mice: NS **(A)**, OT **(B)**, VCR **(C)**, and OT + VCR **(D)**. Arrows represent the application of EFS. **(E,F)** The area under the curve (AUC) for relaxation **(E)** and contraction **(F)** responses induced by EFS in the four groups indicates that the neurogenic relaxation and contraction of colonic segments are restrained by VCR but eliminated by prior OT treatment (n = 5–6). The data are expressed as the means ± SEM, and one-way ANOVA combined with the Newman–Keuls test is used to compare the differences among multiple groups. ^**^ *p* < 0.01 and ^***^ *p* < 0.001 *versus* the NS group; ^##^ *p* < 0.01 *versus* the VCR group.

In this experiment, the colonic segments showed continuous and stable spontaneous contractions, and no significant difference in the spontaneous contraction of colonic segments was observed among the four groups (Supplementary Figure S2). One mouse was removed from the organ bath study because there was no continuous spontaneous activity. Representative trace from the control showed that EFS induced biphasic responses consisting of relaxation and subsequent contraction (Figure 3A), where nitric oxide (NO) was mainly responsible for the relaxation phase and acetylcholine (Ach) was mainly responsible for the contraction phase (Moralioglu et al., 2017). In the colonic segments from the VCR group, the magnitude of relaxation and contraction in response to EFS were less pronounced than those in the vehicle control group (Figure 3C). The AUC of EFS-induced relaxation was reduced from 0.20 ± 0.02 g s in the NS group to 0.05 ± 0.01 g s in the VCR group (*p* < 0.01, Figure 3E); contraction was reduced from 1.38 ± 0.11 g s to 0.50 ± 0.03 g s (*p* < 0.001, Figure 3F), indicating an inhibitory effect of VCR on EFS-evoked responses. Pretreatment with OT improved the inhibition of VCR on EFS-induced biphasic responses. The magnitude of relaxation and contraction in response to EFS was augmented in the OT + VCR group compared with the VCR group (Figure 3D). Meanwhile, the AUC of relaxation and contraction was also increased to 0.22 ± 0.04 g s and 1.02 ± 0.11 g s, respectively (*p* < 0.01, Figures 3E, F). These findings revealed that OT mitigated the restraint induced by VCR on neurogenic responses.

### 3.4 OT pretreatment reduces VCR-induced injury to myenteric neurons

In the ENS, the myenteric nerve plexus is mainly responsible for the coordination of muscle movements that propel the content (Spencer and Hu, 2020). Therefore, the spatial organization and the number of enteric neurons in the myenteric nerve plexus were studied by immunofluorescence staining.

#### 3.4.1 OT pretreatment increases the numbers of colonic myenteric neurons compared with VCR administration alone

**FIGURE 4:**
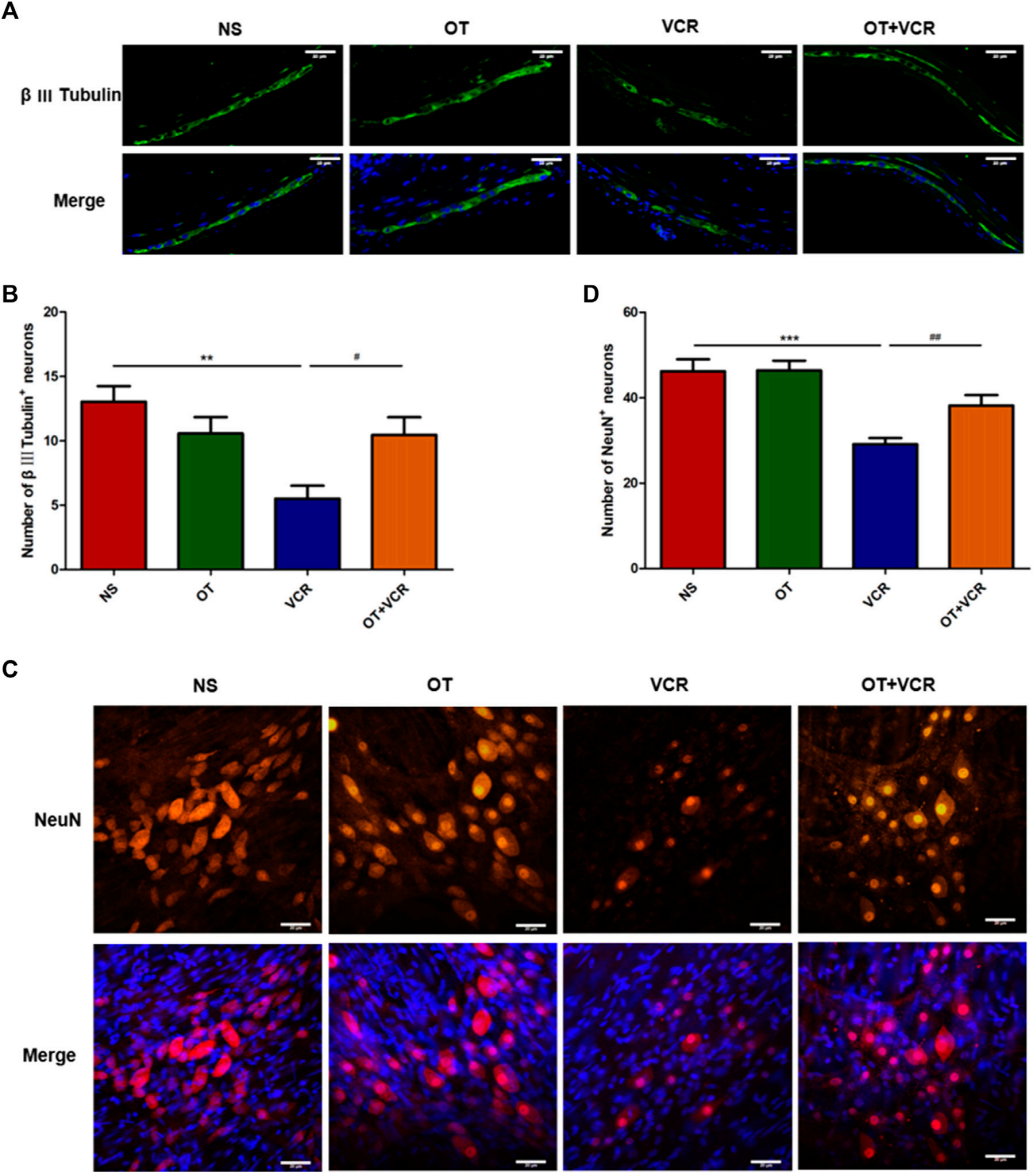
OT attenuates the decrease in the numbers of myenteric neurons induced by VCR administration. **(A)** Typical photo of immunofluorescence staining for β Ⅲ Tubulin in paraffin sections of the colon. **(B)** Quantification of β Ⅲ tubulin-immunopositive neurons (n = 5). **(C)** Typical photo of immunofluorescence staining for NeuN in colonic myenteric nerve plexus. **(D)** Quantification of NeuN-immunopositive neurons (n = 8–9). Scale bars = 20 μm. The data are expressed as the means ± SEM, and one-way ANOVA combined with the Newman–Keuls test is used to compare the differences among multiple groups. ^**^ *p* < 0.01 and ^***^ *p* < 0.001 *versus* the NS group; ^#^ *p* < 0.05 and ^##^ *p* < 0.01 *versus* the VCR group.

Previous results from our lab showed that VCR reduced neuron numbers in the myenteric nerve plexus (Gao et al., 2021), and this result was confirmed by immunofluorescence staining in this present study. From the photomicrographs of the colonic paraffin section and MP, we found that the numbers of β Ⅲ Tubulin (neuronal marker) immunopositive neurons and NeuN (neuronal marker) immunopositive neurons were lower in the VCR group than in the vehicle controls (Figures 4A and C). The numbers of β Ⅲ Tubulin^+^ neurons in the paraffin sections were reduced from 13.03 ± 1.08 in the NS group to 5.96 ± 0.85 in the VCR group (*p* < 0.01, Figure 4B). The amounts of NeuN^+^ neurons in the MP were reduced from 46.18 ± 2.64 to 29.11 ± 1.34 (*p* < 0.001, Figure 4D). In contrast, the amounts of β Ⅲ Tubulin^+^ neurons and NeuN^+^ neurons in the OT + VCR group were greater than that in the VCR group, increasing to 10.45 ± 1.23 and 38.18 ± 2.28, respectively (*p* < 0.05 and *p* < 0.01, respectively, Figures 4B and D). These results suggested that OT prevented the decrease in the amounts of intermuscular plexus neurons caused by VCR administration.

#### 3.4.2 OT pretreatment increases the numbers of nitrergic neurons compared with VCR administration alone in the myenteric nerve plexus

**FIGURE 5:**
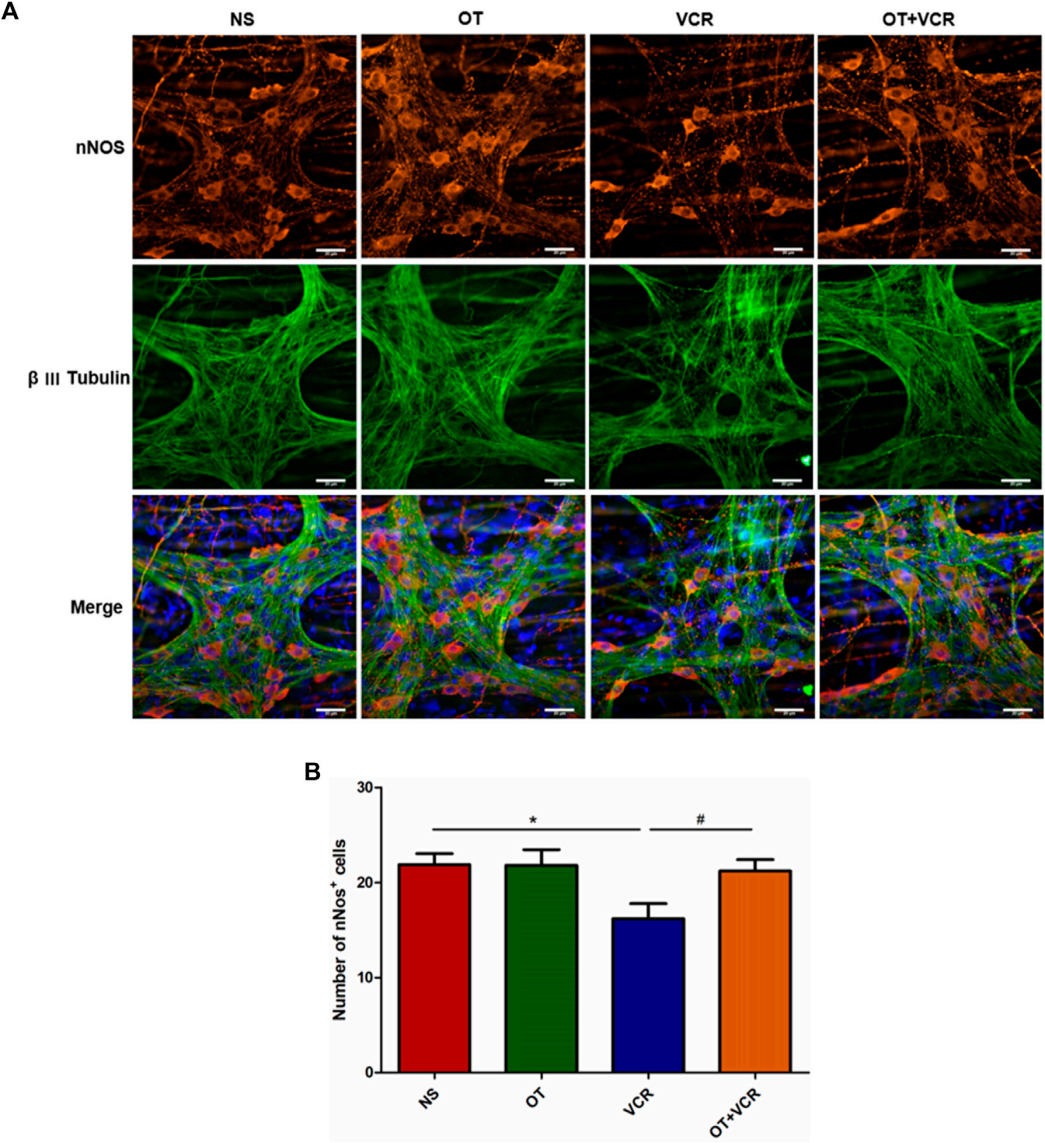
OT pretreatment increases the amounts of nNOS^+^ neurons compared with VCR administration alone in the myenteric nerve plexus. **(A)** Typical photomicrograph of immunofluorescence staining for β Ⅲ Tubulin (green) and nNOS (red) in colonic myenteric plexus. **(B)** Quantification of nNOS-immunopositive neurons (n = 5–7). Scale bars = 20 μm. The data are expressed as the means ± SEM, and one-way ANOVA combined with the Newman–Keuls test is used to compare the differences among multiple groups. ^*^ *p* < 0.05 *versus* the NS group; ^#^ *p* < 0.05 *versus* the VCR group.

NO is a primary inhibitory neurotransmitter generated by the ENS and is mainly responsible for the EFS-induced relaxation phase (Furness, 2000; Moralioglu et al., 2017). Neuronal NO synthase (nNOS) is the main source of NO in the myenteric nerve plexus (Takahashi, 2003). Therefore, the amounts of nitrergic neurons in the colon were detected by double immunofluorescence staining of MP with nNOS (red) and β Ⅲ Tubulin (green). From the representative images and quantitative analysis, we observed fewer nNOS-positive neurons in the VCR group, which was 16.19 ± 1.43, than in the control group, which was 21.87 ± 1.08, but these numbers were increased to 21.21 ± 1.07 in the OT + VCR group (*p* < 0.05, Figure 5).

#### 3.4.3 OT pretreatment increases the numbers of cholinergic neurons compared with VCR administration alone in the myenteric nerve plexus

**FIGURE 6:**
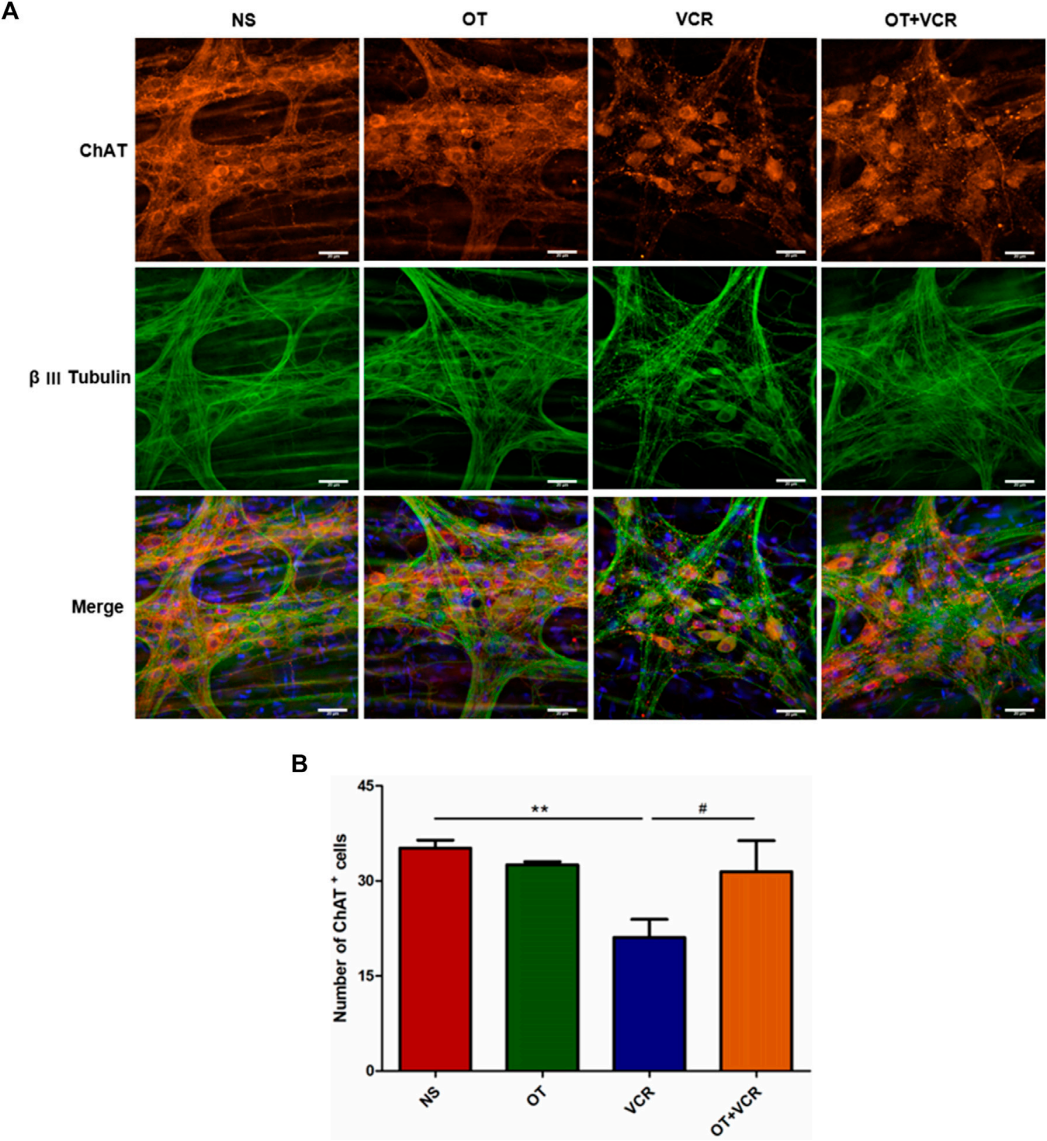
OT pretreatment increases the numbers of ChAT^+^ neurons compared with VCR administration alone. **(A)** Typical photomicrograph of immunofluorescence staining for β Ⅲ Tubulin (green) and ChAT (red) in myenteric plexus of the colon. **(B)** Quantification of ChAT-immunopositive neurons (n = 3–5). Scale bars = 20 μm. The data are expressed as the means ± SEM, and one-way ANOVA combined with the Newman–Keuls test is used to compare the differences among multiple groups. ^**^ *p* < 0.01 *versus* the NS group; ^#^ *p* < 0.05 *versus* the VCR group.

ACh is the major excitatory neurotransmitter produced by motor neurons in the ENS and is mainly responsible for the EFS-induced contraction phase (Furness, 2000; Moralioglu et al., 2017). Therefore, cholinergic neurons in colonic MP were also detected by double immunofluorescence staining for ChAT (red), an ACh synthetase, and β Ⅲ Tubulin. The results showed that the amounts of ChAT-positive neurons were also decreased in the VCR group compared with the NS group, which were reduced from 35.18 ± 1.11 to 21.08 ± 2.49, while this decrease was also prevented by pretreatment with OT, with the numbers of ChAT^+^ neurons being 31.45 ± 4.00 (*p* < 0.01 and *p* < 0.05, respectively, Figure 6).

In conclusion, OT prevented VCR-induced decreases in the amounts of neurons, nitrergic neurons, and cholinergic neurons in the myenteric nerve plexus.

### 3.5 OT attenuates VCR-induced oxidative stress in mice

**FIGURE 7:**
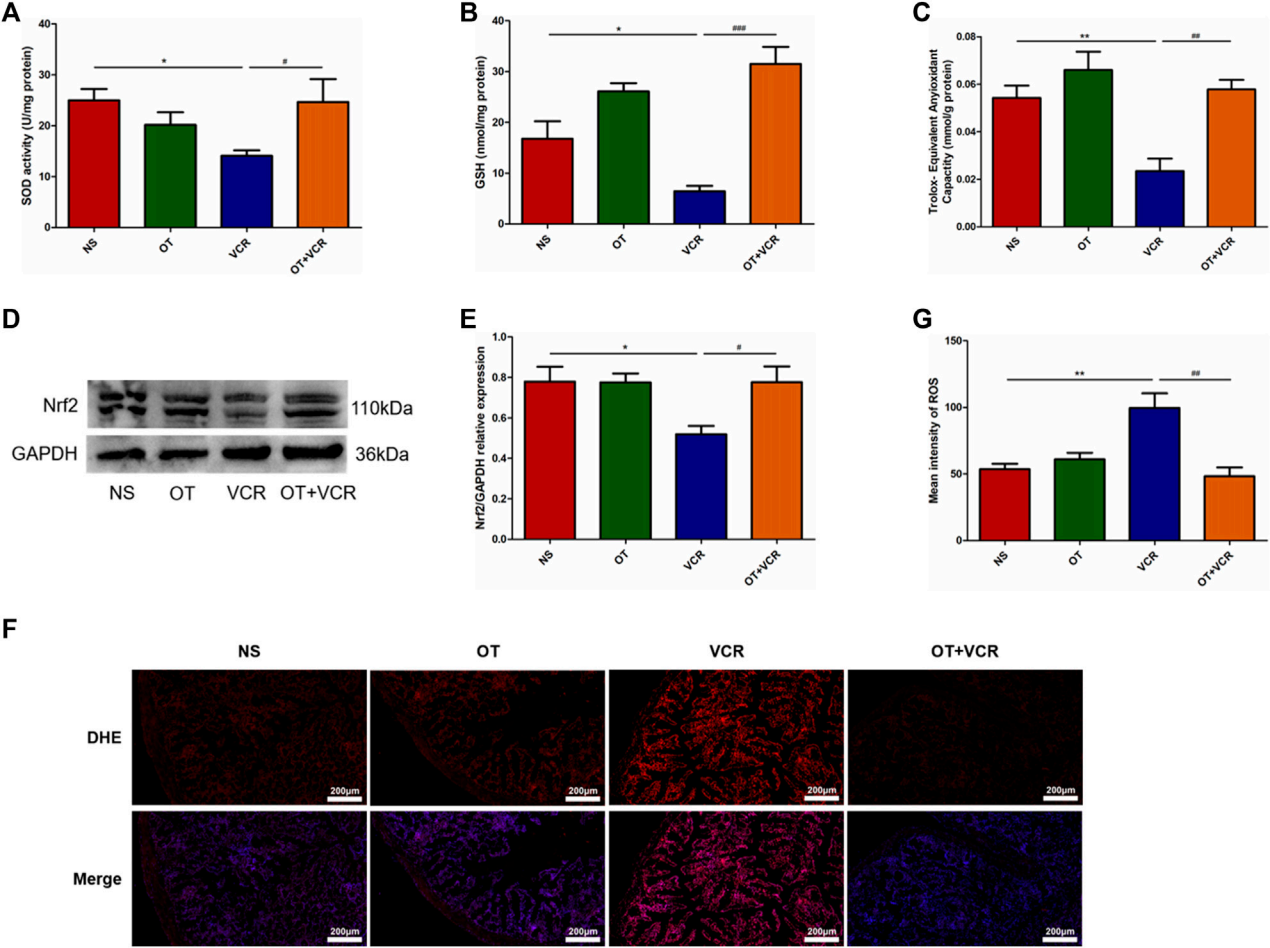
OT attenuates VCR-induced oxidative stress in mice. **(A–C)** Activity of SOD **(A)** and content of GSH **(B)** and T-AOC **(C)** in colon tissues are measured (n = 4–7). **(D)** Representative image of Nrf2 protein is detected by Western blot. **(E)** Quantification of Nrf2 relative expression levels (n = 4). **(F)** Representative image of immunofluorescence staining for DHE in colonic frozen sections. **(G)** Quantification of the mean fluorescence intensity of DHE, which represents for ROS levels (n = 3–4). Scale bars = 200 μm. The data are expressed as the means ± SEM, and one-way ANOVA combined with the Newman–Keuls test is used to compare the differences among multiple groups. ^*^ *p* < 0.05 and ^**^ *p* < 0.01 *versus* the NS group; ^#^ *p* < 0.05, ^##^ *p* < 0.01 and ^###^ *p* < 0.001 *versus* the VCR group.

After VCR treatment, we found that the activity of SOD, the content of GSH, and the T-AOC in the colon samples of mice were significantly reduced (Figures 7A–C). Furthermore, Western blot results showed that Nrf2 protein expression was decreased (Figures 7D and E); the immunofluorescence assay also showed that DHE staining was enhanced and ROS levels were significantly elevated in the VCR group compared with those in the NS group (Figures 7F and G). As previously reported, in this experiment, OT pretreatment increased SOD activity, GSH content, and T-AOC; it also increased Nrf2 protein expression and significantly decreased DHE staining and ROS levels (Figure 7). These results demonstrated that OT attenuated oxidative stress in mice treated with VCR.

### 3.6 OT suppresses ERK1/2 and p38 MAPK activation caused by VCR

**FIGURE 8:**
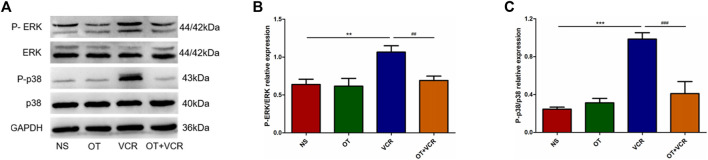
OT suppresses the VCR-induced activation of the ERK1/2 and p38 MAPK pathways. **(A)** Representative image of phosphorylated and total ERK1/2 and p38 protein tested using Western blot. **(B–C)** Quantification of phosphorylated ERK1/2 **(B)** and p38 **(C)** relative expression levels (n = 5). The data are expressed as the means ± SEM, and one-way ANOVA combined with the Newman–Keuls test is used to compare the differences among multiple groups. ^**^ *p* < 0.01 and ^***^ *p* < 0.001 *versus* the NS group; ^##^ *p* < 0.01 and ^###^ *p* < 0.001 *versus* the VCR group.

The results from Western blot have shown that VCR treatment resulted in the activation of ERK1/2 and p38 MAPK pathways, which is manifested by increased phosphorylation levels of the ERK1/2 and p38 proteins. These effects were prevented by OT pretreatment, as indicated by decreased P-ERK1/2 and P-p38 expression (Figure 8). This result suggested that OT inhibited the VCR-induced activation of the ERK1/2 and p38 MAPK pathways.

## 4 Discussion

The effects of OT on VCR-induced gastrointestinal dysmotility and enteric nerve injury were investigated in this study. Continuous VCR treatment for 14 days reduced the intestinal propulsion rate and prolonged the time of glass ball expelled from the colon and the time of first red feces appearance. Furthermore, VCR treatment decreased the relaxation and contraction responses of the colonic segments to EFS and reduced the numbers of total neurons and nNOS- and ChAT-immunopositive neurons in the myenteric nerve plexus. The most exciting finding was that OT improved the gastrointestinal motor function and protected against enteric nerve injury following VCR treatment. OT increased the intestinal propulsion rate, shortened the time of glass ball expelling and first red stool excretion, enhanced the reactivity of colonic segments to EFS, and increased the numbers of myenteric neurons and nNOS- and ChAT-positive neurons. Overall, OT alleviated VCR-induced changes in gastrointestinal motility and neurogenic injury. Further studies showed that OT enhanced the activity of SOD, increased the content of GSH and the T-AOC, increased the expression of Nrf2 protein, and decreased ROS levels; meanwhile, it decreased the phosphorylation levels of ERK1/2 and p38 proteins in the colon samples following VCR treatment. Therefore, OT might play a protective role by inhibiting oxidative stress and the MAPK (ERK1/2 and p38) pathways.

In clinical practice, gastrointestinal motility disorder is a known complication of VCR treatment, presenting as constipation or even paralytic ileus (Tomomasa et al., 1999; Yasu et al., 2016; Escalante et al., 2017; Adil et al., 2021). Animals treated with VCR also develop gastrointestinal motor dysfunction (Sninsky, 1987; Sharma, 1988; Peixoto Júnior et al., 2009; Lopez-Gomez et al., 2018). In the present study, the intestinal transit was altered after VCR treatment. Previous studies by us and other labs had confirmed that OT was an endogenous neuropeptide produced by the ENS (Monstein et al., 2004; Ohlsson et al., 2006; Welch et al., 2008; Shi et al., 2021), which stimulated motor activity in the stomach and colon and improved gastrointestinal dyskinesia induced by stress and hypoxia (Ohlsson et al., 2004; Li et al., 2007; Feng et al., 2009; Qin et al., 2009; Babygirija et al., 2010; Xie et al., 2011; Welch et al., 2014; Yang et al., 2019). Accordingly, we believed that OT might exert protective effects against VCR-induced intestinal transit weakening. This was proved by our results, which showed that OT pretreatment improved intestinal transit. Therefore, clinical preadministration of OT might be useful to prevent or treat intestinal motor dysfunction induced by chemotherapy. In this paper, it was found that OT itself did not affect gastrointestinal transit, possibly because of its short half-life (Smith et al., 2019). The regulatory effects of OT on gastrointestinal motility reported in the pervious literature were usually transient (Ohlsson et al., 2004; Li et al., 2007; Feng et al., 2009; Qin et al., 2009), whereas all experiments in this study were conducted 24 h after the last OT injection. In another study in our lab, administration of OT alone also had no effect on the peripheral pain sensitivity of mice (Zhu et al., 2021).

OT exerts biological effects by binding to its receptor (OTR). OTR is a G-protein-coupled receptor. After combining with OT, it triggers intracellular calcium release and protein kinase C activation through the PLC-IP3/DAG signaling pathway (Blanks et al., 2007; Jurek and Neumann, 2018). We found that OTR on macrophages inhibited the LPS-induced polarization through the β-arrestin 2-NF-κB pathway (Tang et al., 2019). This might be the mechanism underlying the anti-inflammatory effect of OT. Some studies indicated that the anti-inflammatory effect of OT might be physiological. Our previous study found that if the OTR on mononuclear macrophages was conditionally knocked out in mice, the intestinal inflammation induced by DSS was more severe (Tang et al., 2019); Kara Gross Margolis et al. also found that necrotizing enterocolitis was further aggravated after pretreatment with atosiban, an inhibitor of OTR (Gross Margolis et al., 2017). Therefore, as a neuropeptide, OT might exert endogenous inhibition on intestinal inflammation through the OT/OTR signaling system. Abnormal gastrointestinal motility is a known complication in VCR-treated patients (Tomomasa et al., 1999; Yasu et al., 2016; Adil et al., 2021). We found, for the first time, that the treatment of OT exerted a protective effect against VCR-induced gastrointestinal transit weakening. Endogenous OT might have a similar effect, as we found that the concentration of OT in plasma was significantly increased at 14 days following VCR treatment. We had previously reported that the elevated level of OT induced by D-mannose inhibited DSS-induced intestinal injury (Shi et al., 2021; Zhang et al., 2024). Therefore, we speculated that VCR treatment promoted the local synthesis and secretion of OT in the intestine, which in return might have an endogenous protective effect on VCR-induced intestinal transit weakening. This effect might be achieved through OTR.

Patients undergoing chemotherapy with other drugs exhibited structural and functional changes in colonic myenteric neurons (Carbone et al., 2016). VCR also caused the alterations in the ENS (Lopez-Gomez et al., 2018; Gao et al., 2021). Therefore, we speculated that the protective effect of OT against VCR-induced intestinal transit abnormality might be related to the ENS. Immunofluorescence staining of the paraffin sections and MP of the colon showed that VCR reduced the amount of neurons in the myenteric nerve plexus, which was ameliorated by pretreatment with OT. Further studies found that OT also prevented VCR-induced loss of the number of nNOS-immunopositive and ChAT-immunopositive neurons. Based on these results, OT protected the myenteric neurons from VCR-induced damage. This might be the mechanism by which it improved intestinal transit.

The ENS plays a major part in regulating intestinal motility, among which the propulsion of chyme along the oral cavity to the anus is its physiological function. This propulsive movement is attributed to the innervation of the tissue and complex neural reflexes that contract the upstream intestinal muscles (the ascending excitatory reflex) and relax the downstream intestinal muscles (the descending inhibitory reflex), thereby facilitating the propulsive movement of digesta towards the anus (Camilleri, 2021). Excitatory neurotransmitters such as ACh are involved in promoting the ascending excitatory reflex, while inhibitory neurotransmitters such as NO are involved in promoting the descending inhibitory reflex (Lecci et al., 2002). Immunofluorescence results showed that OT successfully prevented the loss of the number of nitrergic and cholinergic neurons caused by VCR. Those two types of neurons are responsible for EFS-induced relaxation and contraction responses, respectively (Moralioglu et al., 2017). In this study, we found that the responses of isolated colonic segments to EFS were attenuated after VCR treatment, suggesting that VCR might inhibit both the responses. Interestingly, OT pretreatment improved EFS-evoked responses and increased the number of nNOS^+^ and ChAT^+^ neurons, suggesting that OT might improve intestinal transit by alleviating VCR-induced neurogenic injuries. Unfortunately, we only examined the neurogenic responses but did not detect any myogenic responses (such as the response of the colonic segment to KCl of the expression of Ach receptors). The present results confirmed that neurogenic factors were involved in the protective effect of OT, but it did not rule out the involvement of myogenic factors. It had been found that VCR affected the response of the colon or the dimensions (Peixoto Júnior et al., 2009; Vera et al., 2017; Lopez-Gomez et al., 2018; López-Tofiño et al., 2023). OT receptors were expressed on smooth muscles cells (Qin et al., 2009), so OT might also exert a protective effect by directly acting on smooth muscles.

Lopez-Gomez L et al. found that VCR increased the proportion of nNOS-positive neurons (Lopez-Gomez et al., 2018). Our results might not contradict this, as an increase in the proportion of nNOS-positive neurons was accompanied by a decrease in their number (McQuade et al., 2016). Unfortunately, due to the defects of our methodology, the labeling of β Ⅲ Tubulin antibody failed to well expose all neurons encapsulated by nerve fibers in MP preparation, so we could only count the nNOS immunoreactive neurons in the current method and could not calculate the proportion. Meanwhile, we also observed a decrease in EFS-induced relaxation such that the function of the nitrergic neurons in smooth muscle relaxation function was weakened. This weakening might be related to the disrupted gastrointestinal motility observed in the clinic.

VCR-induced neuropathy has been reported to be associated with oxidative stress. After nerve injury, antioxidant levels were reduced and ROS levels were increased in the sciatic nerve, spinal cord, and brain (Chen et al., 2020; Zhang and Xu, 2020; Khan et al., 2021). Oxidative stress contributes to the loss of myenteric neurons in individuals with chemotherapy-induced gastrointestinal dysfunction (McQuade et al., 2016). Therefore, we suggested that VCR also induced oxidative stress in the gut, which might be responsible for the reduction of myenteric neurons. We examined the expression of oxidative stress-related indicators in colon tissues and found that VCR reduced SOD activity, GSH content, T-AOC, and Nrf2 protein expression and increased ROS levels in the colon; thus, VCR might cause enteric nerve injury by inducing oxidative stress. The anti-tumor mechanism of VCR is through binding to tubulin, inhibiting microtubule function, arresting mitotic progression, and preventing cell division (Triarico et al., 2021). As an intense site of cell division, the intestinal mucosal epithelium may be one of the targets of VCR. It had been reported that the intestinal mucosal epithelium was damaged following VCR treatment. The number of necrotic cells increased, and metaphase mitotic arrest appeared in the intestinal crypts after VCR treatment, along with villus shortening and even mucosal erosion (Hobsonii et al., 1974; Beró and Jávor, 1985; Lopez-Gomez et al., 2018). These effects might disrupt the intestinal barrier function, facilitate the translocation of bacteria or toxins to intestine, and induce inflammation responses (Chelakkot et al., 2018). It was found that long-term VCR treatment led to inflammatory cells infiltration in intestinal lamina and submucosa (Beró and Jávor, 1985; Lopez-Gomez et al., 2018) and promoted the polarization of intestinal macrophages towards pro-inflammatory phenotype, which increased the release of inflammatory factors (Gao et al., 2021). It is well known that ROS production is increased during the inflammation response. So, it is possible that the VCR-induced increase of intestinal ROS might be caused by local inflammation. OT has been reported to inhibit inflammation and ROS production. It suppressed the elevation of ROS levels in H9c2 cardiomyocytes induced by ischemia–reperfusion (Gonzalez-Reyes et al., 2015), and it was involved in maintaining the intestinal epithelial barrier and restoring the intestinal epithelial injury induced by 5-FU, irradiation, and DSS (Welch et al., 2014; Chen et al., 2015). It alleviated necrotizing enterocolitis, TNBS- and DSS-induced colitis, and food allergy-induced intestinal inflammation and inhibited the expression of genes involved in oxidative stress response in the inflammatory intestine (Old et al., 2014; Welch et al., 2014; Gross Margolis et al., 2017; Tang et al., 2019; Dou et al., 2021; Yu et al., 2022). In this study, OT pretreatment reversed VCR-induced oxidative stress, manifested as the increase of SOD activity, GSH content, T-AOC, and Nrf2 protein expression and the decrease of ROS levels. This might result from the direct inhibition of intestinal ROS production by OT or the indirect reduction of ROS by alleviating intestinal epithelial damage and inflammation. Consequently, OT might exert a protective effect on VCR-induced myenteric neuron injury by ameliorating oxidative stress. Several chemical and natural compounds with antioxidant activity mitigated VCR-induced neuropathy, such as mitoquinone, a mitochondrial-targeted antioxidant, and curcumin, isolated from *Curcuma longa* (Babu et al., 2015; Chen et al., 2020). GSH had also attracted the interest of research scholars in different disciplines, for example, enzyme mechanisms, drug metabolism, radiation, and cancer, due to its multifunctional properties (Gul et al., 2000).

Previous results from our lab showed that VCR induced myenteric neuron injury by enhancing ERK1/2 and p38 MAPK phosphorylation to stimulate macrophage polarization to the pro-inflammatory phenotype, resulting in an increase in inflammatory cytokines (Gao et al., 2021). OT inhibited the LPS-induced activation of ERK1/2 and p38 MAPK in microglia (Yuan et al., 2016). OT inhibited the polarization of macrophages toward the pro-inflammatory phenotype, decreased the release of inflammatory factors, and alleviated intestinal inflammation (Tang et al., 2019). It was hinted that OT might improve VCR-induced myenteric neuron injury by inhibiting the activation of the ERK1/2 and p38 MAPK pathways. Western blot analysis showed that OT reduced the phosphorylation of ERK1/2 and p38 proteins caused by VCR, indicating that OT inhibited the VCR-induced activation of the ERK1/2 and p38 MAPK pathways. Inhibition of Ras and c-Raf1, which were the key elements in the activation of intracellular signal transduction pathways relating to the MAPK family, by farnesyl thiosalicylic acid and GW5074 had been reported to attenuate VCR-induced neuropathy (Jaggi and Singh, 2012).

This paper documented a number of changes that accompanied VCR toxicity. Although OT pretreatment might ameliorate VCR-induced toxicity by inhibiting oxidative stress and MAPK pathways (ERK 1/2, p38), unfortunately, the detailed mechanism was not explored here.

In summary, OT can protect the enteric neurons against VCR-induced damage by inhibiting oxidative stress and the MAPK pathways (ERK 1/2, p38), which may be the underlying mechanism for relieving gastrointestinal dysmotility, but a more precise mechanism remains to be elucidated. Our study provides a basis for the search and development of drugs that may prevent or relieve the constipation or ileal injury caused by VCR, and potentially, OT will be used as a clinical treatment in the future.

## Data Availability

The original contributions presented in the study are included in the article/Supplementary Material; further inquiries can be directed to the corresponding authors.

## Abbreviations

ACh, acetylcholine; AUC, area under the curve; ChAT, choline acetyltransferase; CNS, central nervous system; DAG, diacylglycerol; DAPI, 4′,6′diamidino-2-phenylindole dihydrochloride hydrate; DHE, dihydroethidium; EFS, electrical field stimulation; ELISA, enzyme-linked immunosorbent assay; ENS, enteric nervous system; GITT, gastrointestinal transit time; GSH, glutathione; IP3, inositol-3-phosphate; MP, myenteric plexus; MAPK, mitogen-activated protein kinase; NADPH, nicotinamide adenine dinucleotide phosphate; nNOS, neuronal NO synthase; NO, nitric oxide; NS, normal saline; Nrf2, nuclear factor erythroid 2-related factor 2; OT, oxytocin; OTR, oxytocin receptor; PBS, phosphate-buffered saline; PLC, phospholipase C, PMSF, phenylmethylsulfonyl fluoride; PVDF, polyvinylidene difluoride; ROS, reactive oxygen species; SOD, superoxide dismutase; T-AOC, total antioxidative capacity; and VCR, vincristine.
