# Supplementary material for: Protective effect of oxytocin on vincristine-induced gastrointestinal dysmotility in mice
Source: Front Pharmacol. 2024 Apr 9;15:1270612. doi: 10.3389/fphar.2024.1270612 (PMC11037254; doi:10.3389/fphar.2024.1270612)
Supplement: Supplementary file 2 [file DataSheet8.zip › Fig 8 original data/Fig 8A/Fig 8A.docx]

Full scan of the original blots of cropped images shown in Figure 8A.

Lane1: marker

Lane2: NS

Lane3: OT

Lane4: VCR

Lane5: OT+VCR

P-ERK (44/42kDa)


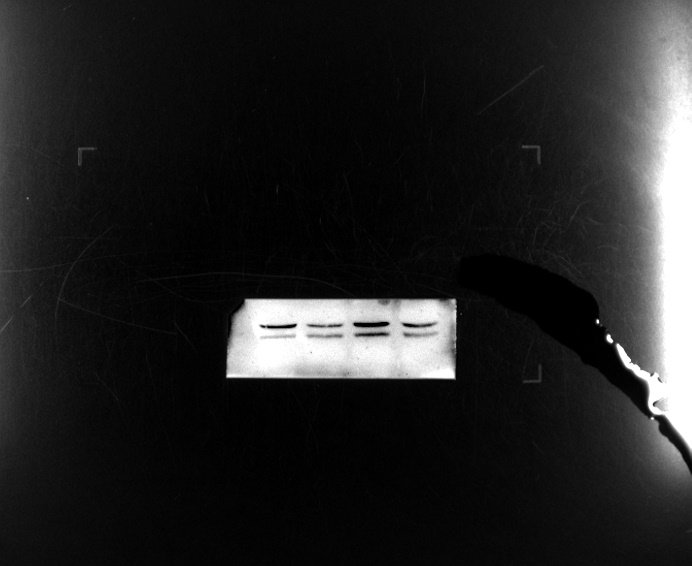


40kDaa

ERK (44/42kDa)


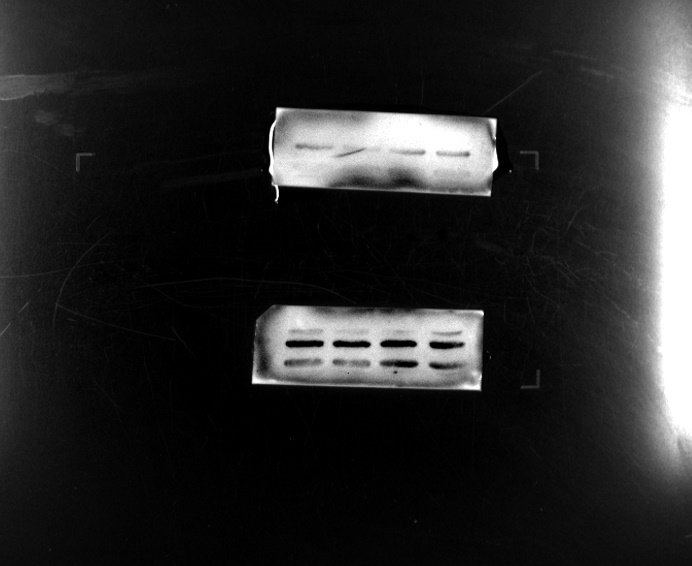


40kDa

P-p38 (43kDa)
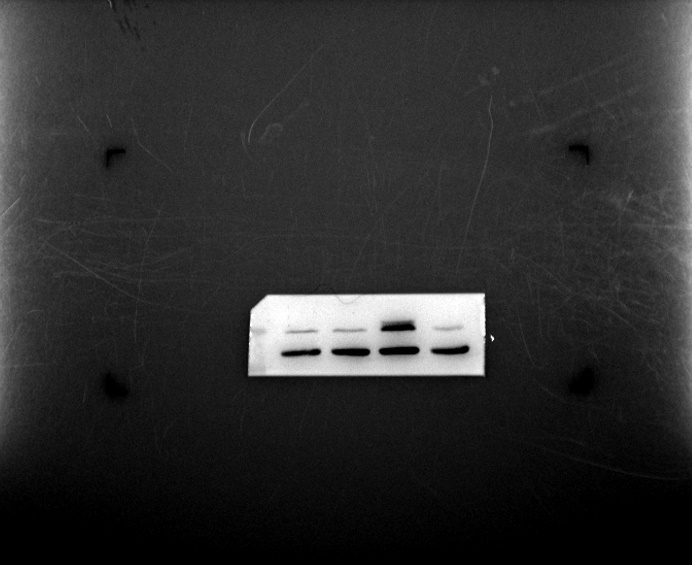


40kDa

P38 (40kDa)
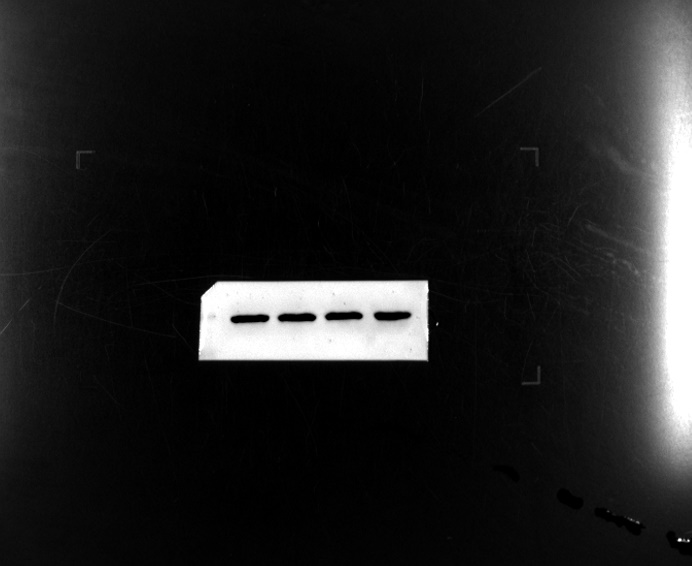


40kDa

GAPDH (36kDa)


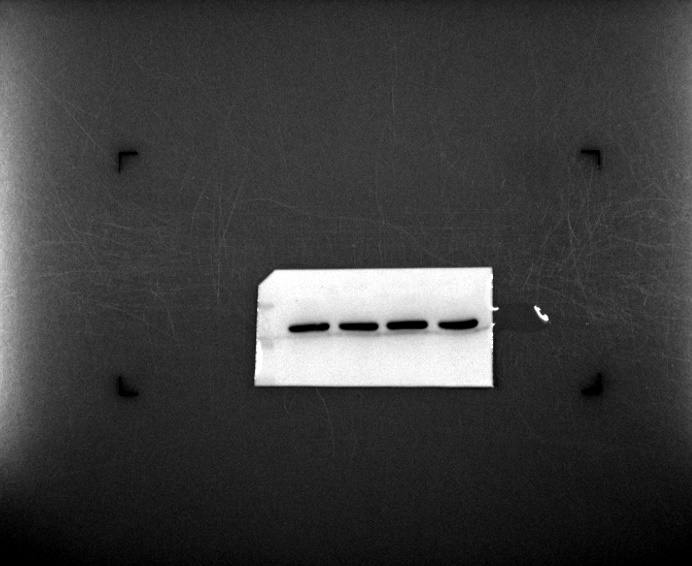


40kDa

35kDa
