# Supplementary figures and images for: Protective effect of oxytocin on vincristine-induced gastrointestinal dysmotility in mice
Source: Front Pharmacol. 2024 Apr 9;15:1270612. doi: 10.3389/fphar.2024.1270612 (PMC11037254; doi:10.3389/fphar.2024.1270612)

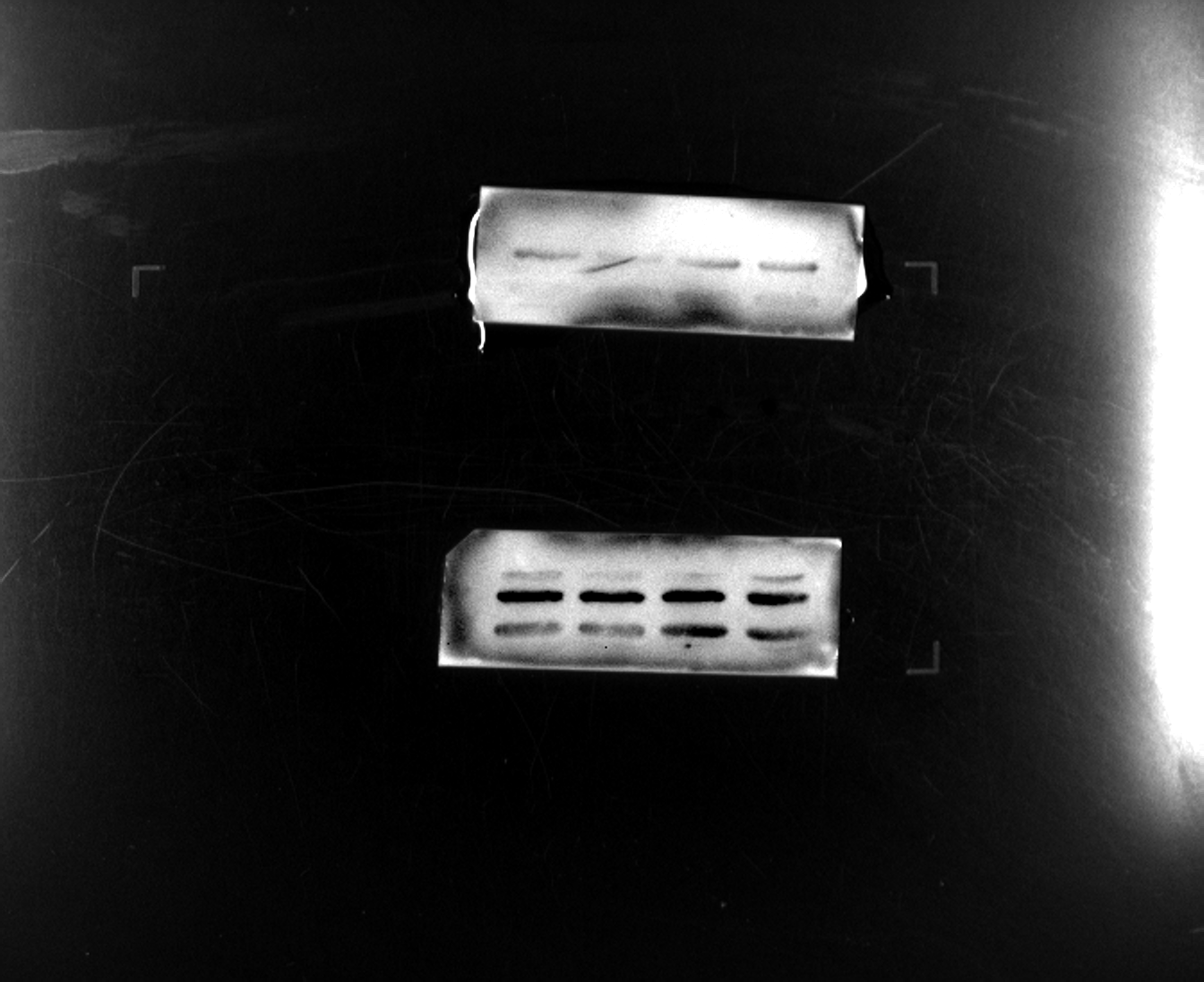

Supplement: Supplementary file 2 [file DataSheet8.zip › Fig 8 original data/Fig 8A/ERK.Tif]

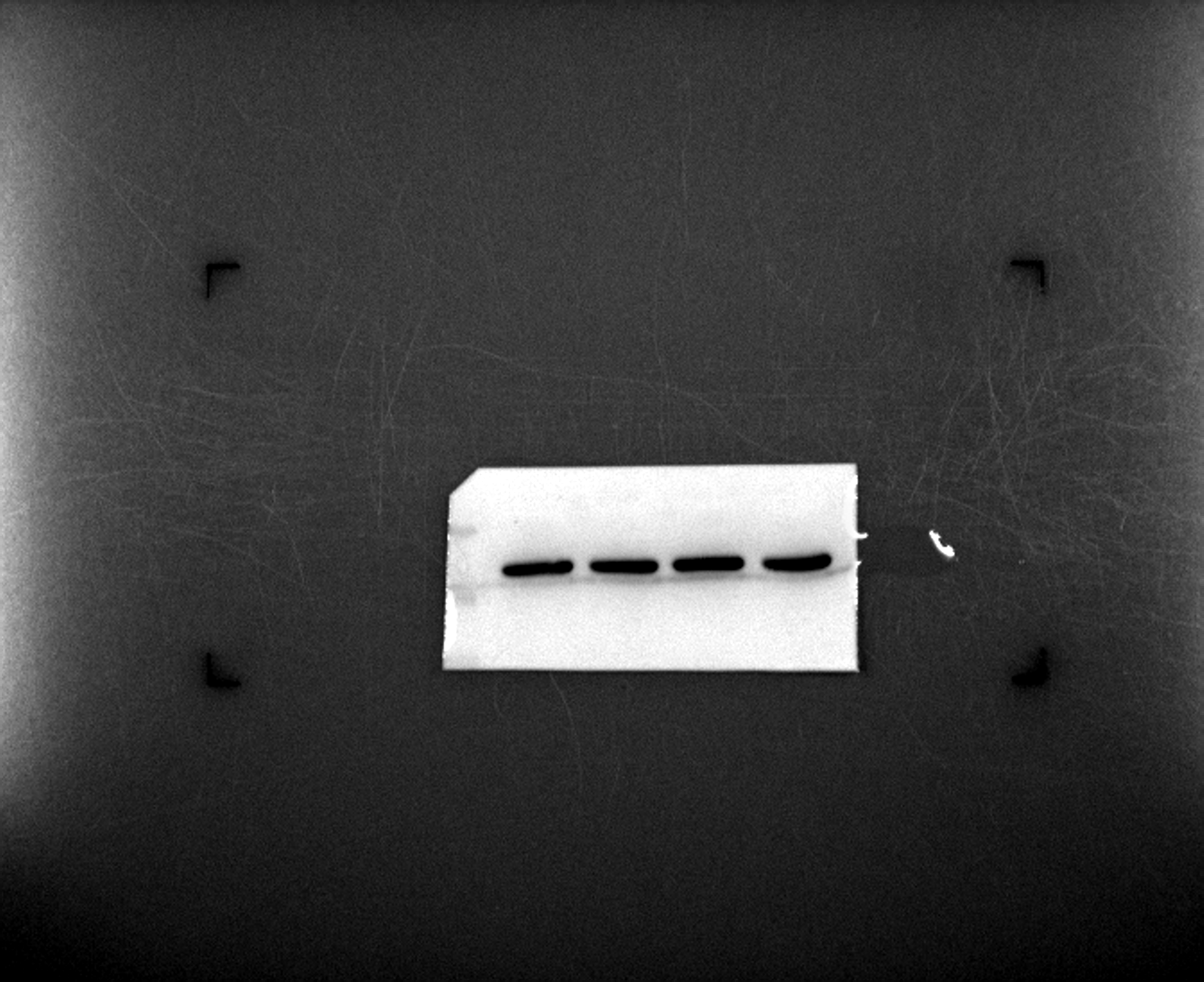

Supplement: Supplementary file 2 [file DataSheet8.zip › Fig 8 original data/Fig 8A/GAPDH.Tif]

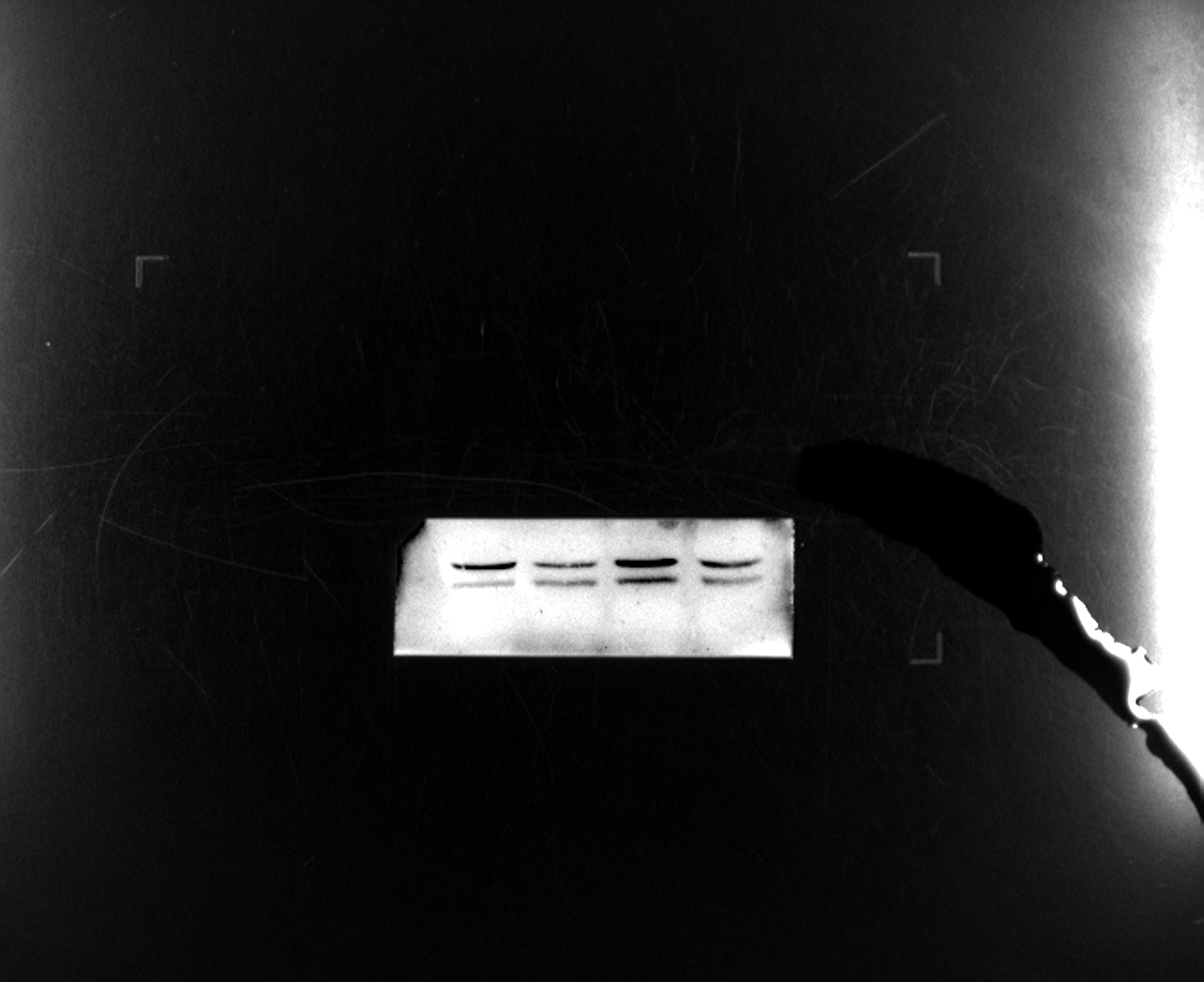

Supplement: Supplementary file 2 [file DataSheet8.zip › Fig 8 original data/Fig 8A/P-ERK.Tif]

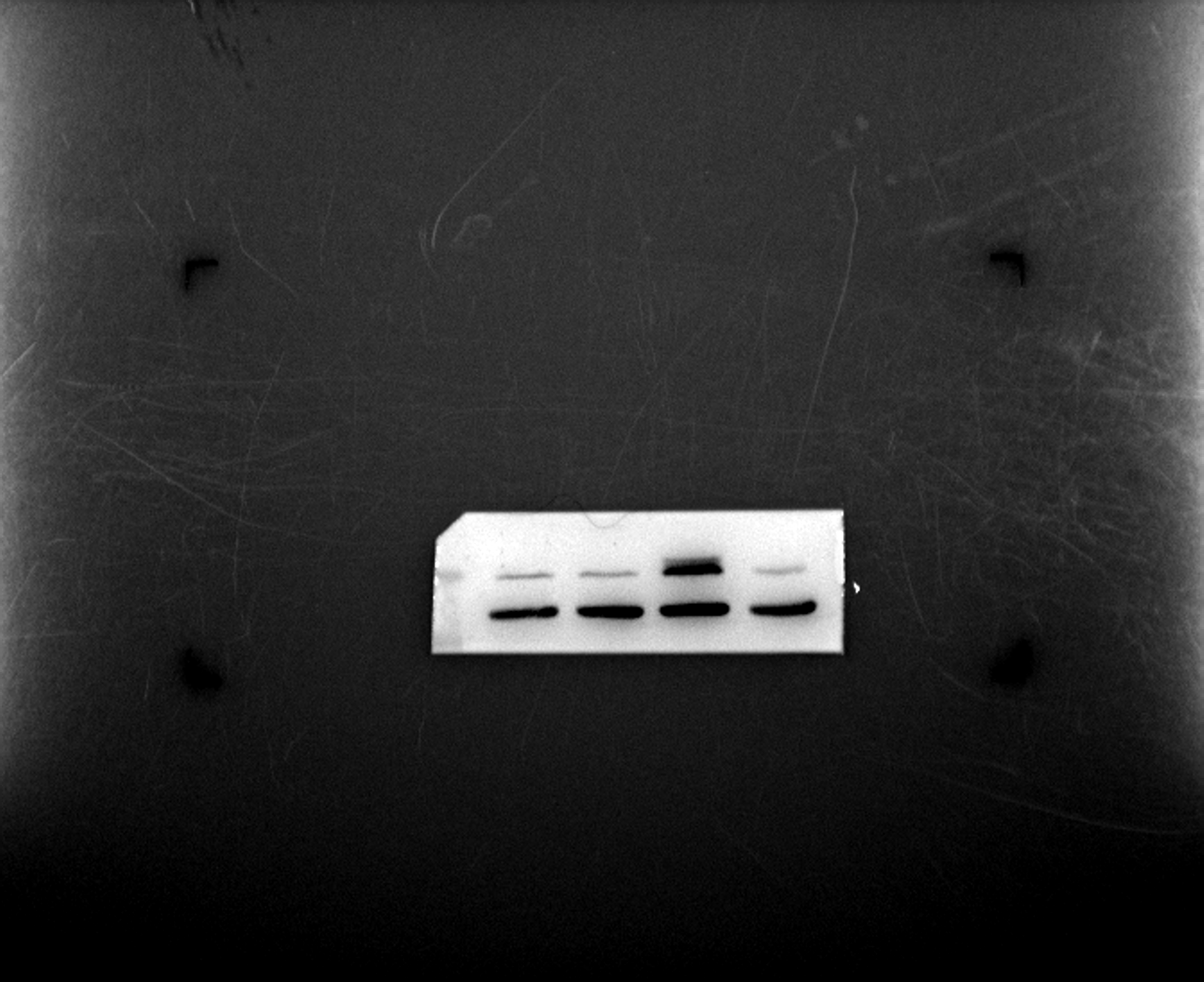

Supplement: Supplementary file 2 [file DataSheet8.zip › Fig 8 original data/Fig 8A/P-p38.Tif]

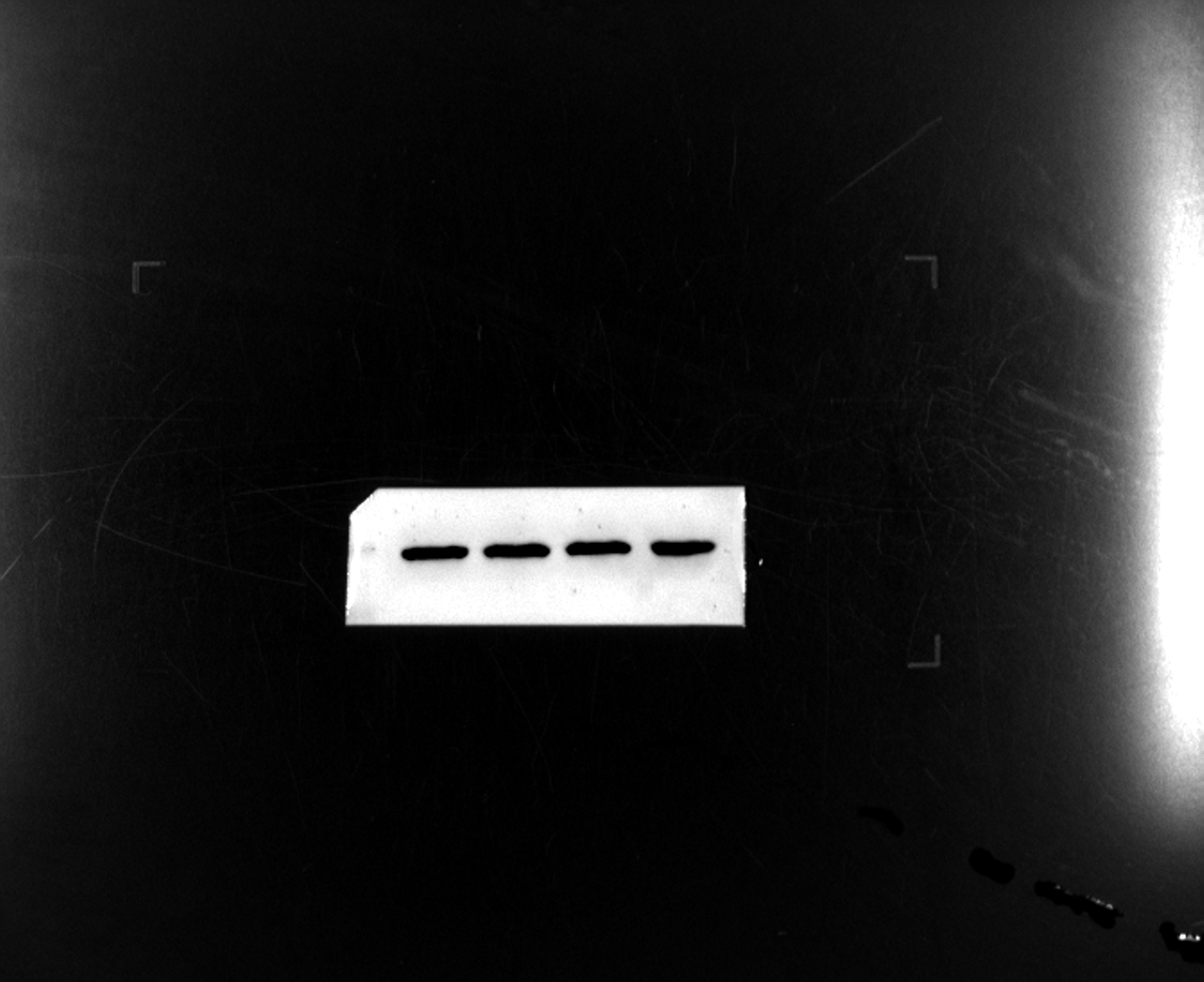

Supplement: Supplementary file 2 [file DataSheet8.zip › Fig 8 original data/Fig 8A/p38.Tif]

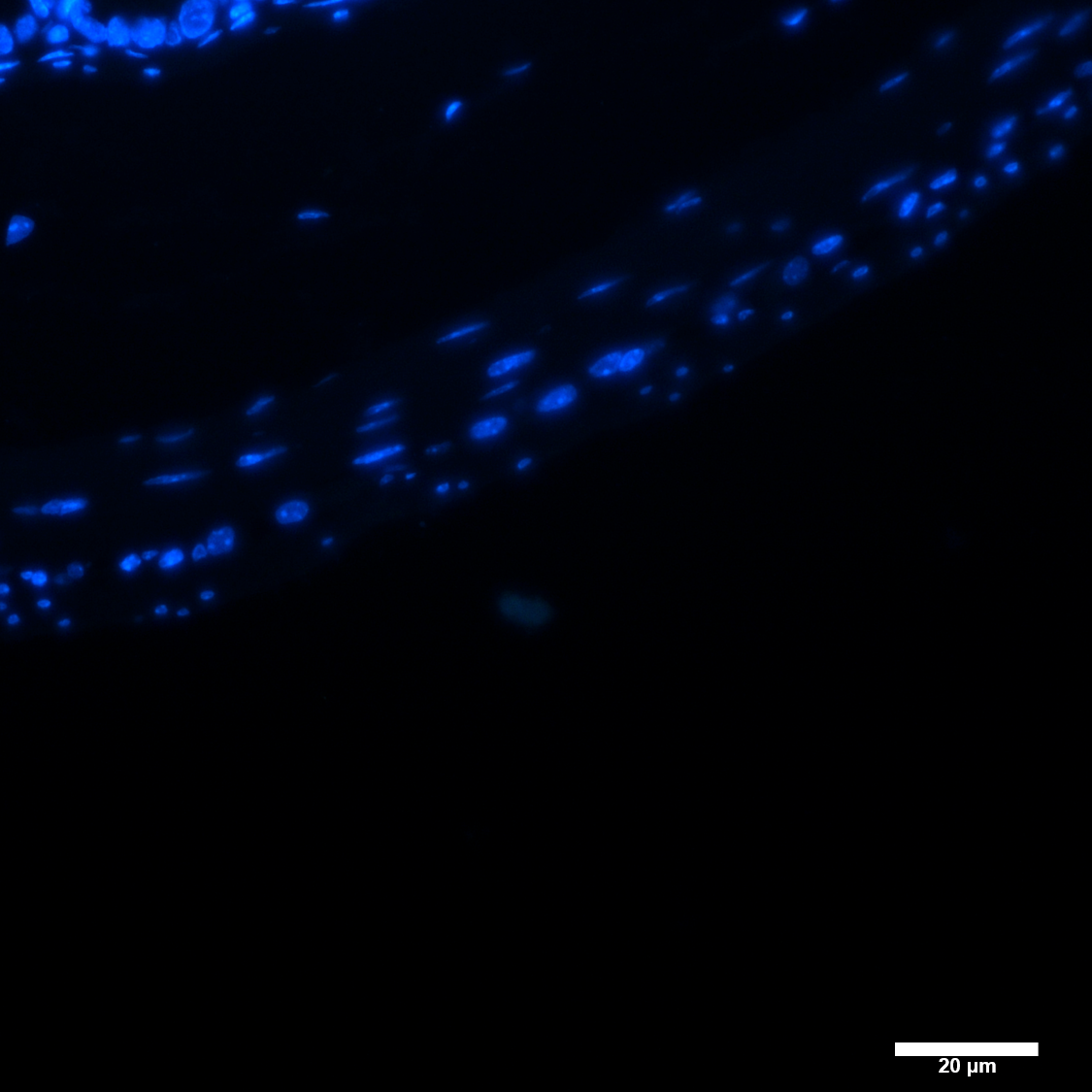

Supplement: Supplementary file 4 [file DataSheet4.zip › Fig 4 original data/Fig 4A/NS-DAPI.tif]

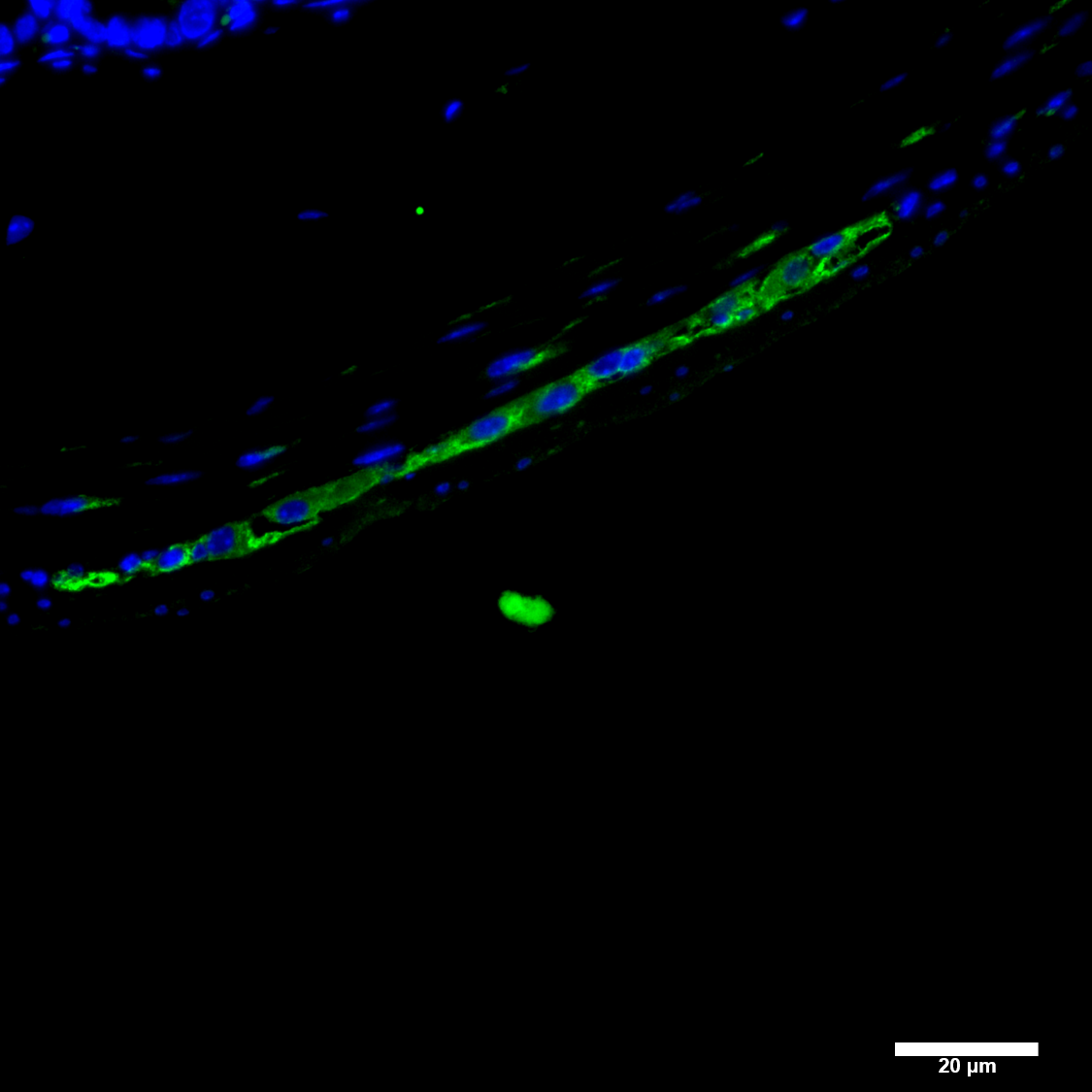

Supplement: Supplementary file 4 [file DataSheet4.zip › Fig 4 original data/Fig 4A/NS-Merge.tif]

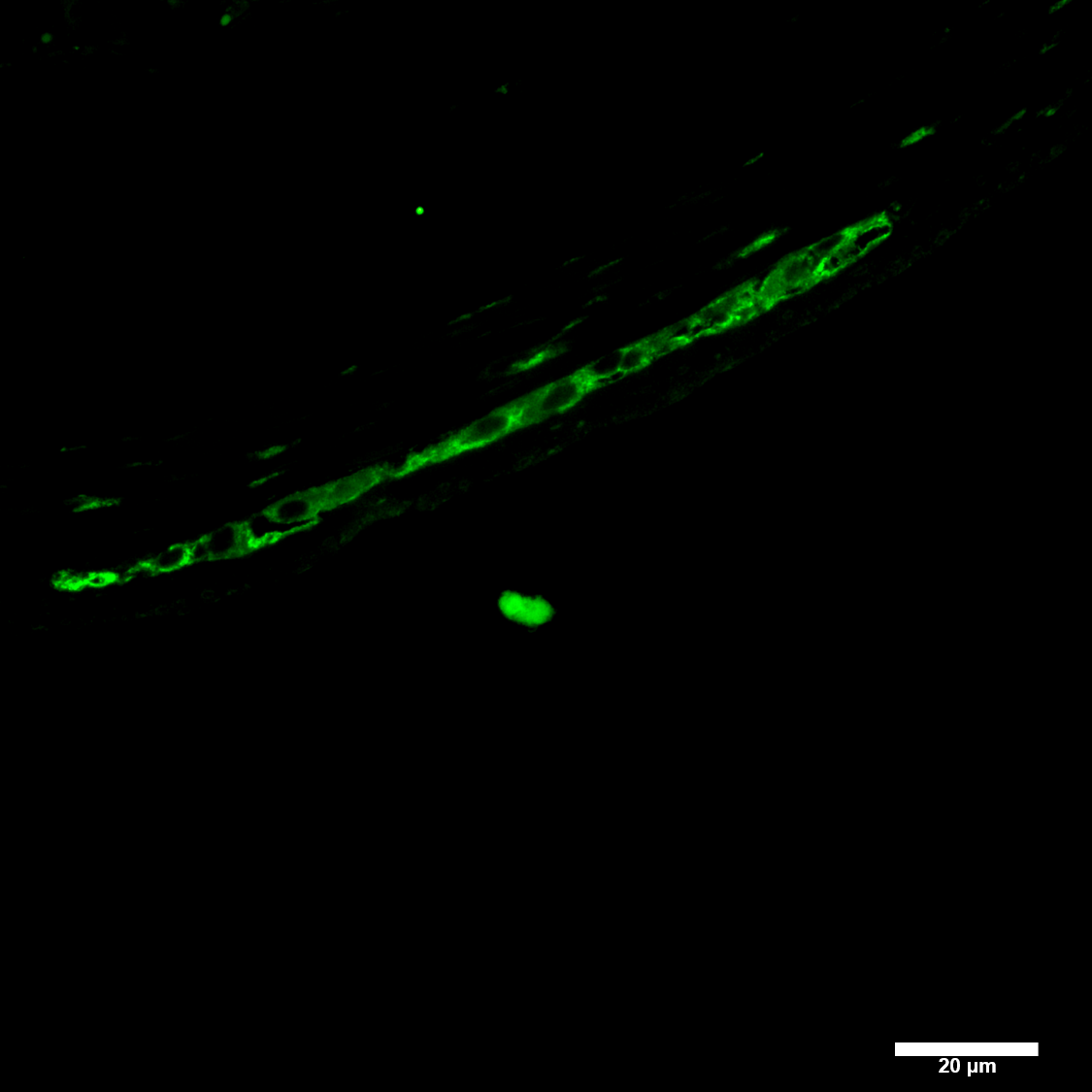

Supplement: Supplementary file 4 [file DataSheet4.zip › Fig 4 original data/Fig 4A/NS-β III Tubulin.tif]

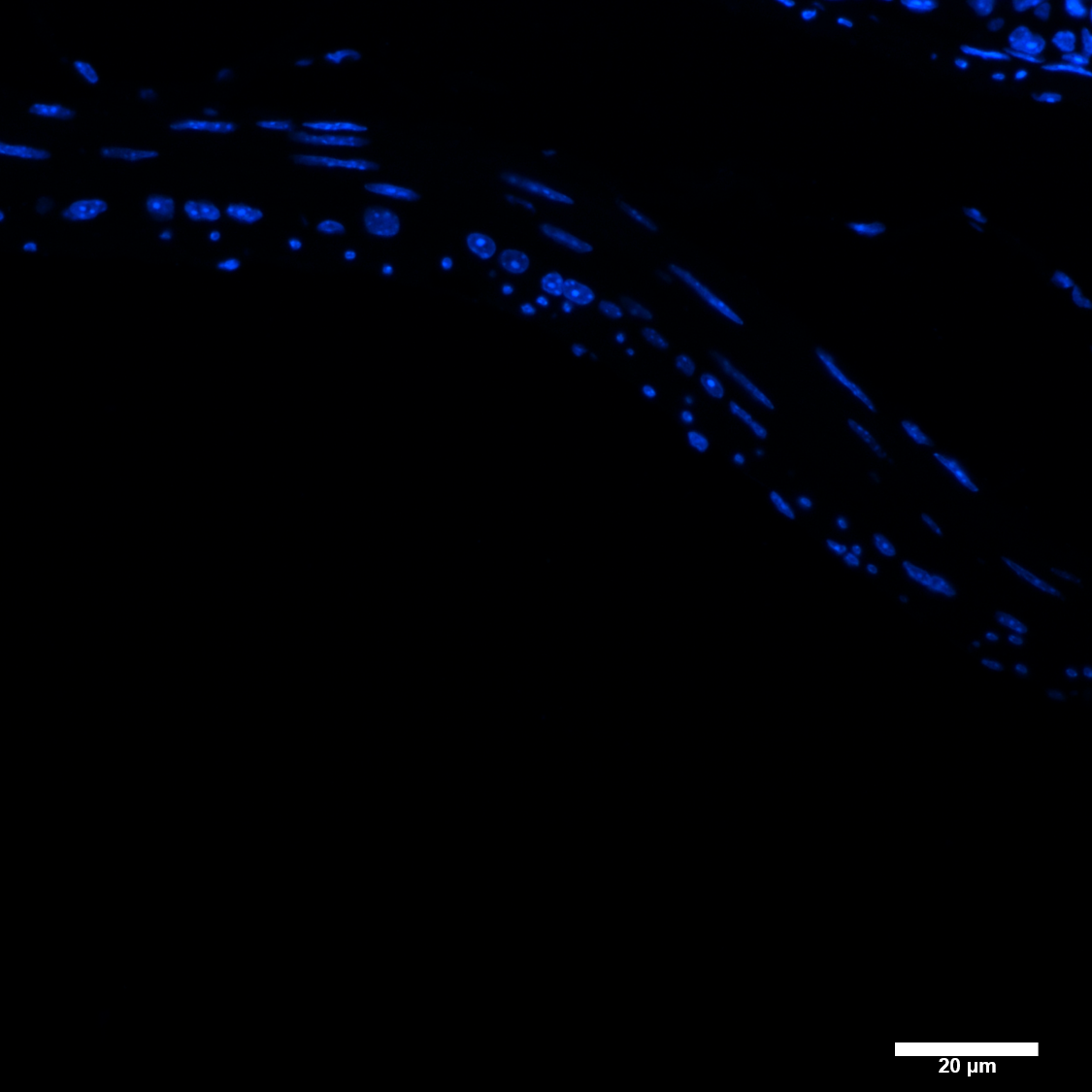

Supplement: Supplementary file 4 [file DataSheet4.zip › Fig 4 original data/Fig 4A/OT+VCR-DAPI.tif]

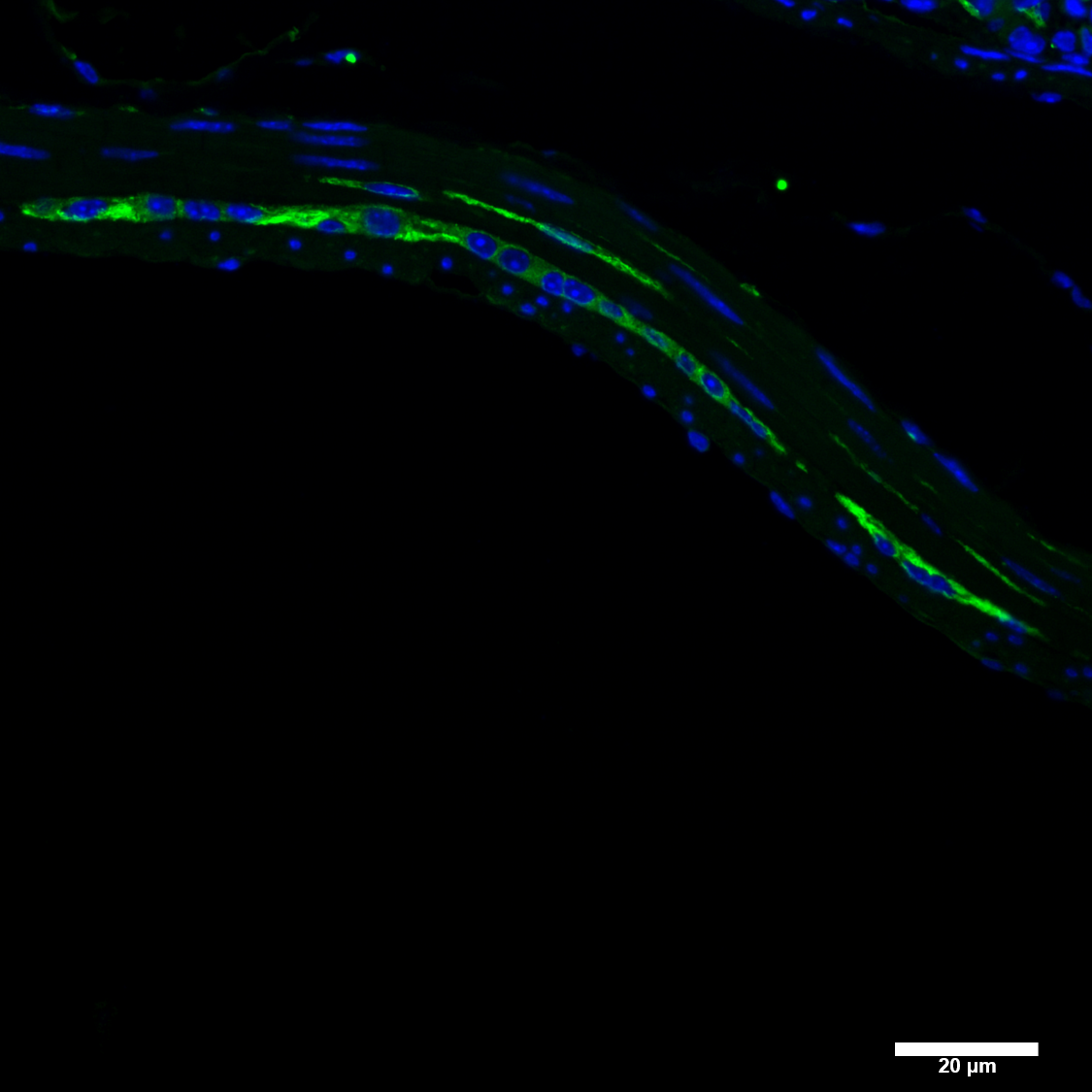

Supplement: Supplementary file 4 [file DataSheet4.zip › Fig 4 original data/Fig 4A/OT+VCR-Merge.tif]

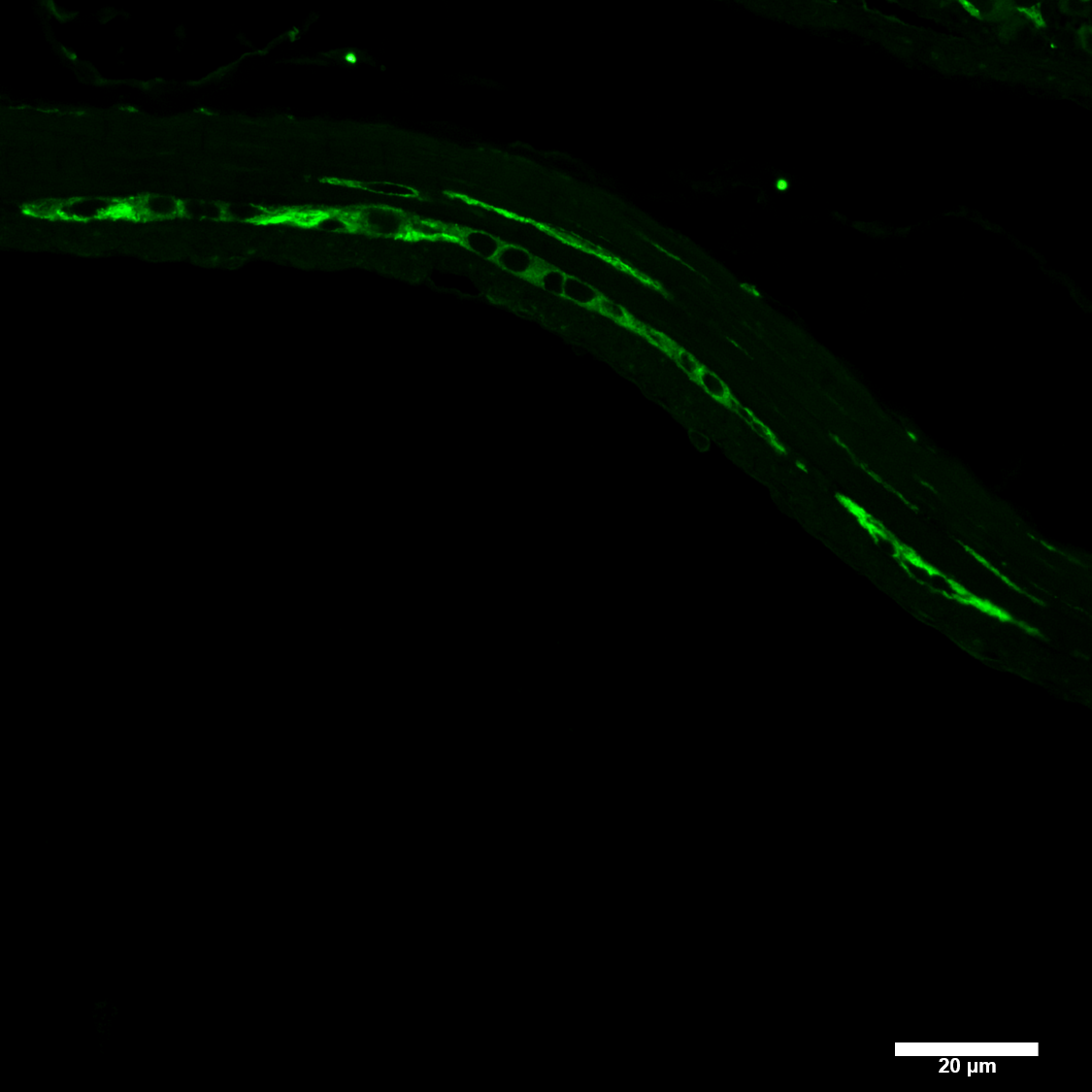

Supplement: Supplementary file 4 [file DataSheet4.zip › Fig 4 original data/Fig 4A/OT+VCR-β III Tubulin.tif]

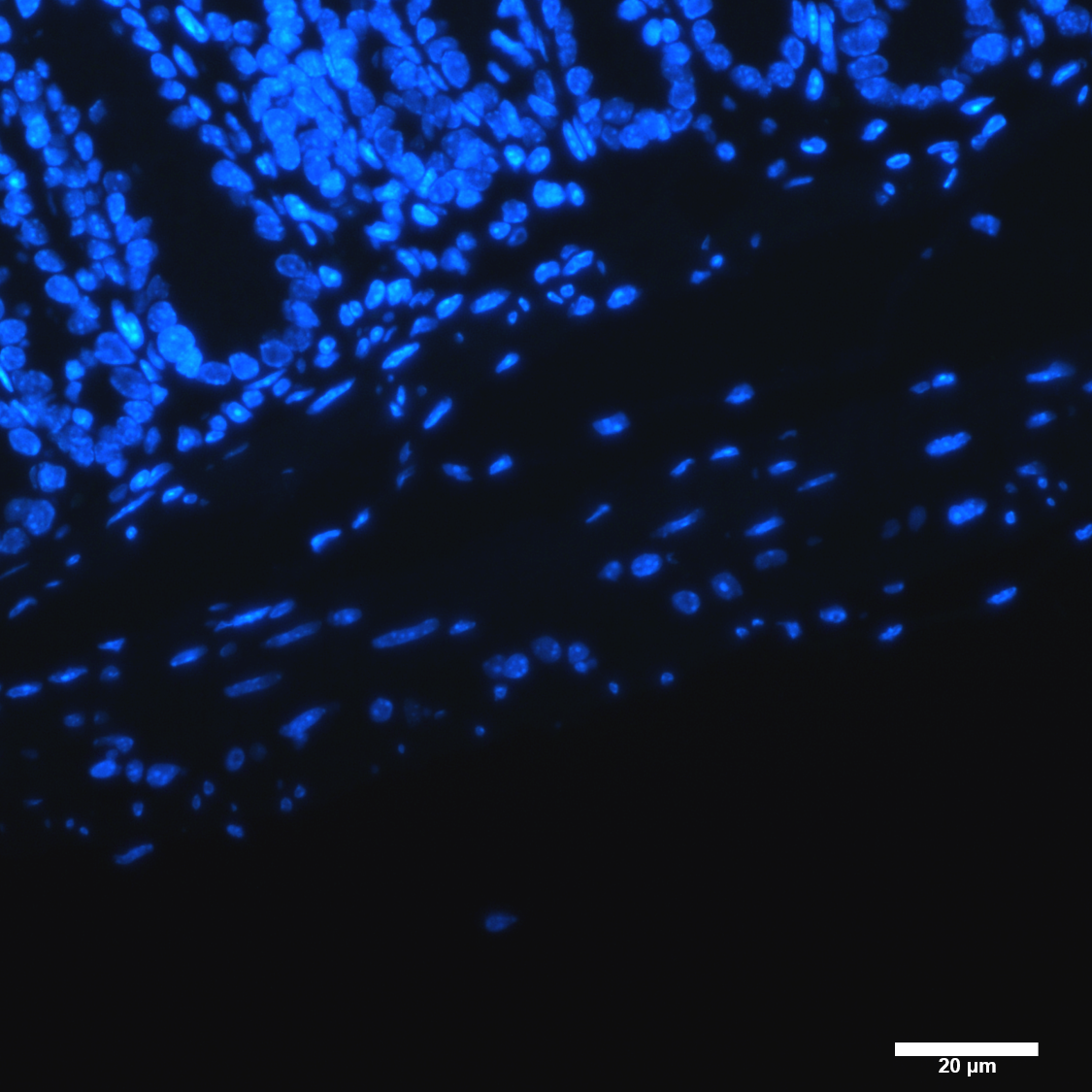

Supplement: Supplementary file 4 [file DataSheet4.zip › Fig 4 original data/Fig 4A/OT-DAPI.tif]

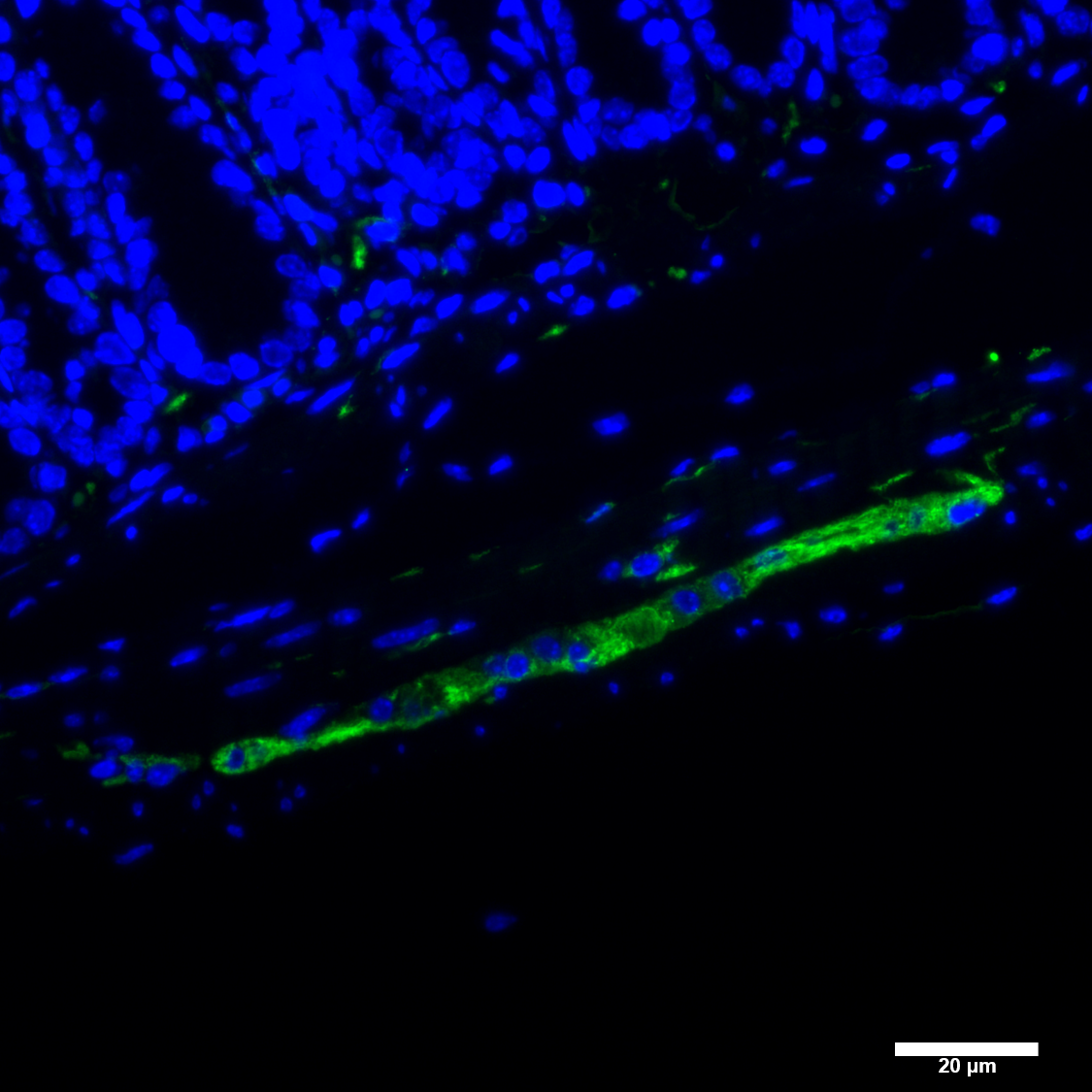

Supplement: Supplementary file 4 [file DataSheet4.zip › Fig 4 original data/Fig 4A/OT-Merge.tif]

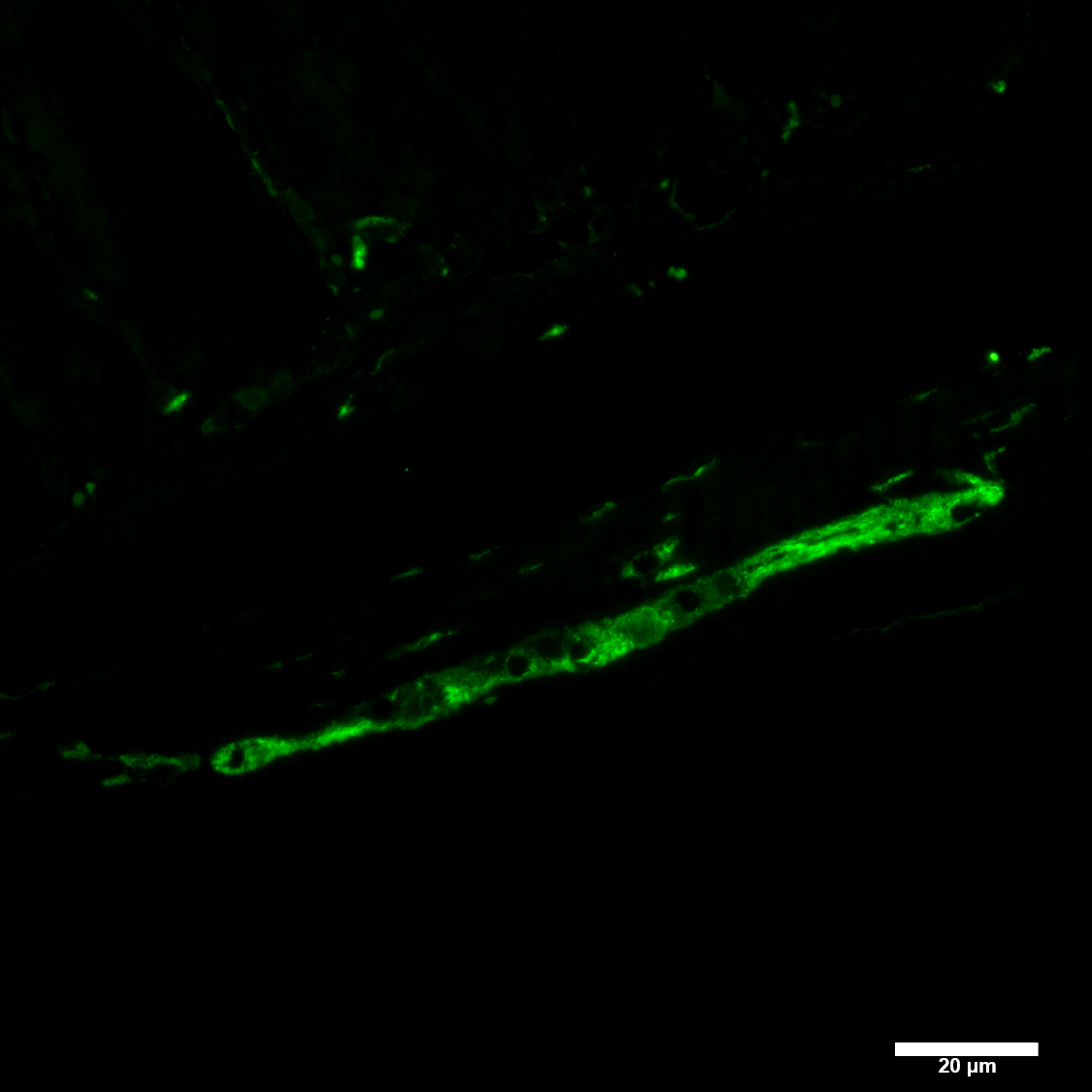

Supplement: Supplementary file 4 [file DataSheet4.zip › Fig 4 original data/Fig 4A/OT-β III Tubulin.tif]

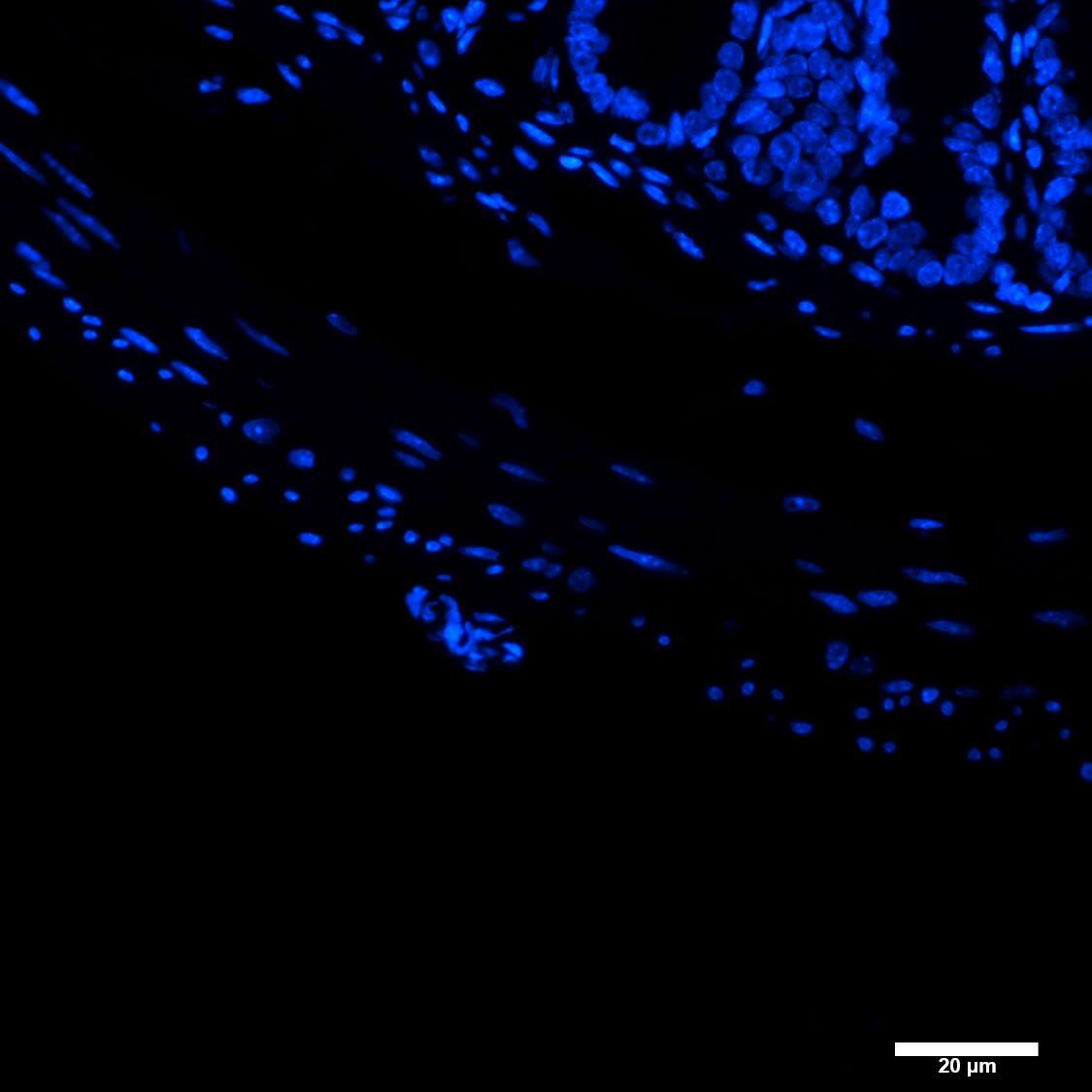

Supplement: Supplementary file 4 [file DataSheet4.zip › Fig 4 original data/Fig 4A/VCR-DAPI.tif]

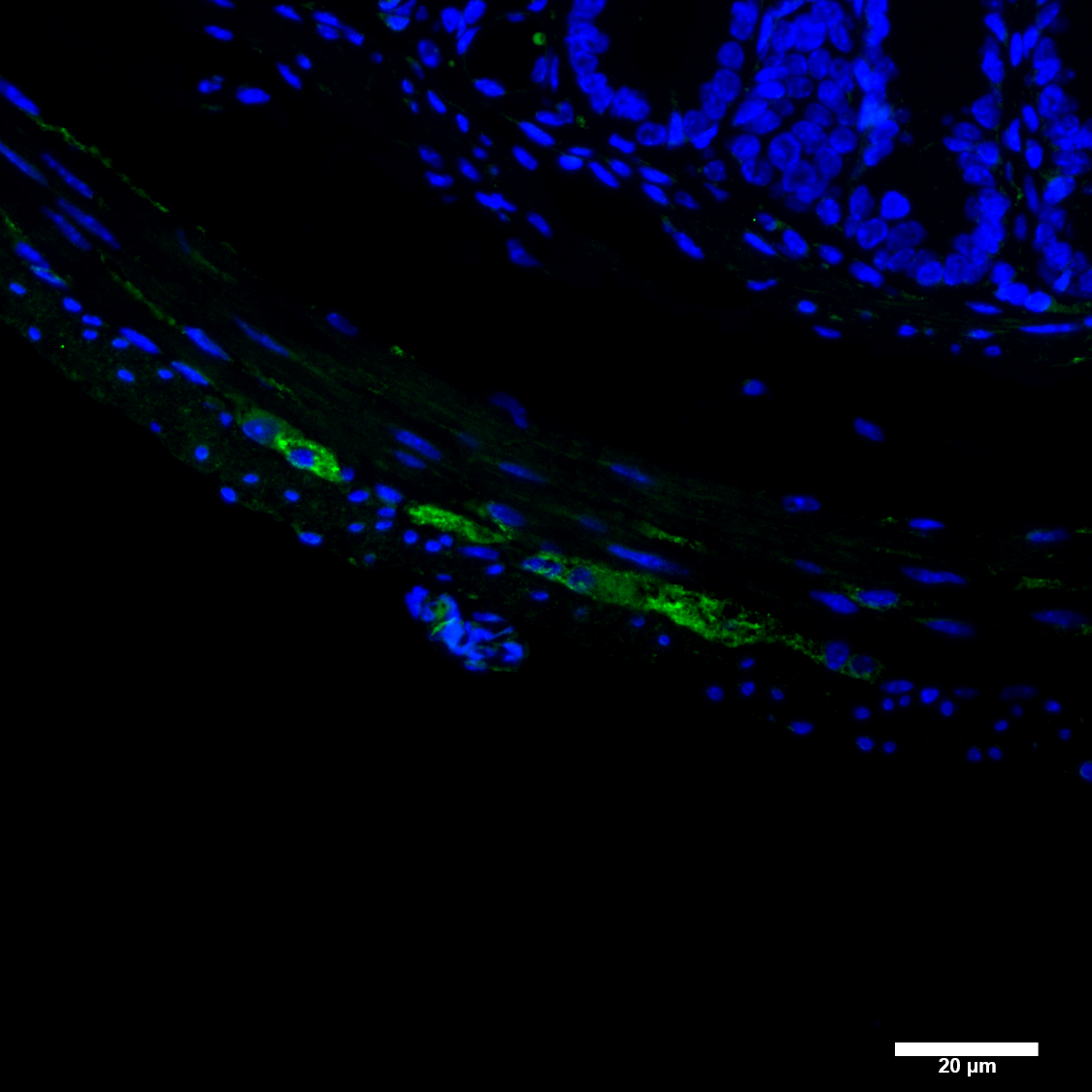

Supplement: Supplementary file 4 [file DataSheet4.zip › Fig 4 original data/Fig 4A/VCR-Merge.tif]

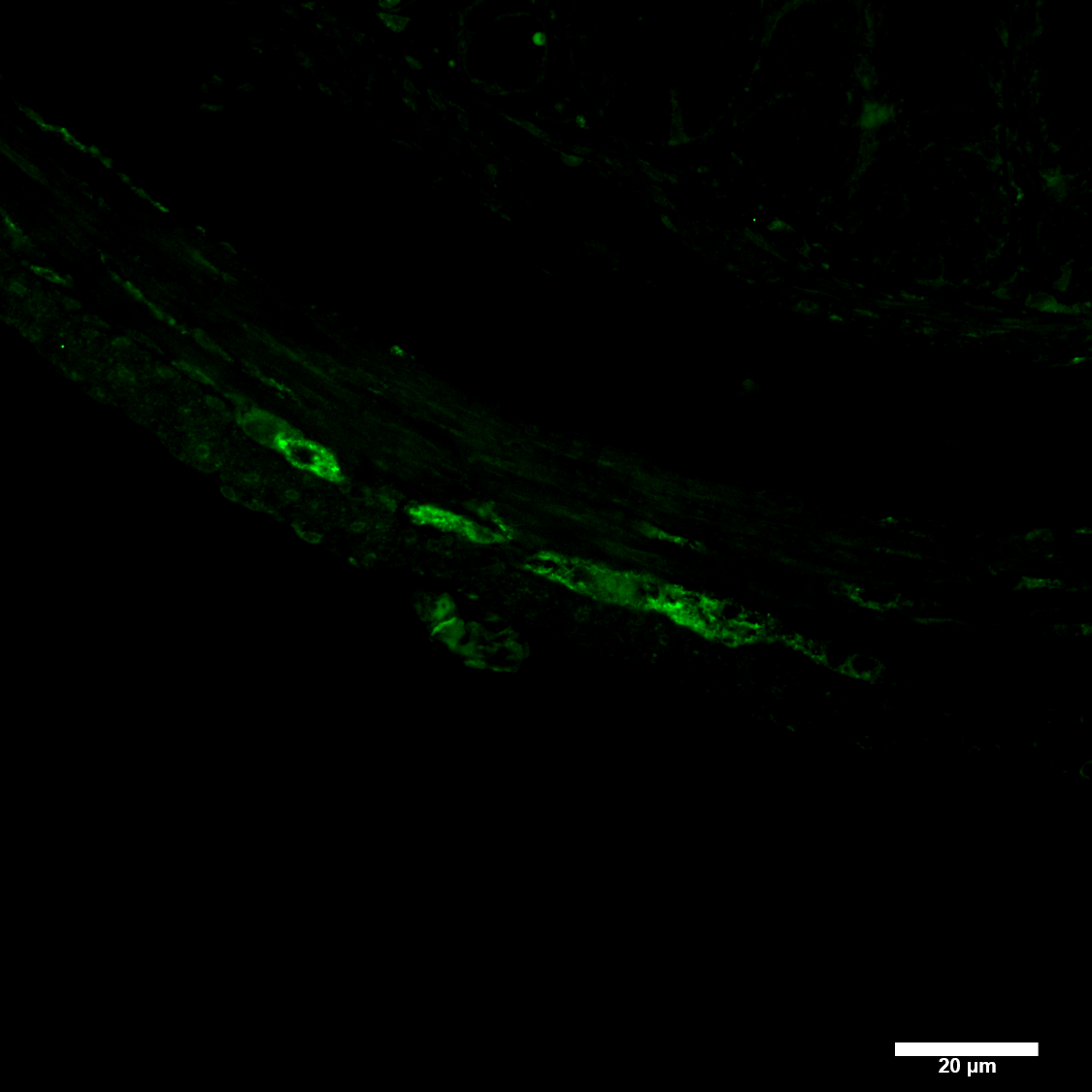

Supplement: Supplementary file 4 [file DataSheet4.zip › Fig 4 original data/Fig 4A/VCR-β III Tubulin.tif]

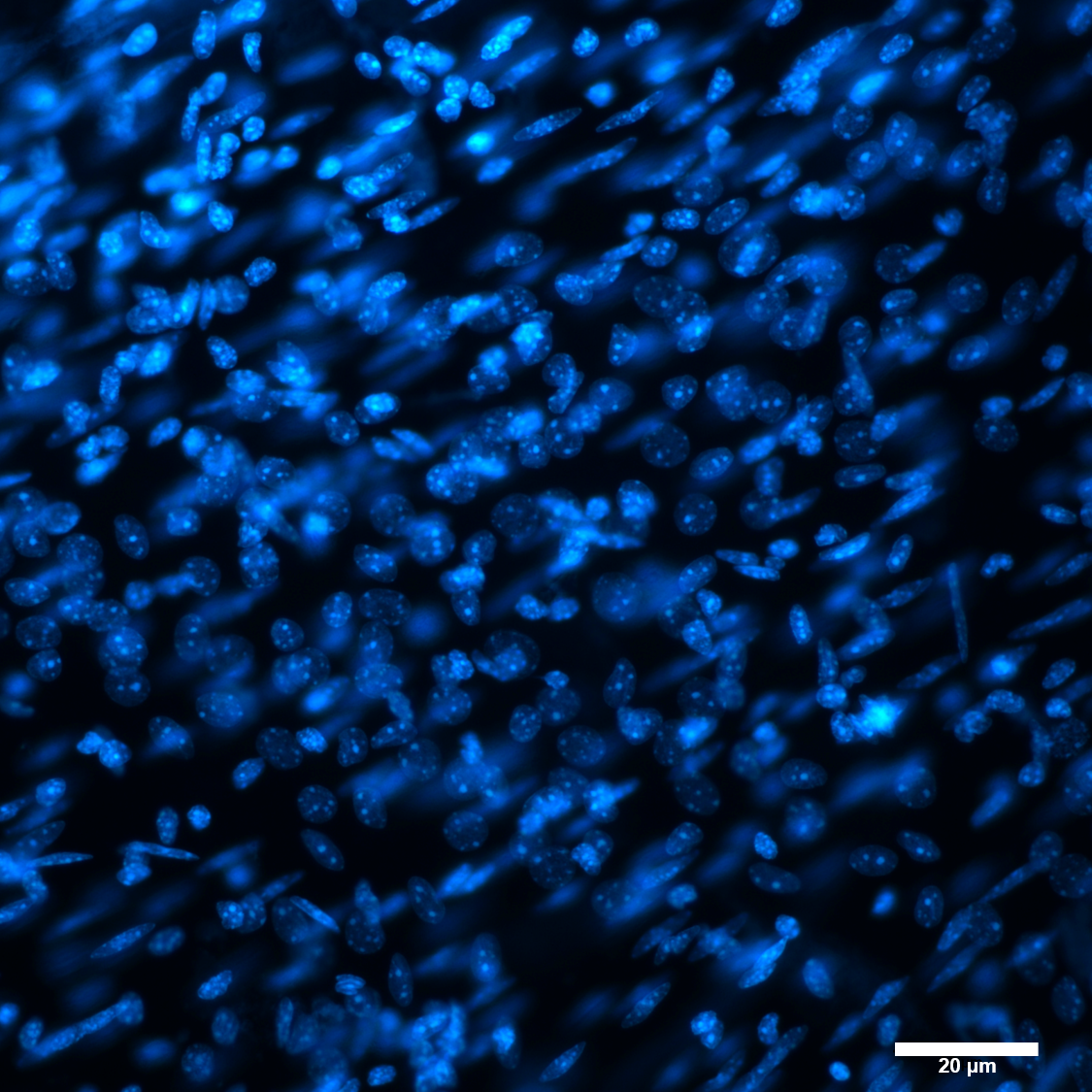

Supplement: Supplementary file 4 [file DataSheet4.zip › Fig 4 original data/Fig 4C/NS-DAPI.tif]

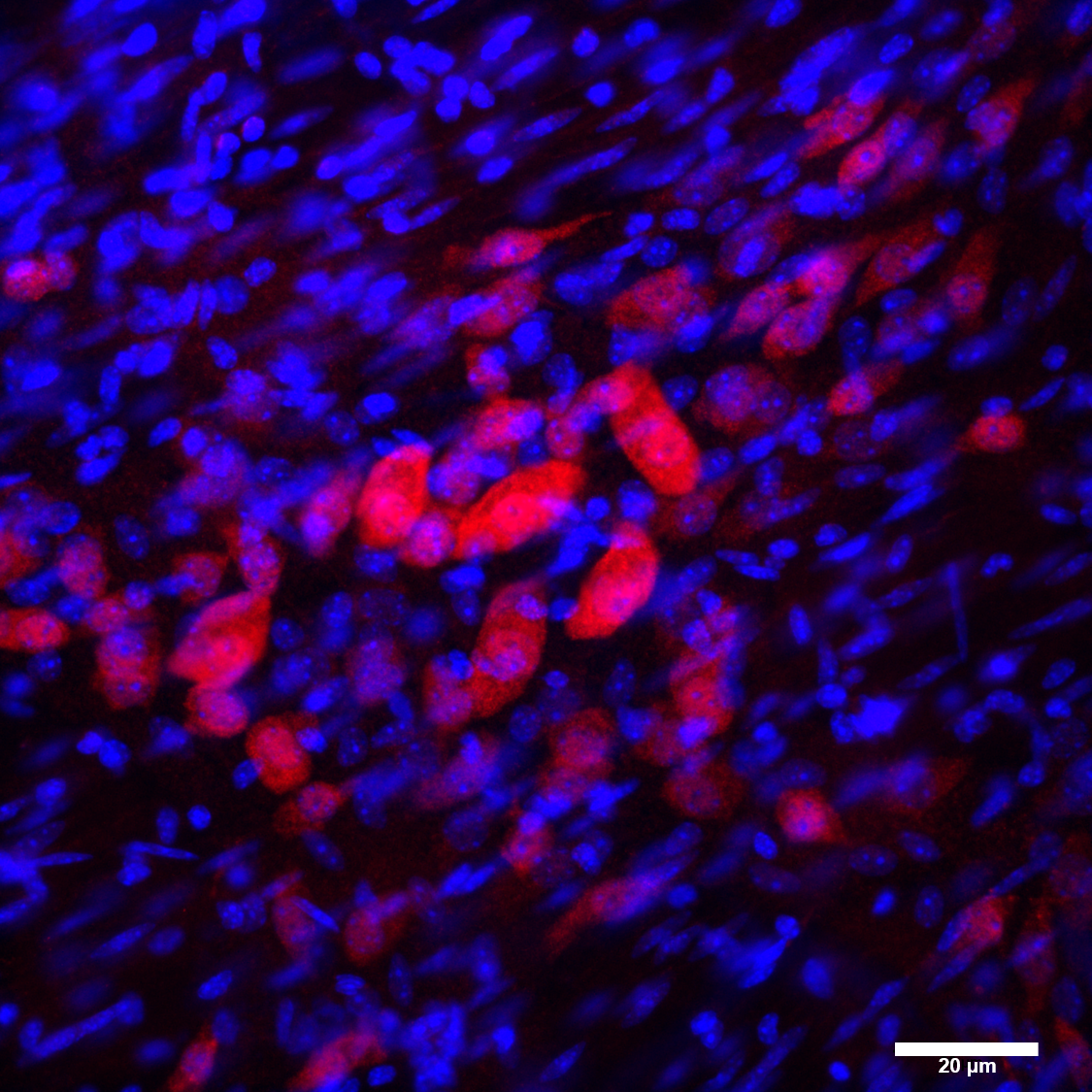

Supplement: Supplementary file 4 [file DataSheet4.zip › Fig 4 original data/Fig 4C/NS-Merge.tif]

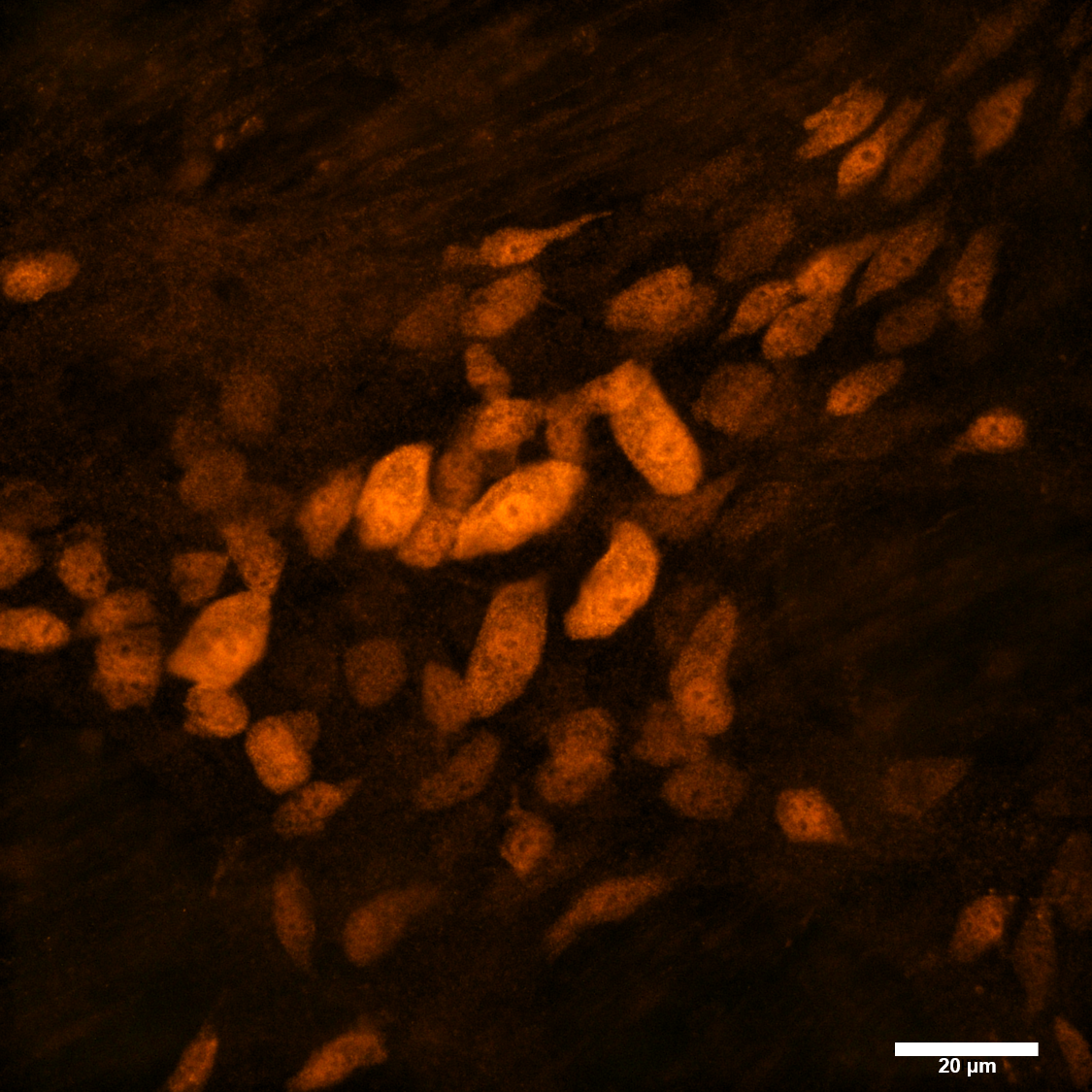

Supplement: Supplementary file 4 [file DataSheet4.zip › Fig 4 original data/Fig 4C/NS-NeuN.tif]

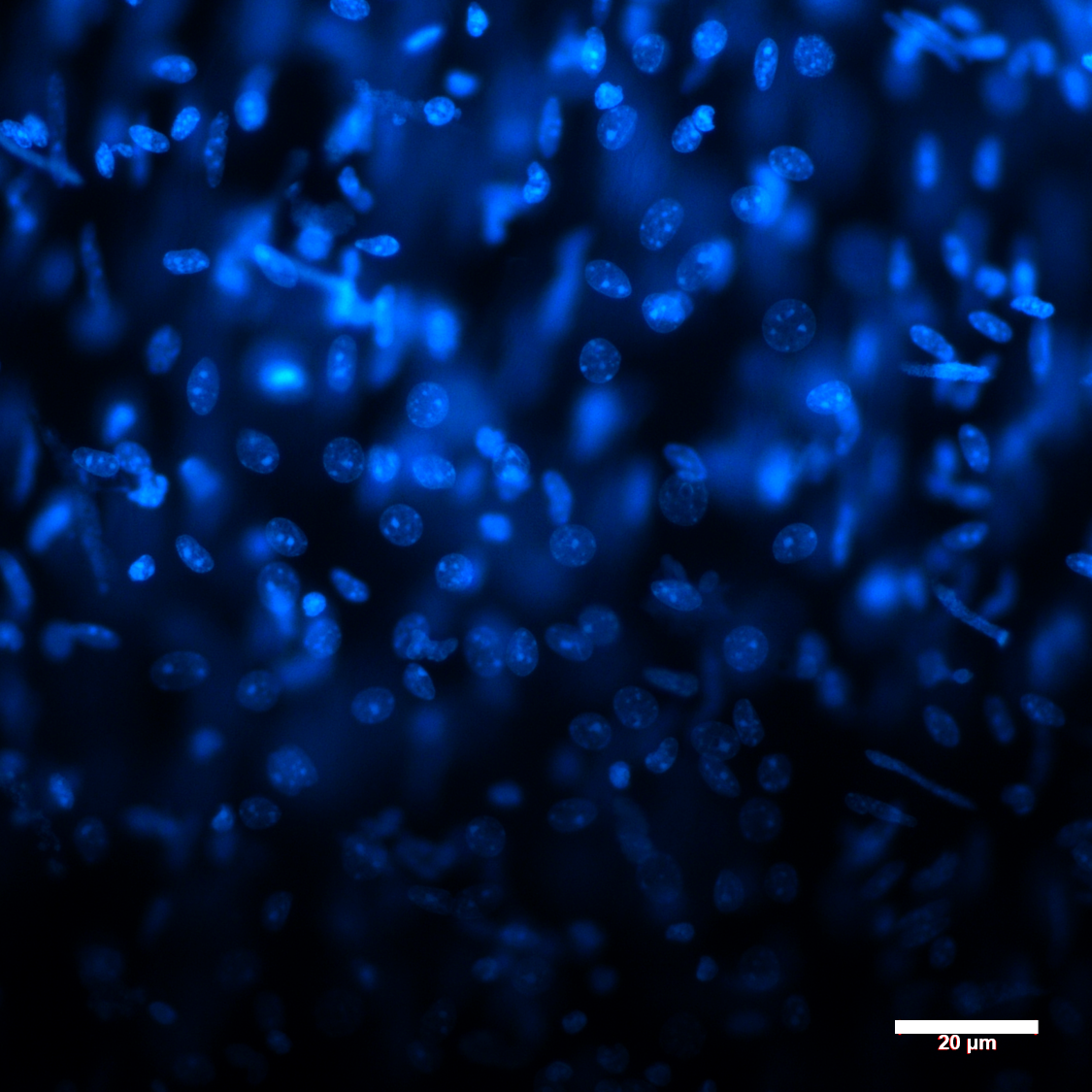

Supplement: Supplementary file 4 [file DataSheet4.zip › Fig 4 original data/Fig 4C/OT+VCR-DAPI.tif]

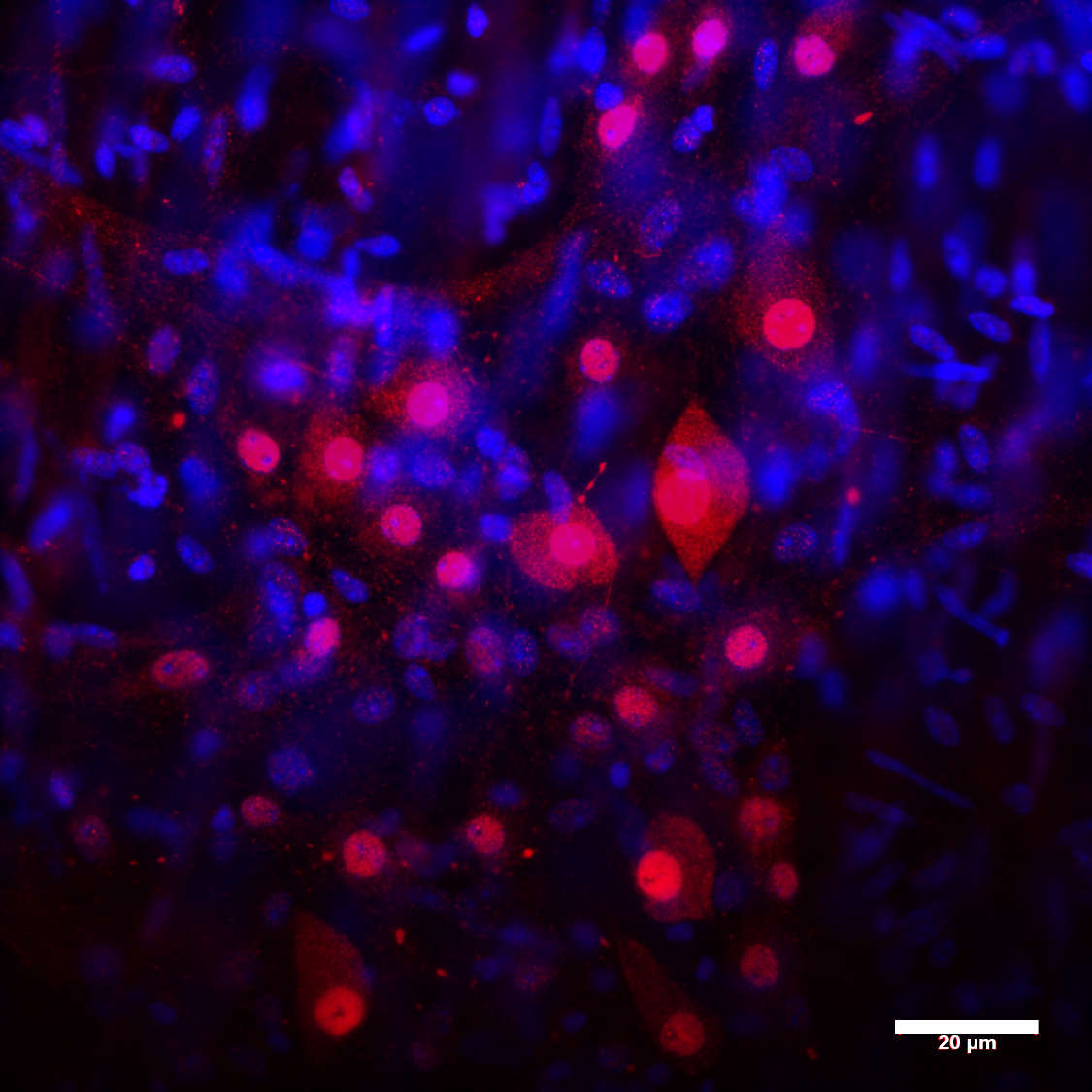

Supplement: Supplementary file 4 [file DataSheet4.zip › Fig 4 original data/Fig 4C/OT+VCR-Merge.tif]

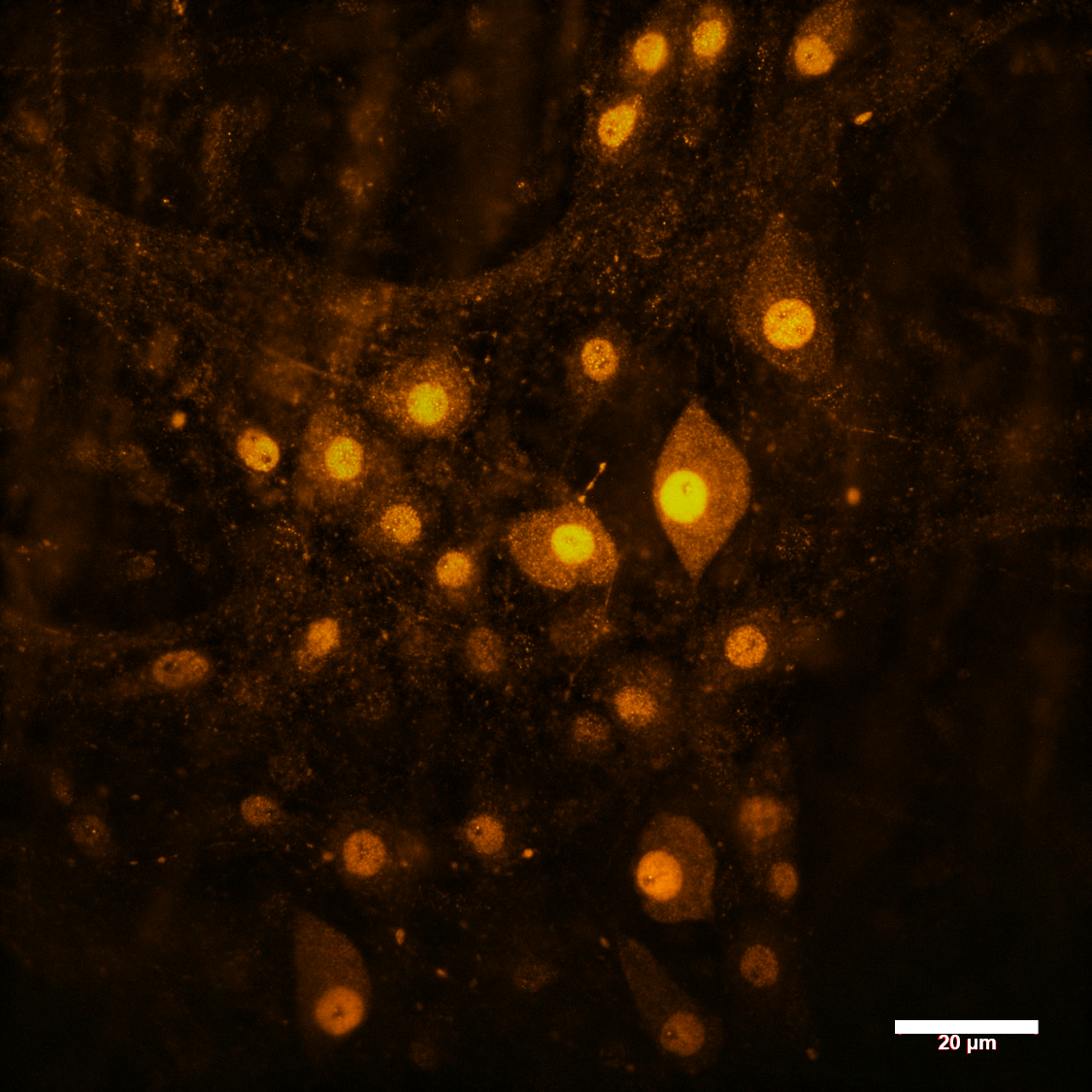

Supplement: Supplementary file 4 [file DataSheet4.zip › Fig 4 original data/Fig 4C/OT+VCR-NeuN.tif]

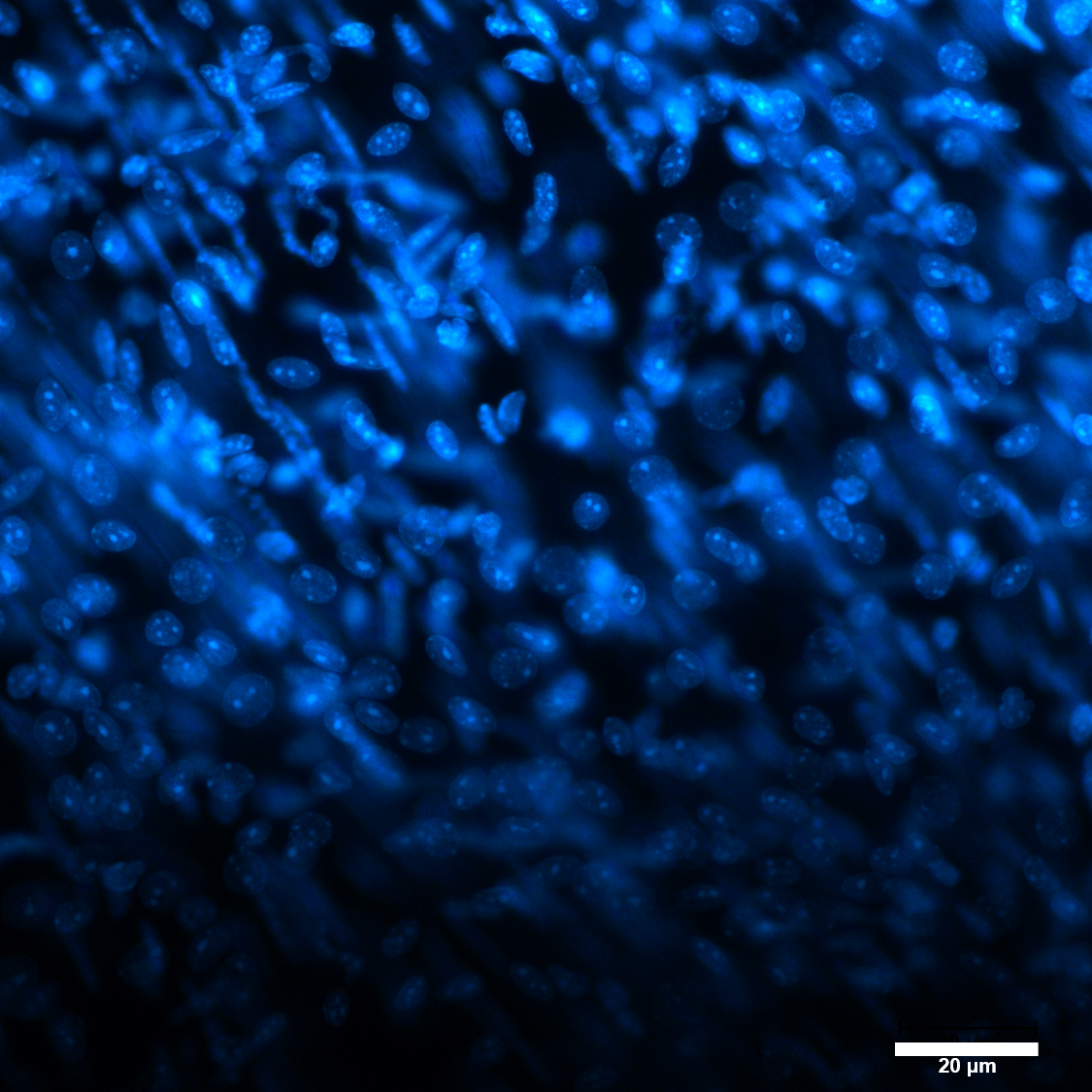

Supplement: Supplementary file 4 [file DataSheet4.zip › Fig 4 original data/Fig 4C/OT-DAPI.tif]

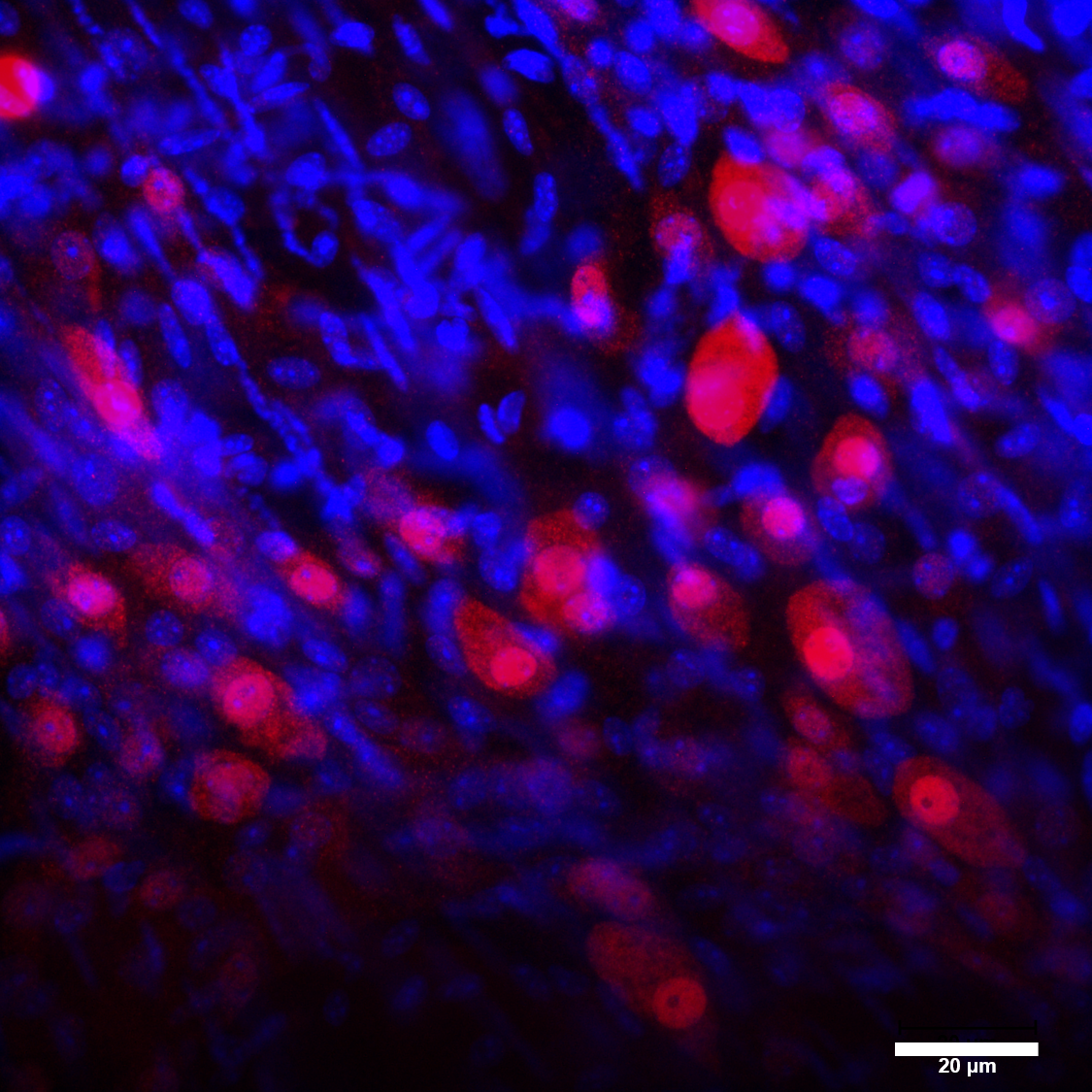

Supplement: Supplementary file 4 [file DataSheet4.zip › Fig 4 original data/Fig 4C/OT-Merge.tif]

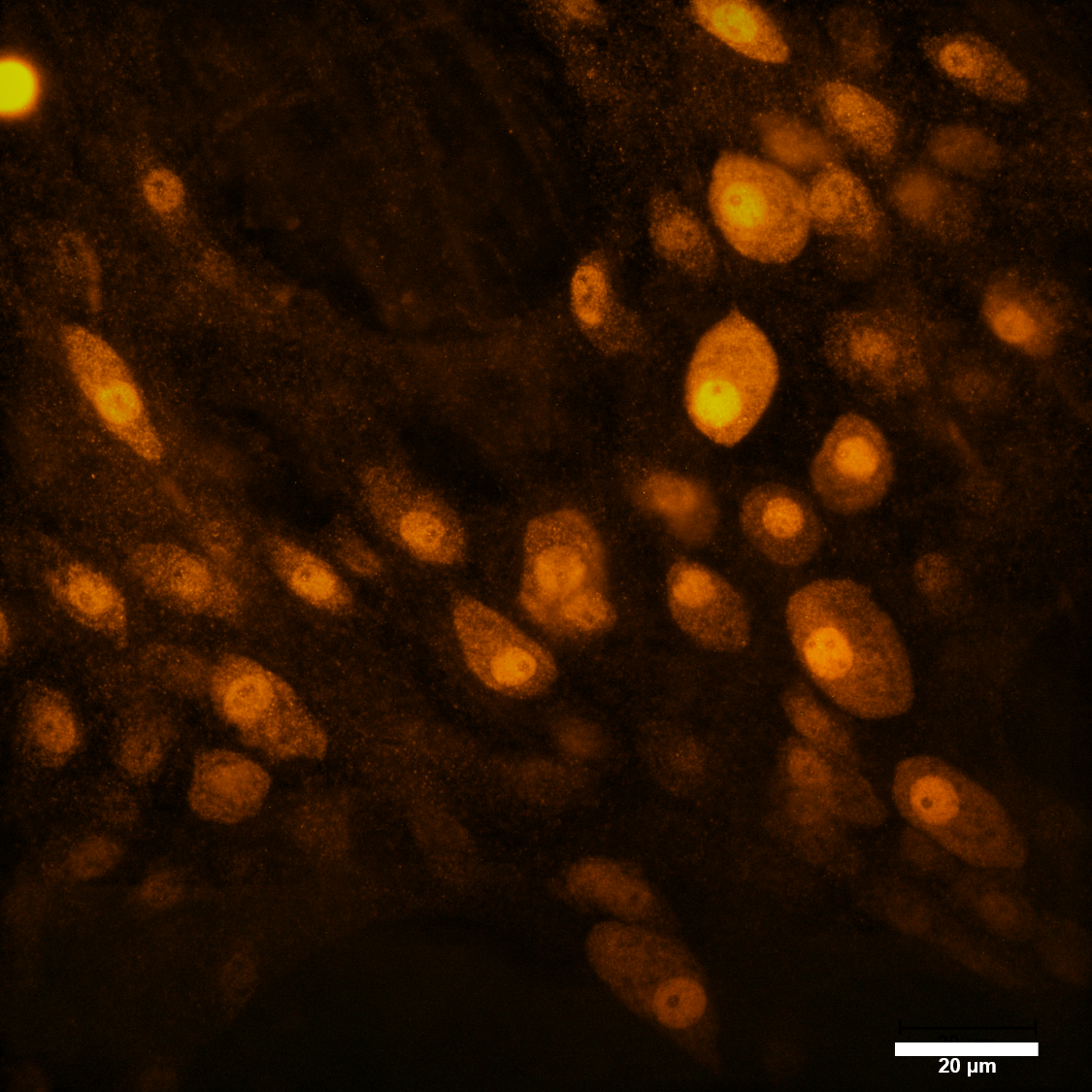

Supplement: Supplementary file 4 [file DataSheet4.zip › Fig 4 original data/Fig 4C/OT-NeuN.tif]

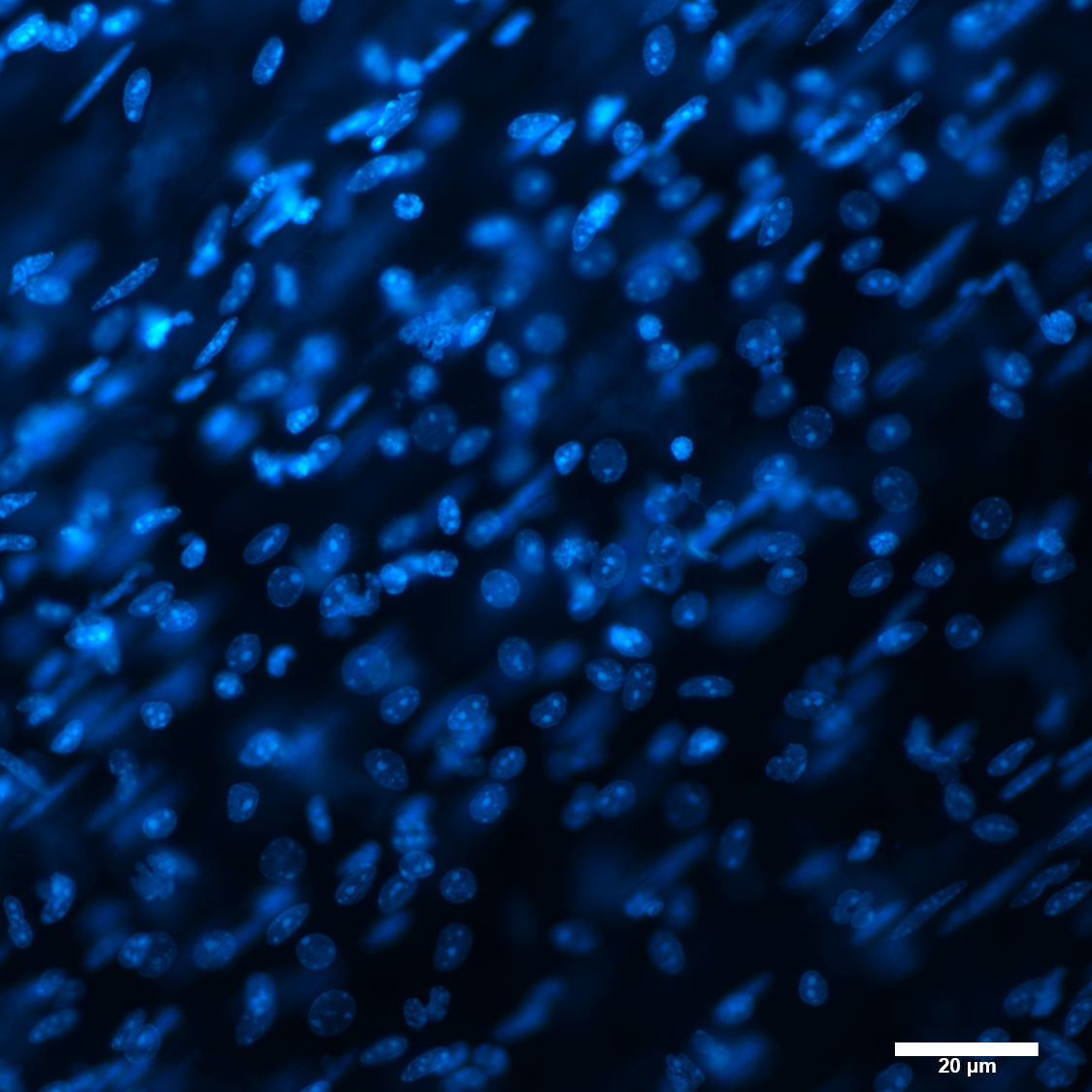

Supplement: Supplementary file 4 [file DataSheet4.zip › Fig 4 original data/Fig 4C/VCR-DAPI.tif]

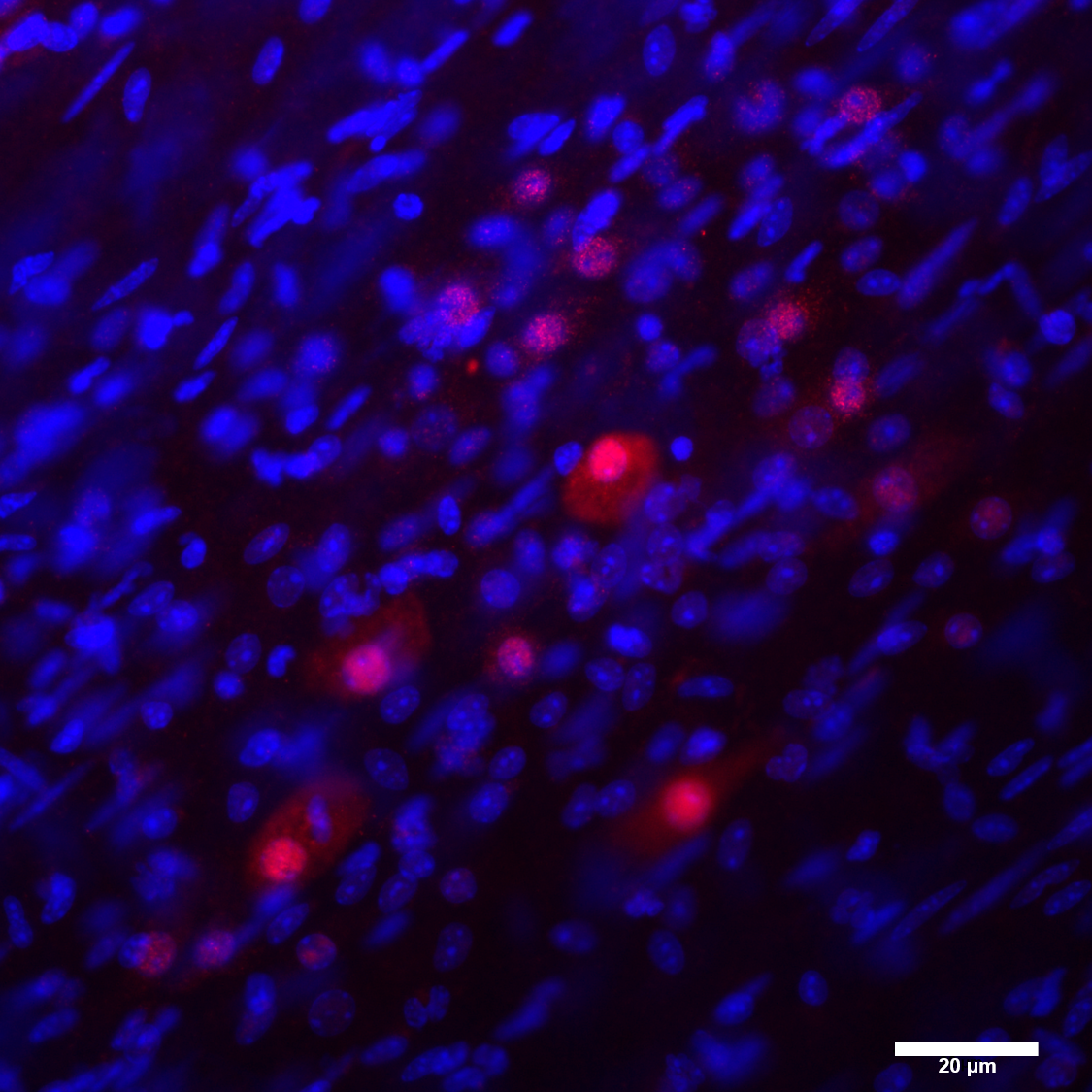

Supplement: Supplementary file 4 [file DataSheet4.zip › Fig 4 original data/Fig 4C/VCR-Merge.tif]

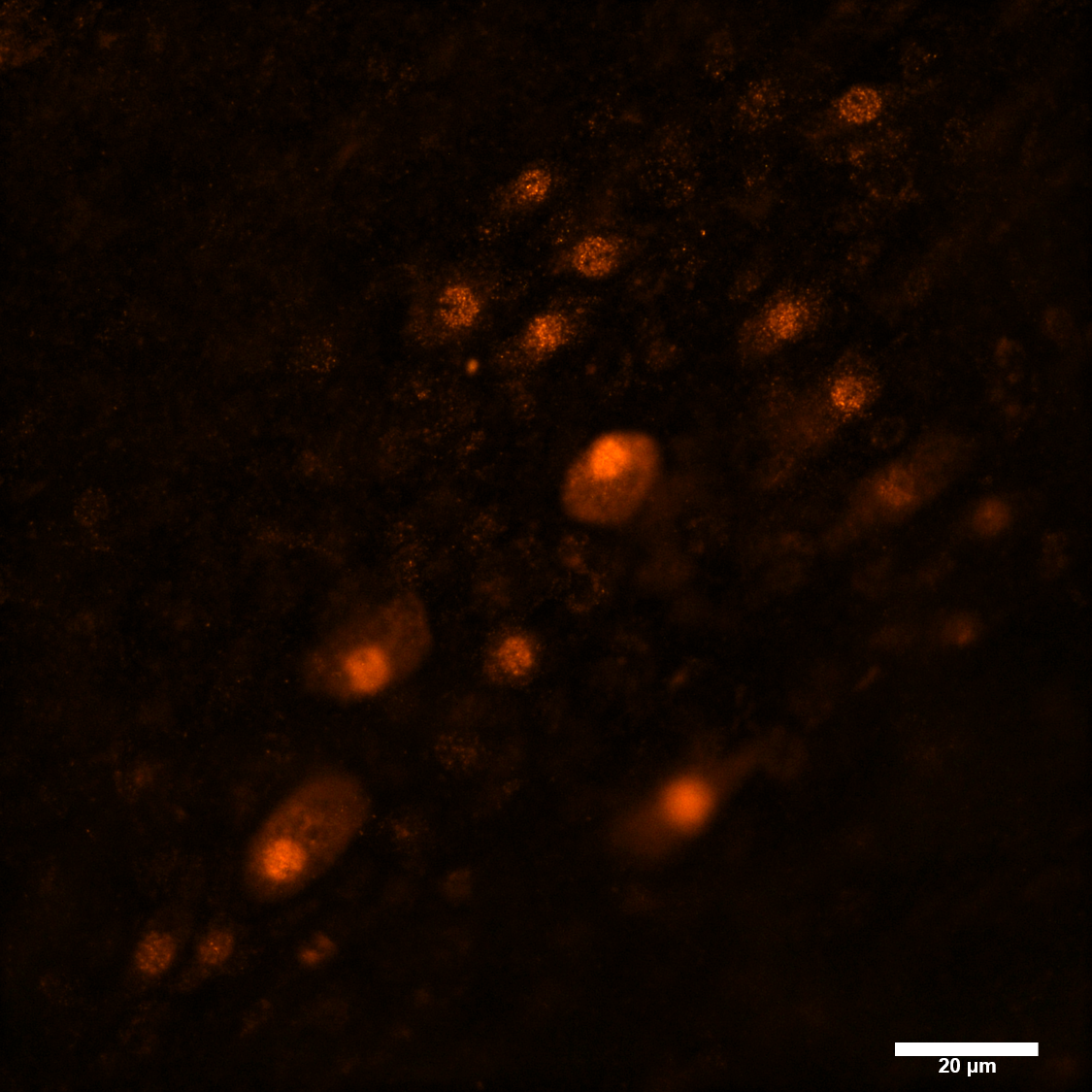

Supplement: Supplementary file 4 [file DataSheet4.zip › Fig 4 original data/Fig 4C/VCR-NeuN.tif]

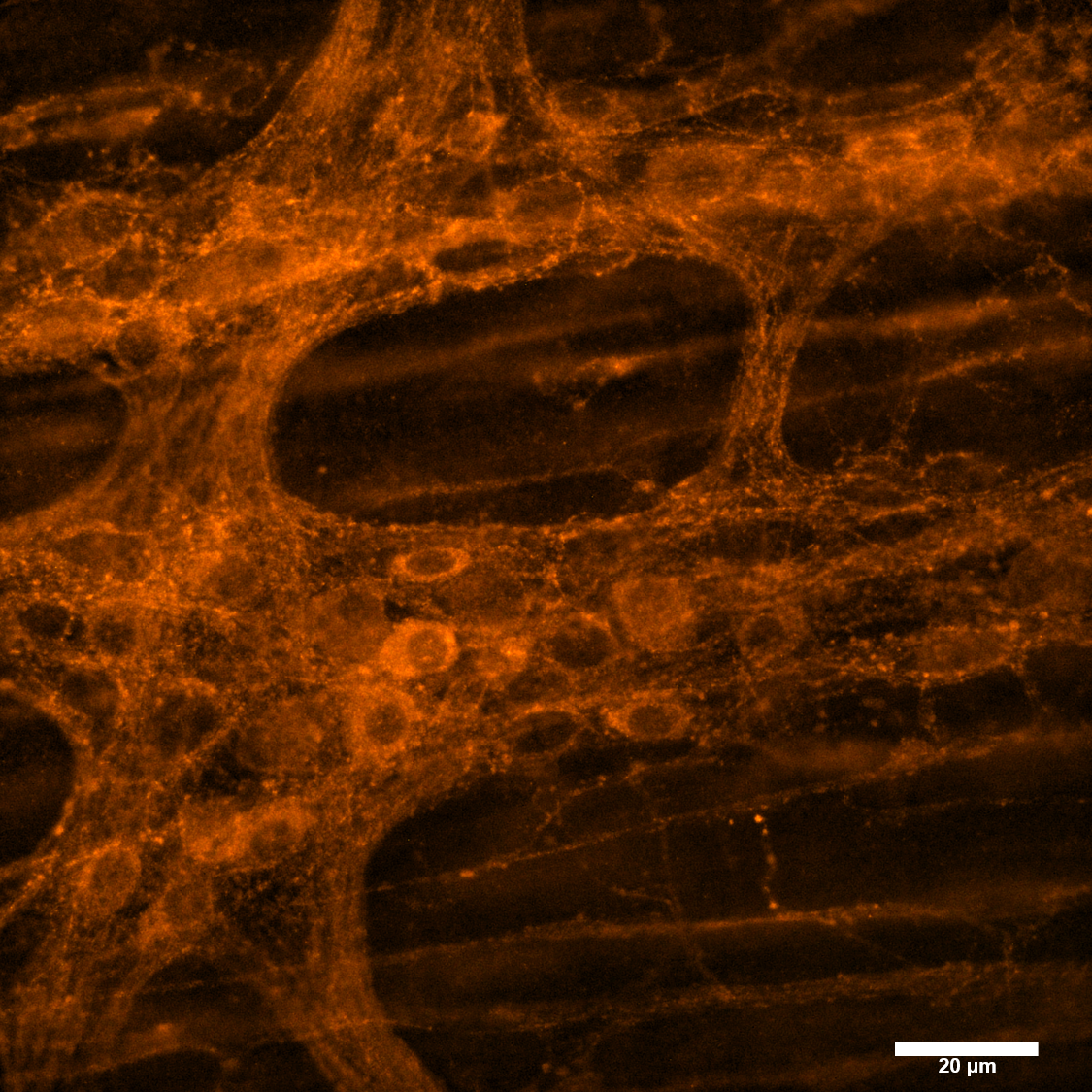

Supplement: Supplementary file 6 [file DataSheet6.zip › Fig 6 original data/Fig 6A/NS-ChAT.tif]

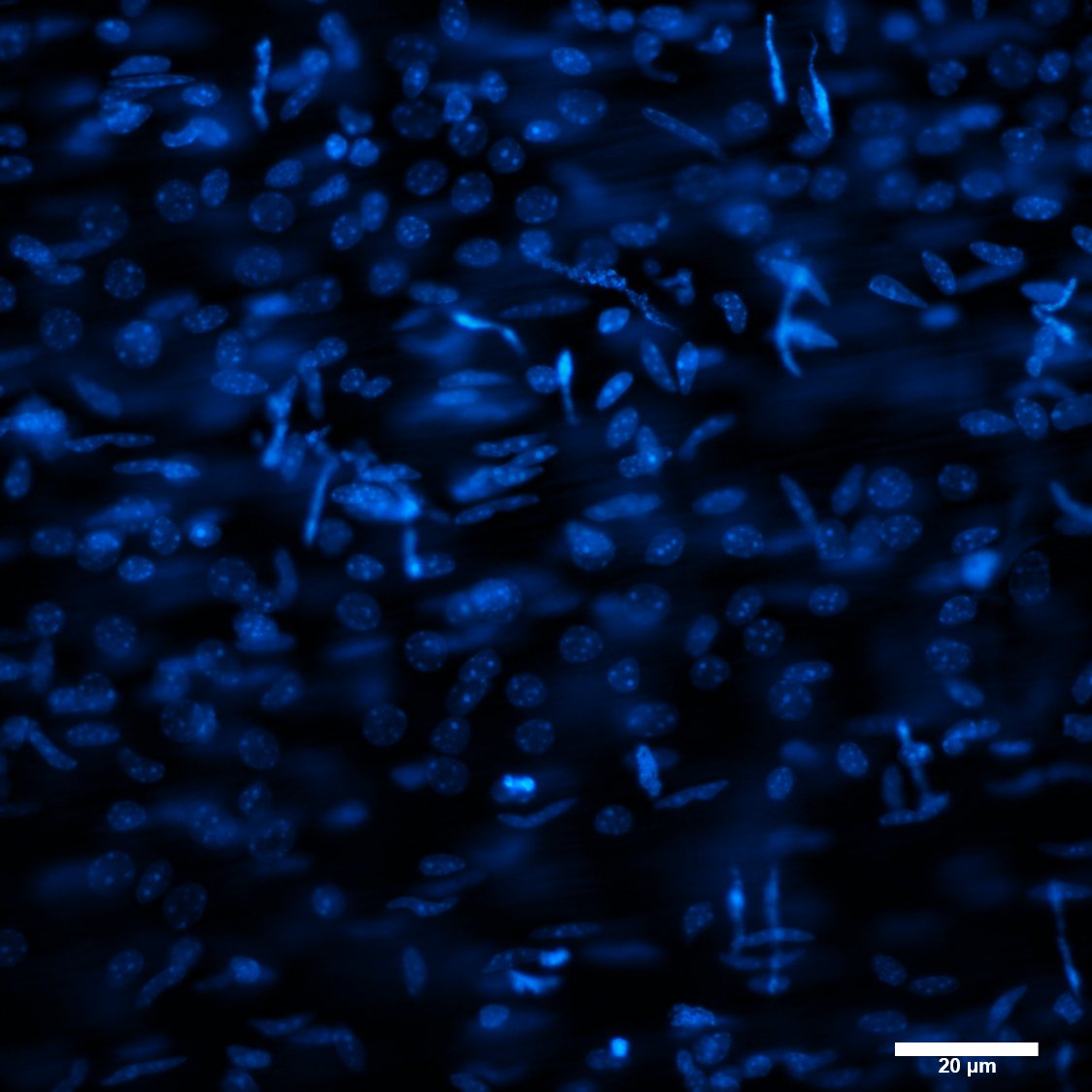

Supplement: Supplementary file 6 [file DataSheet6.zip › Fig 6 original data/Fig 6A/NS-DAPI.tif]

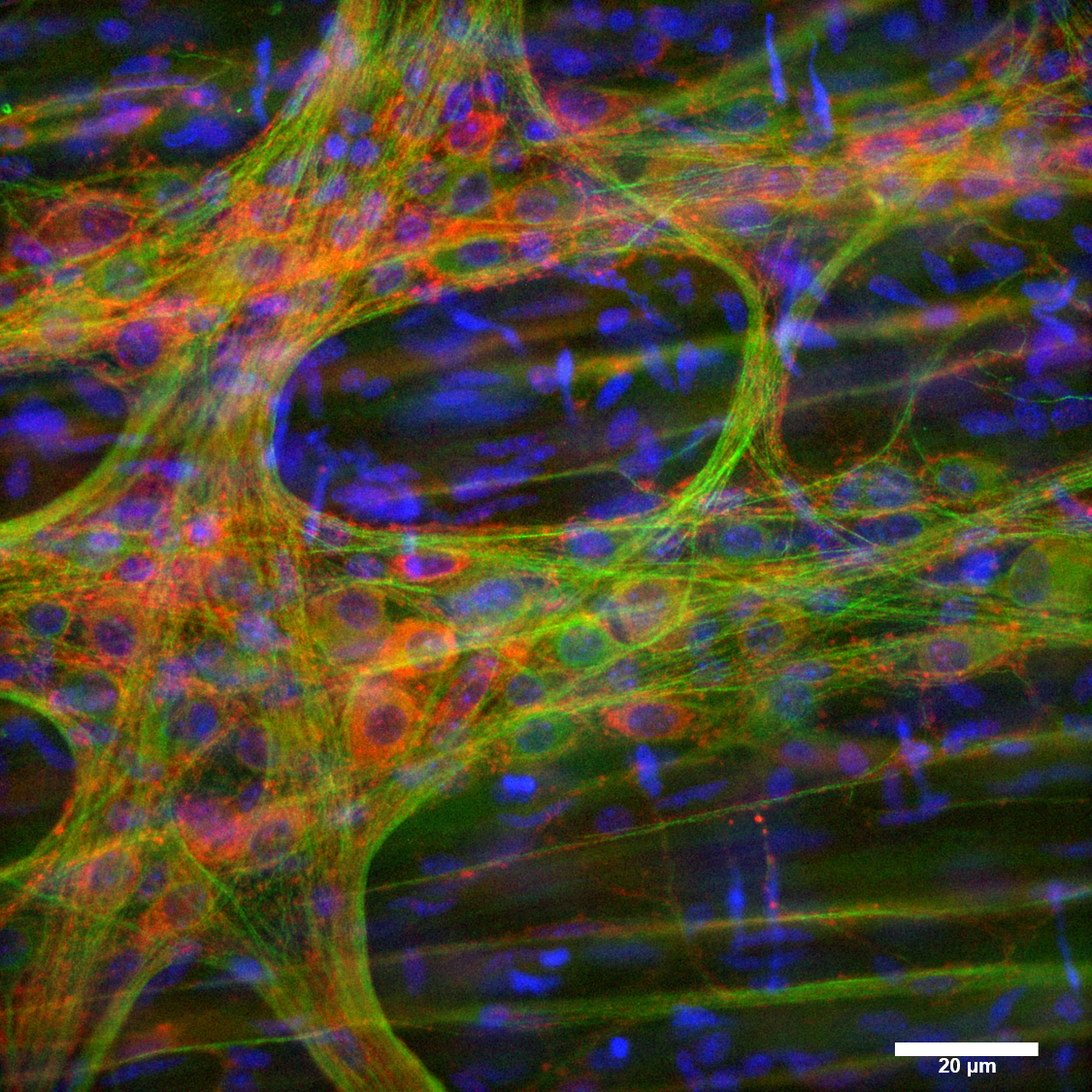

Supplement: Supplementary file 6 [file DataSheet6.zip › Fig 6 original data/Fig 6A/NS-Merge.tif]

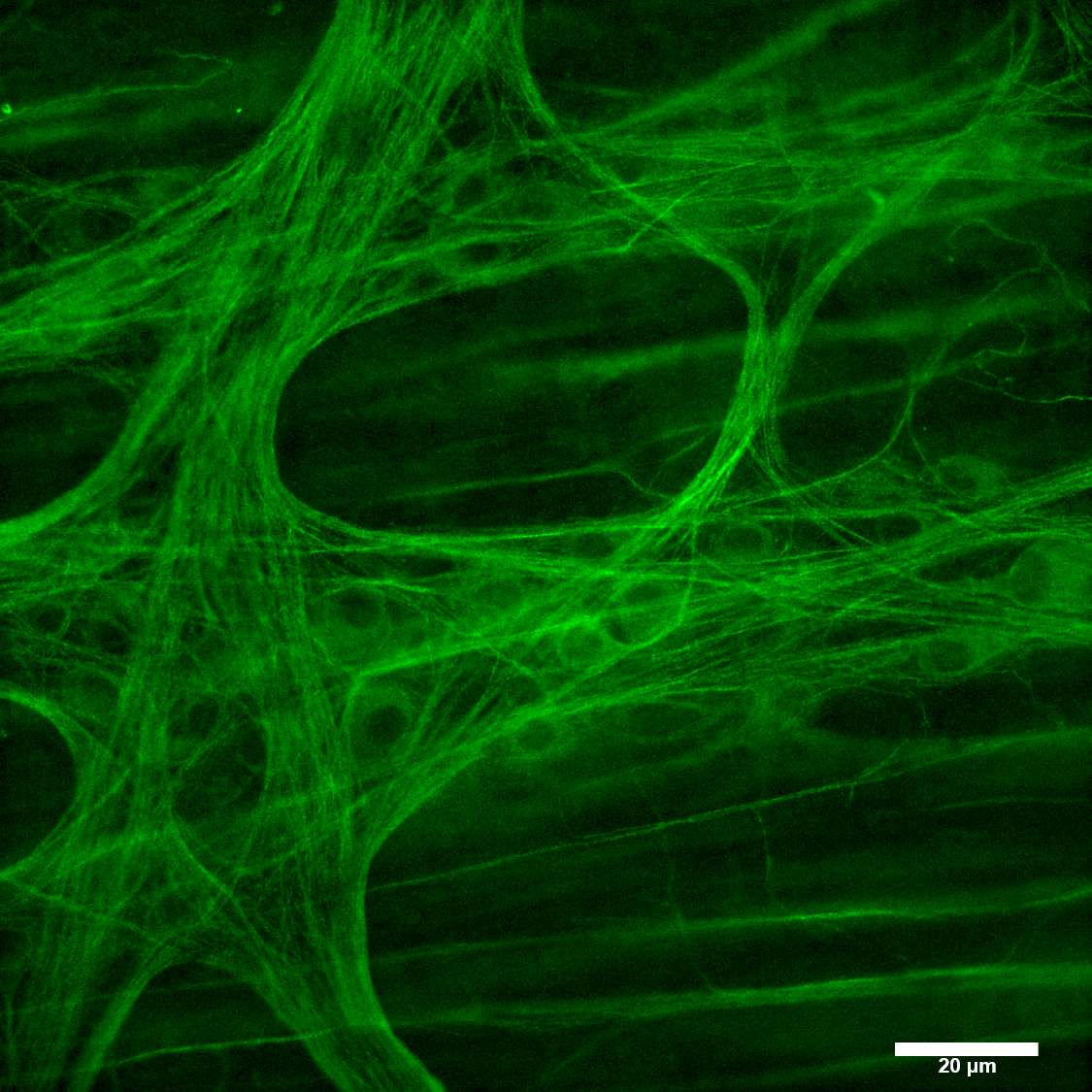

Supplement: Supplementary file 6 [file DataSheet6.zip › Fig 6 original data/Fig 6A/NS-β III Tubulin.tif]

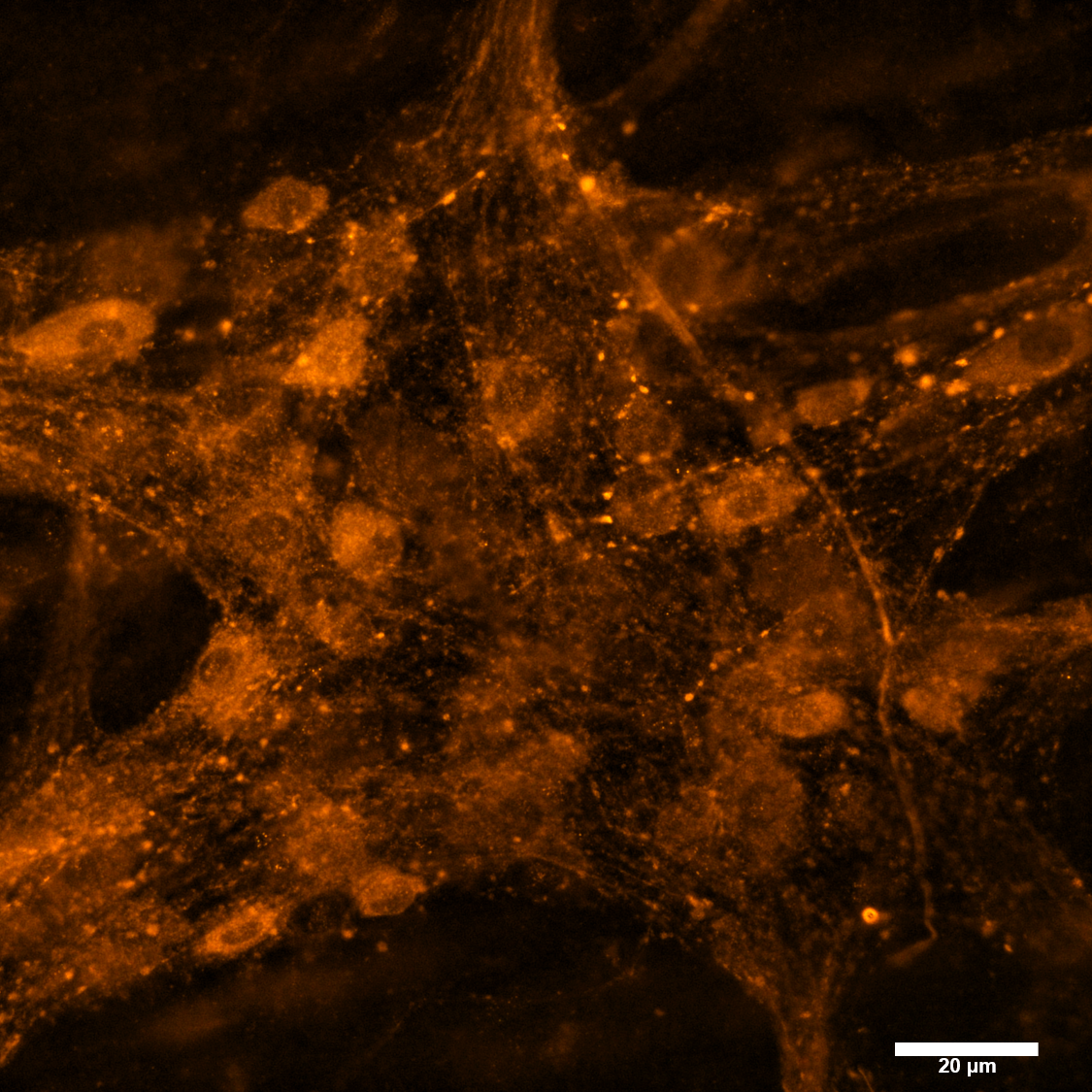

Supplement: Supplementary file 6 [file DataSheet6.zip › Fig 6 original data/Fig 6A/OT+VCR-ChAT.tif]

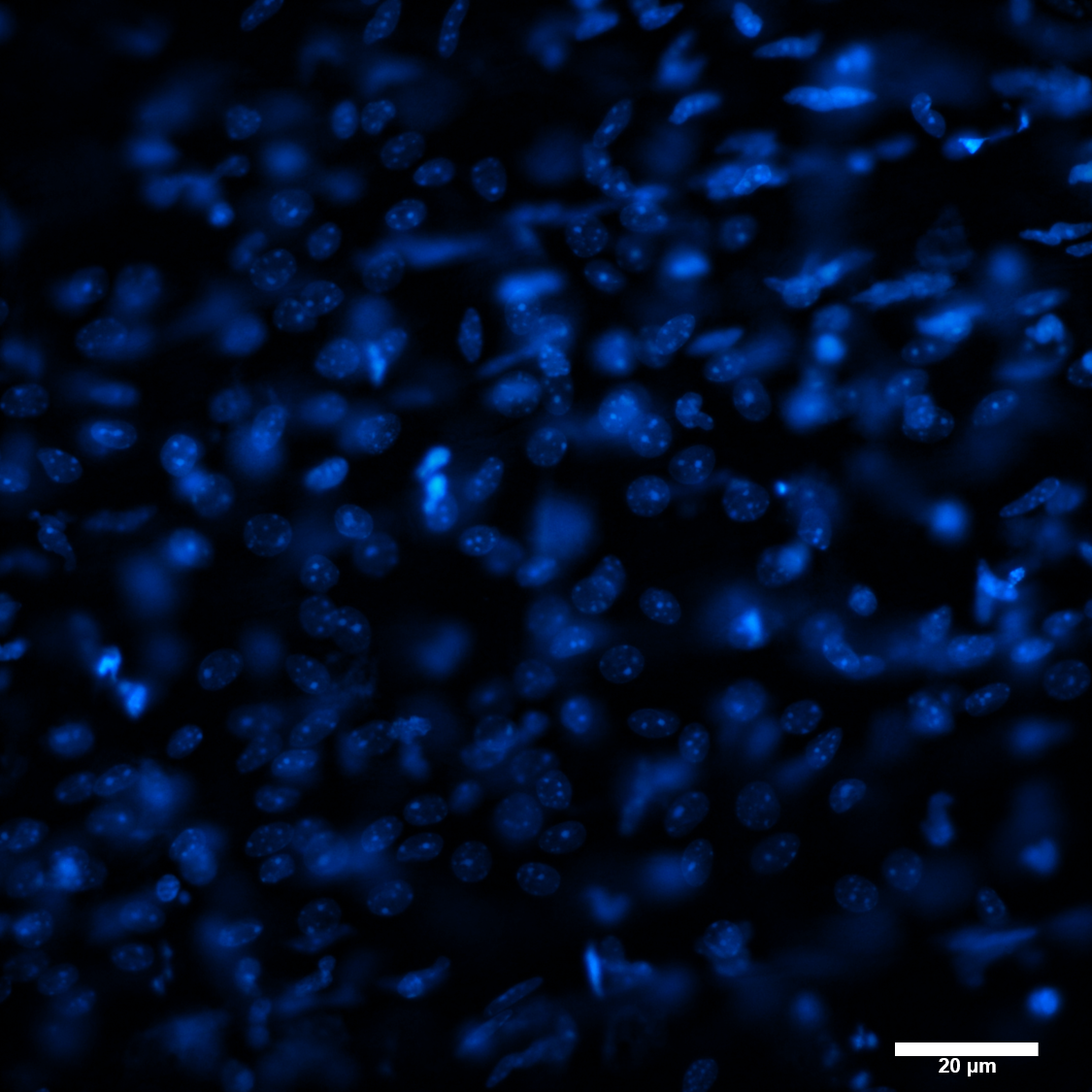

Supplement: Supplementary file 6 [file DataSheet6.zip › Fig 6 original data/Fig 6A/OT+VCR-DAPI.tif]

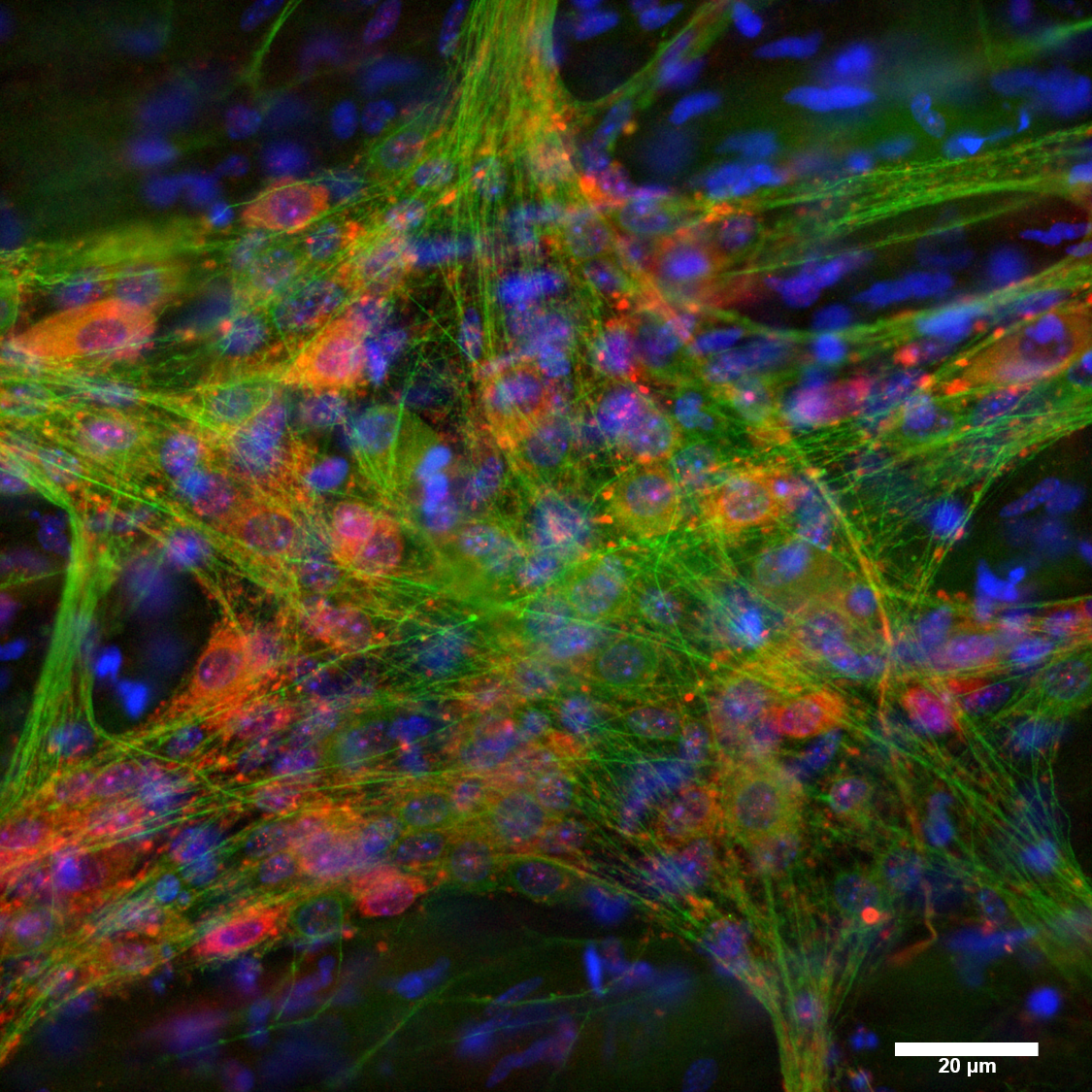

Supplement: Supplementary file 6 [file DataSheet6.zip › Fig 6 original data/Fig 6A/OT+VCR-Merge.tif]

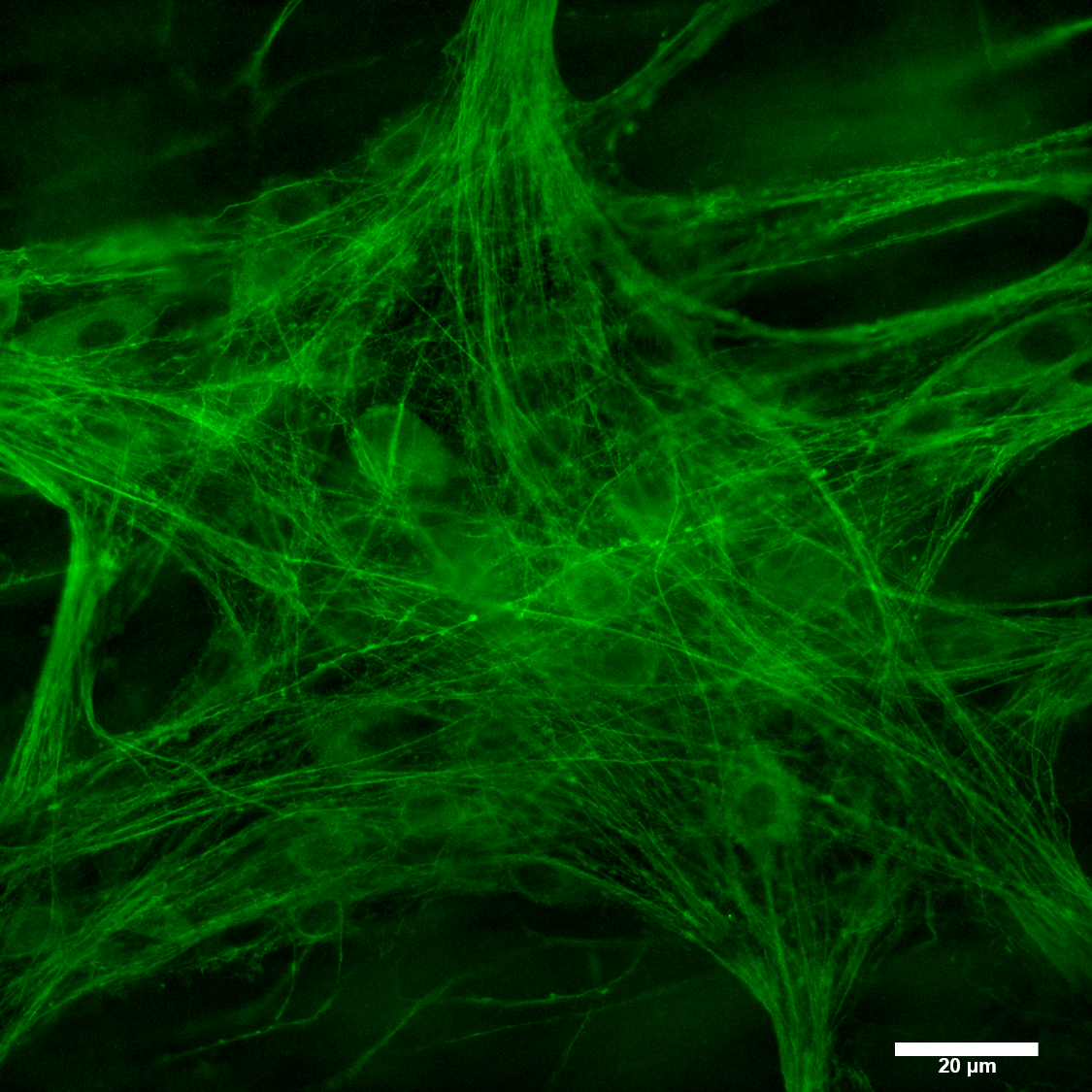

Supplement: Supplementary file 6 [file DataSheet6.zip › Fig 6 original data/Fig 6A/OT+VCR-β III Tubulin.tif]

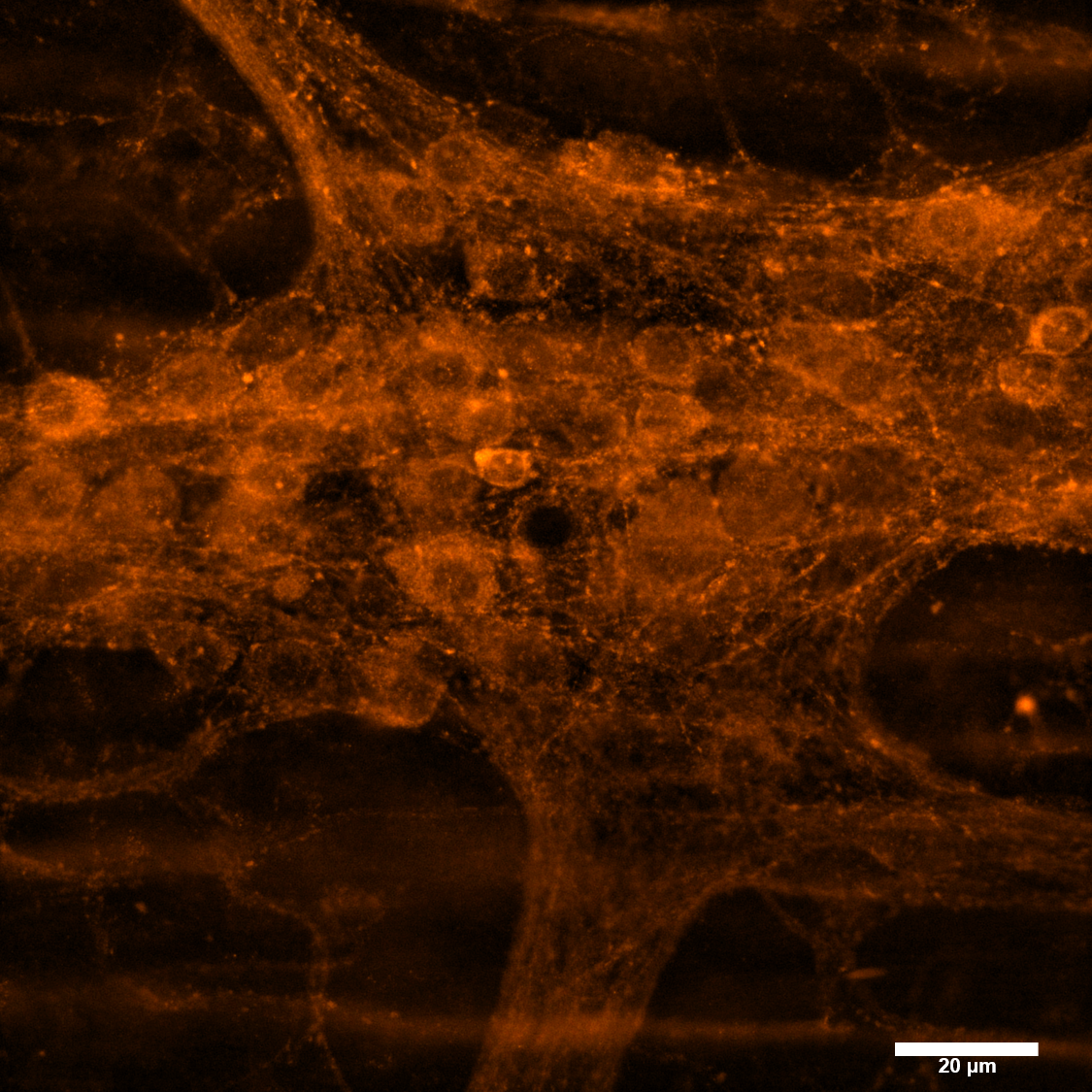

Supplement: Supplementary file 6 [file DataSheet6.zip › Fig 6 original data/Fig 6A/OT-ChAT.tif]

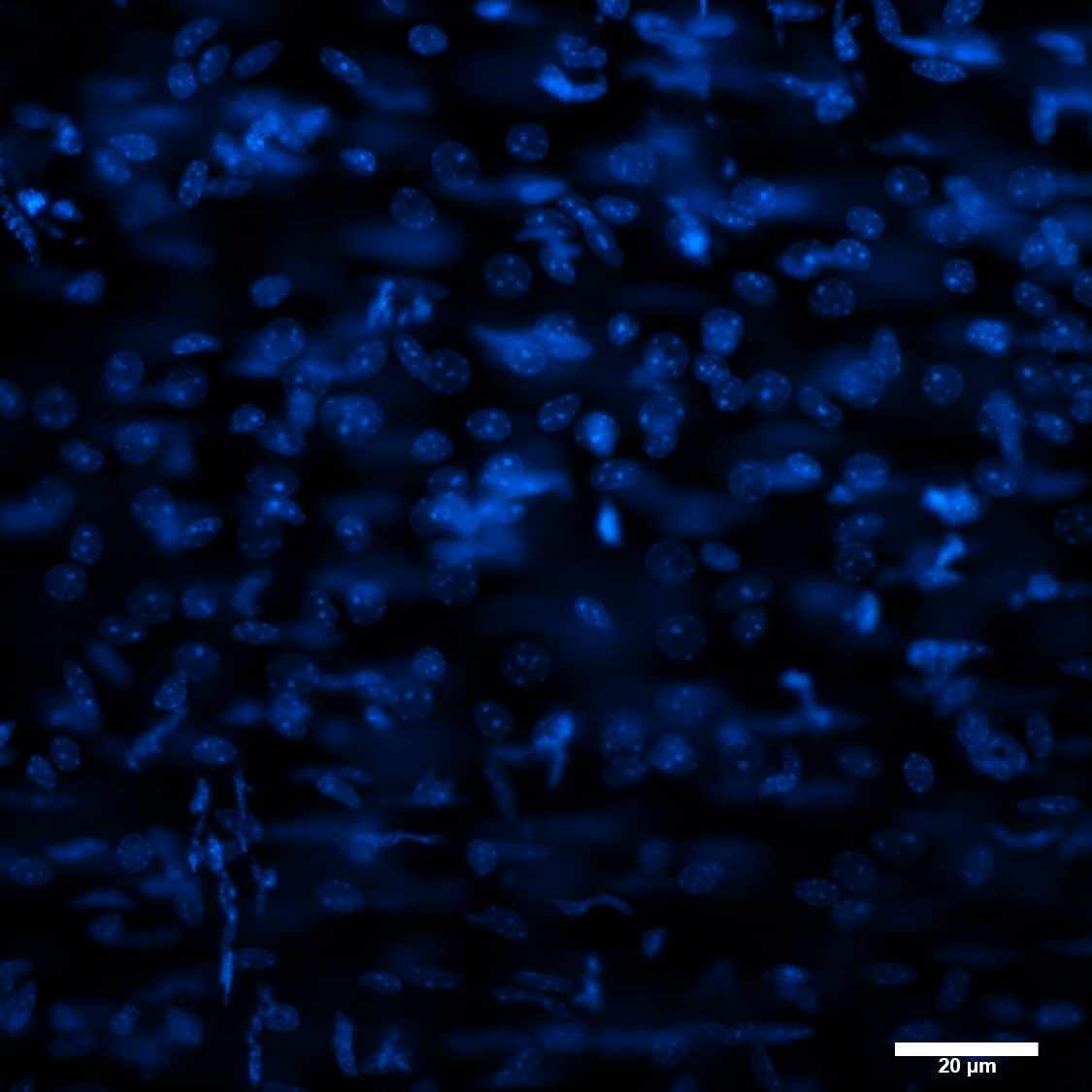

Supplement: Supplementary file 6 [file DataSheet6.zip › Fig 6 original data/Fig 6A/OT-DAPI.tif]

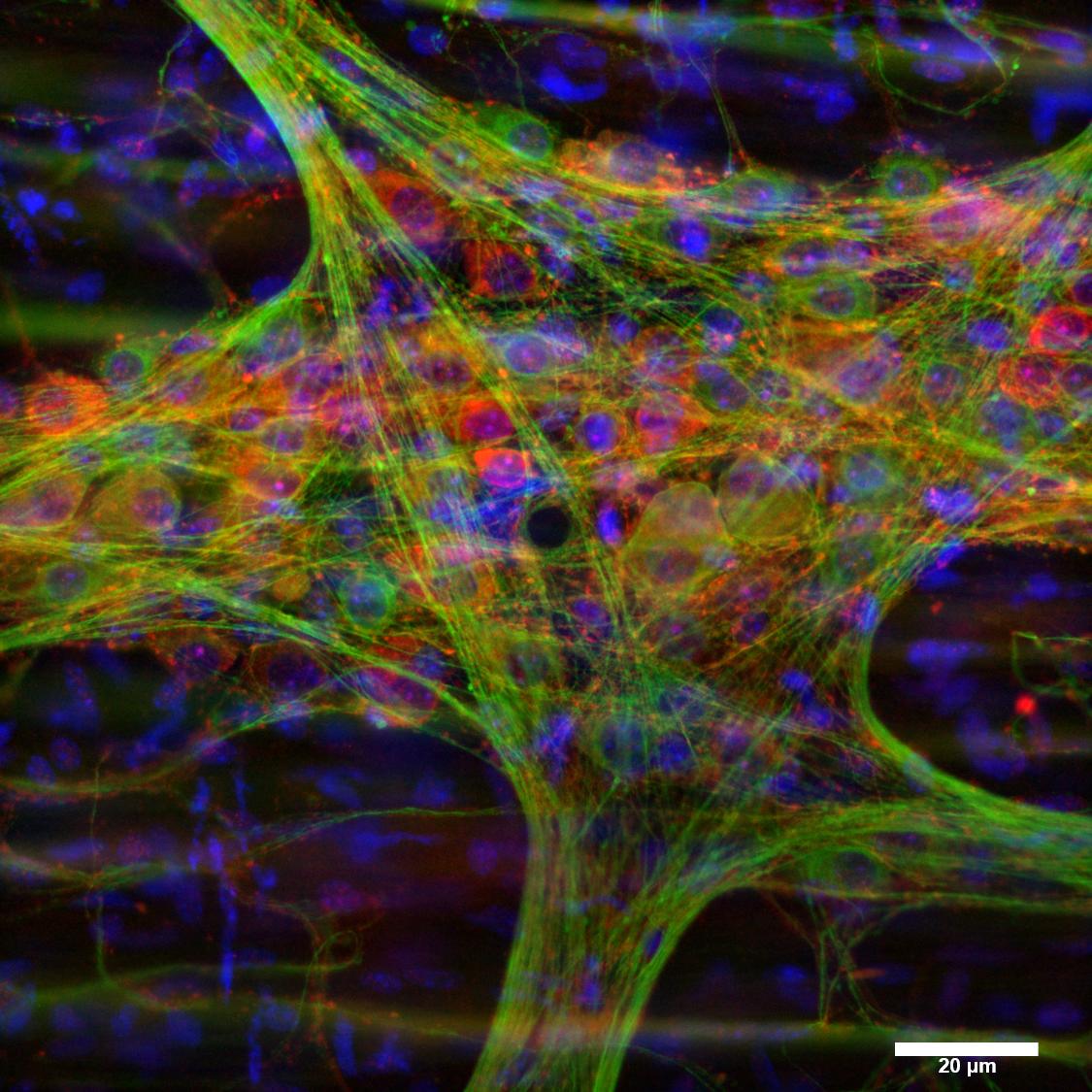

Supplement: Supplementary file 6 [file DataSheet6.zip › Fig 6 original data/Fig 6A/OT-Merge.tif]

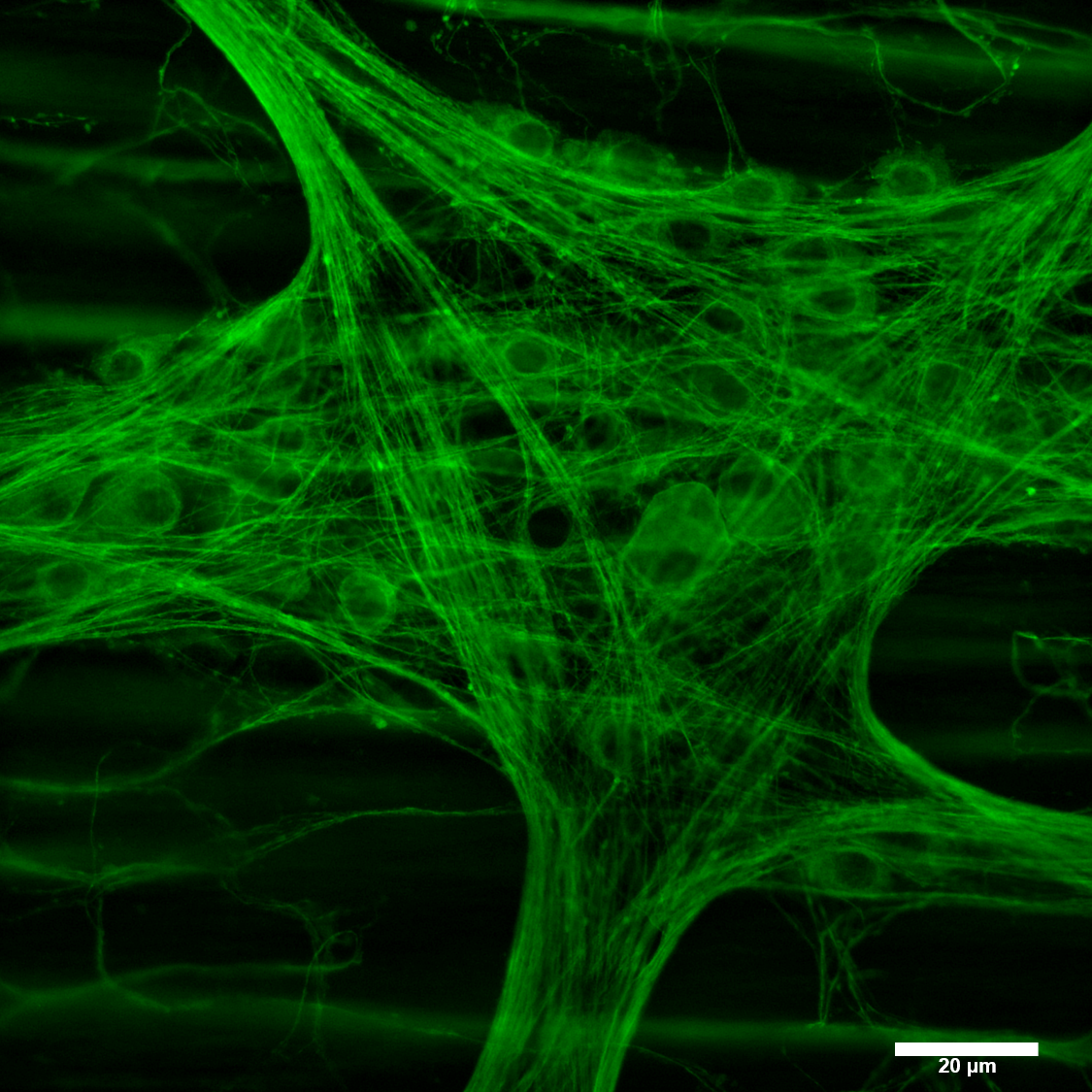

Supplement: Supplementary file 6 [file DataSheet6.zip › Fig 6 original data/Fig 6A/OT-β III Tubulin.tif]

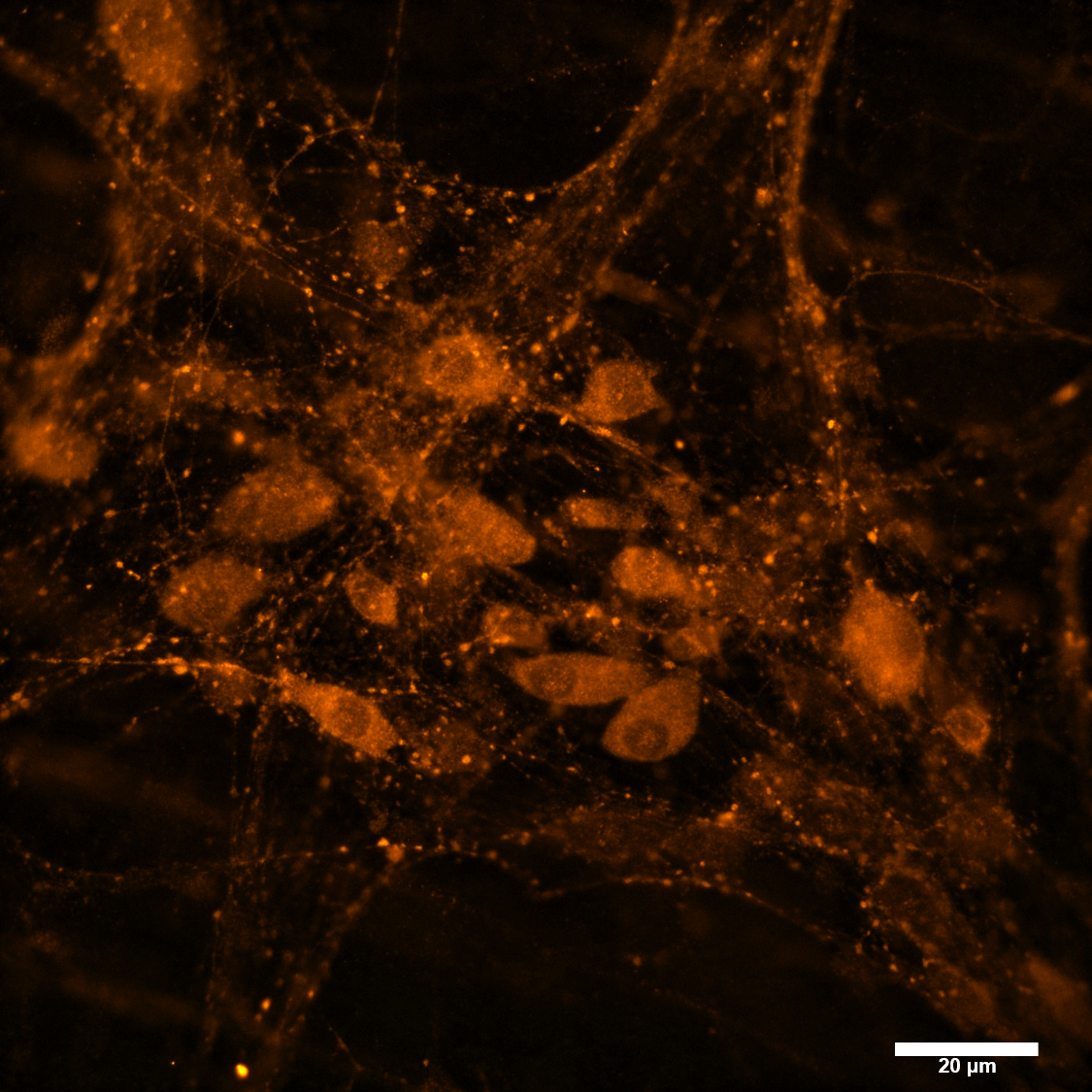

Supplement: Supplementary file 6 [file DataSheet6.zip › Fig 6 original data/Fig 6A/VCR-ChAT.tif]

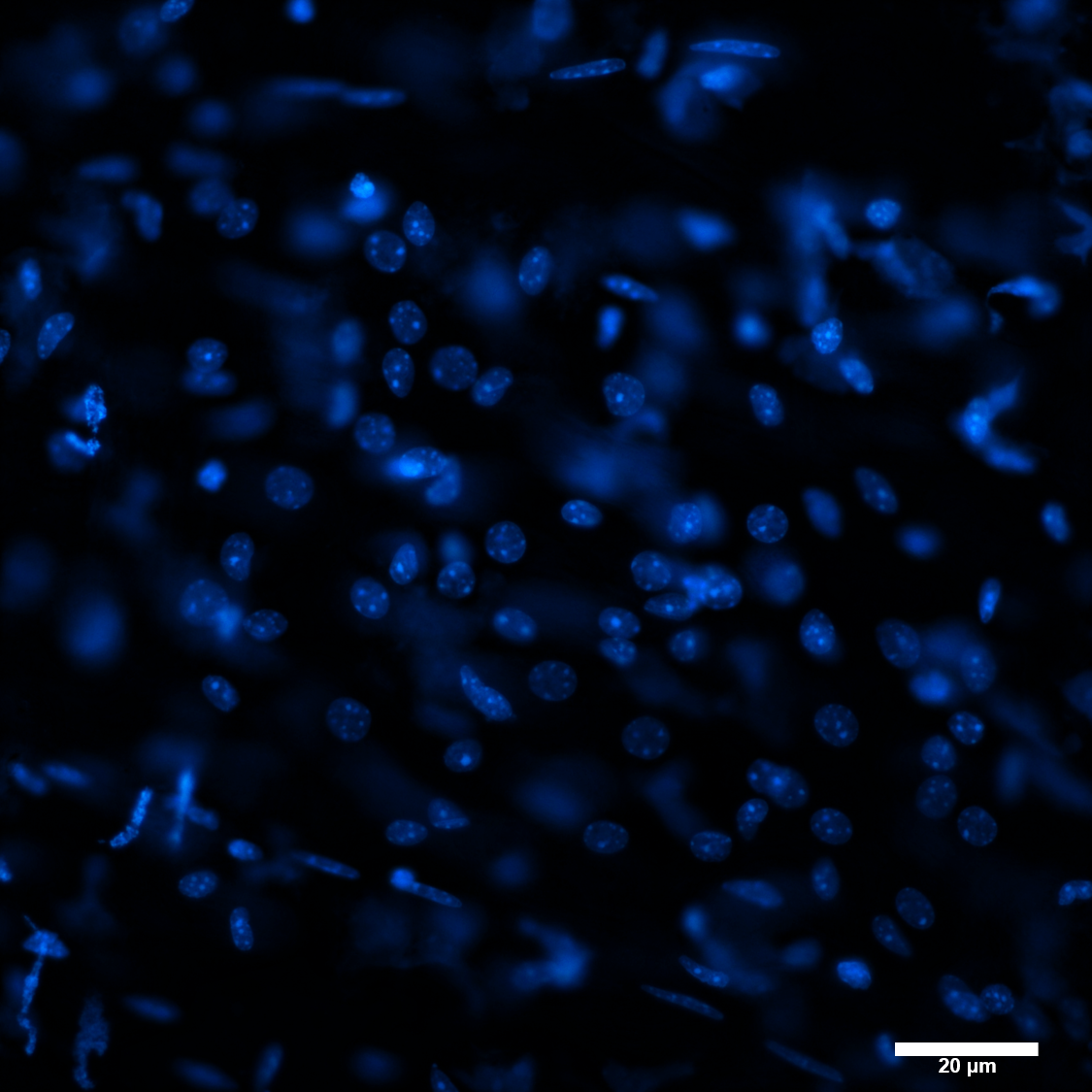

Supplement: Supplementary file 6 [file DataSheet6.zip › Fig 6 original data/Fig 6A/VCR-DAPI.tif]

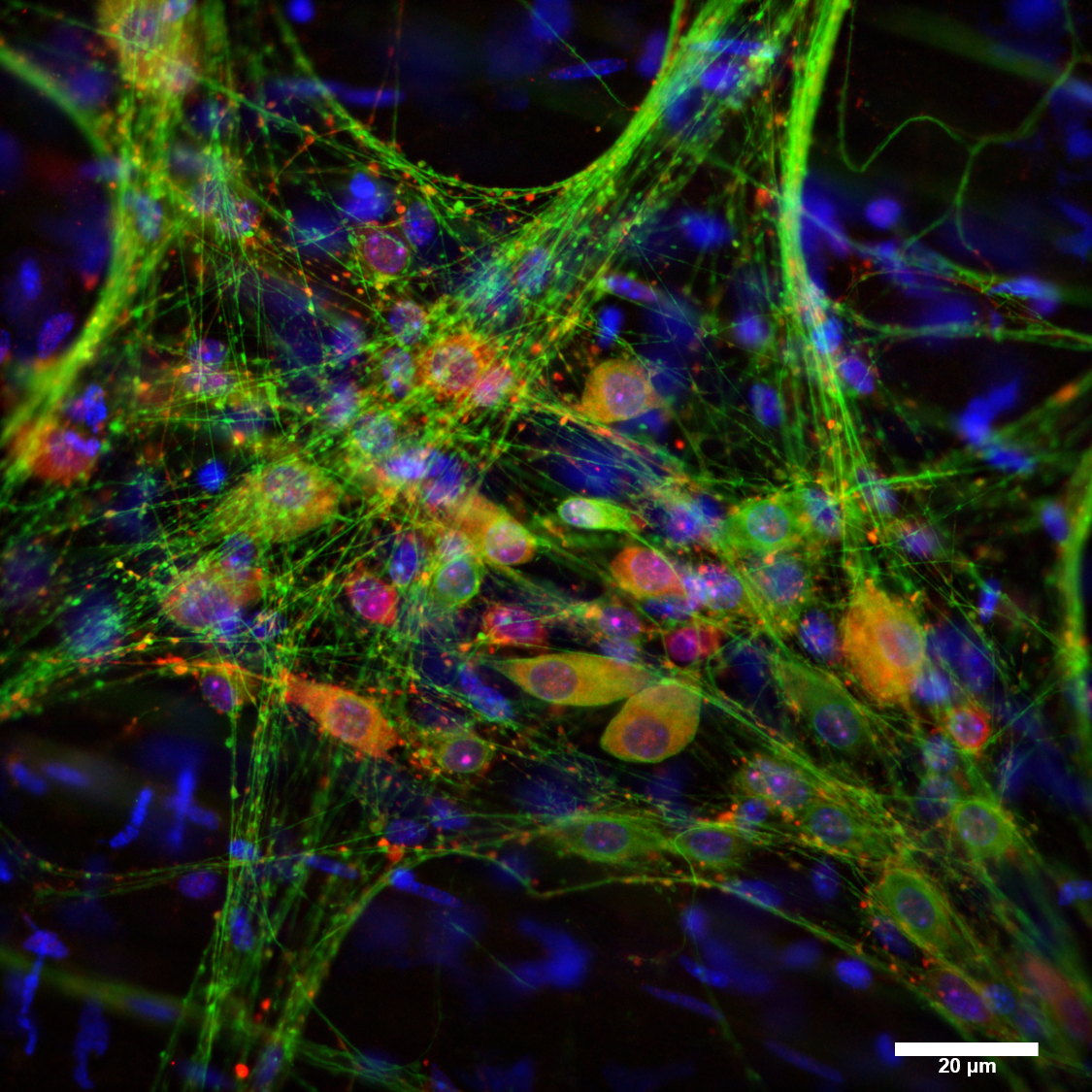

Supplement: Supplementary file 6 [file DataSheet6.zip › Fig 6 original data/Fig 6A/VCR-Merge.tif]

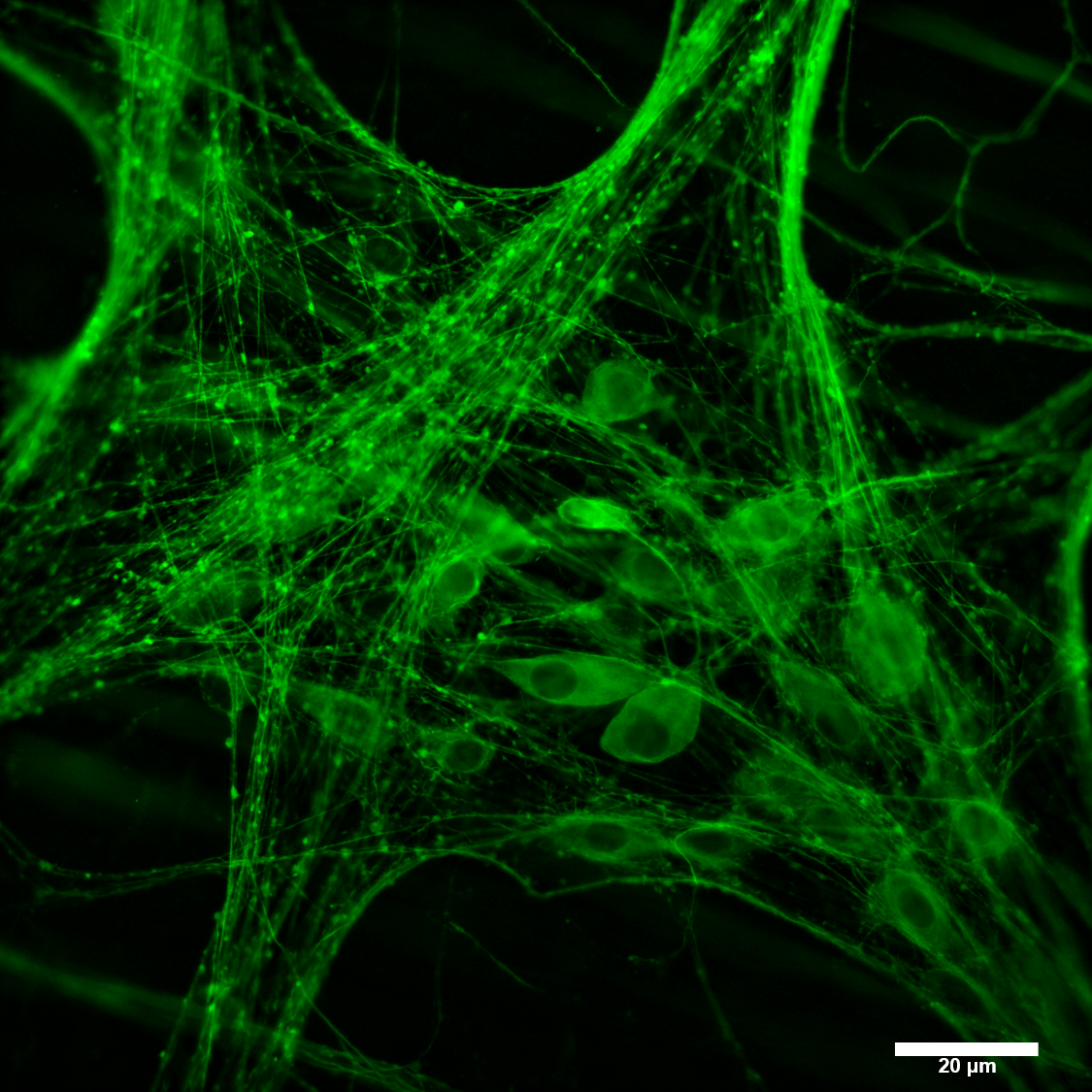

Supplement: Supplementary file 6 [file DataSheet6.zip › Fig 6 original data/Fig 6A/VCR-β III Tubulin.tif]

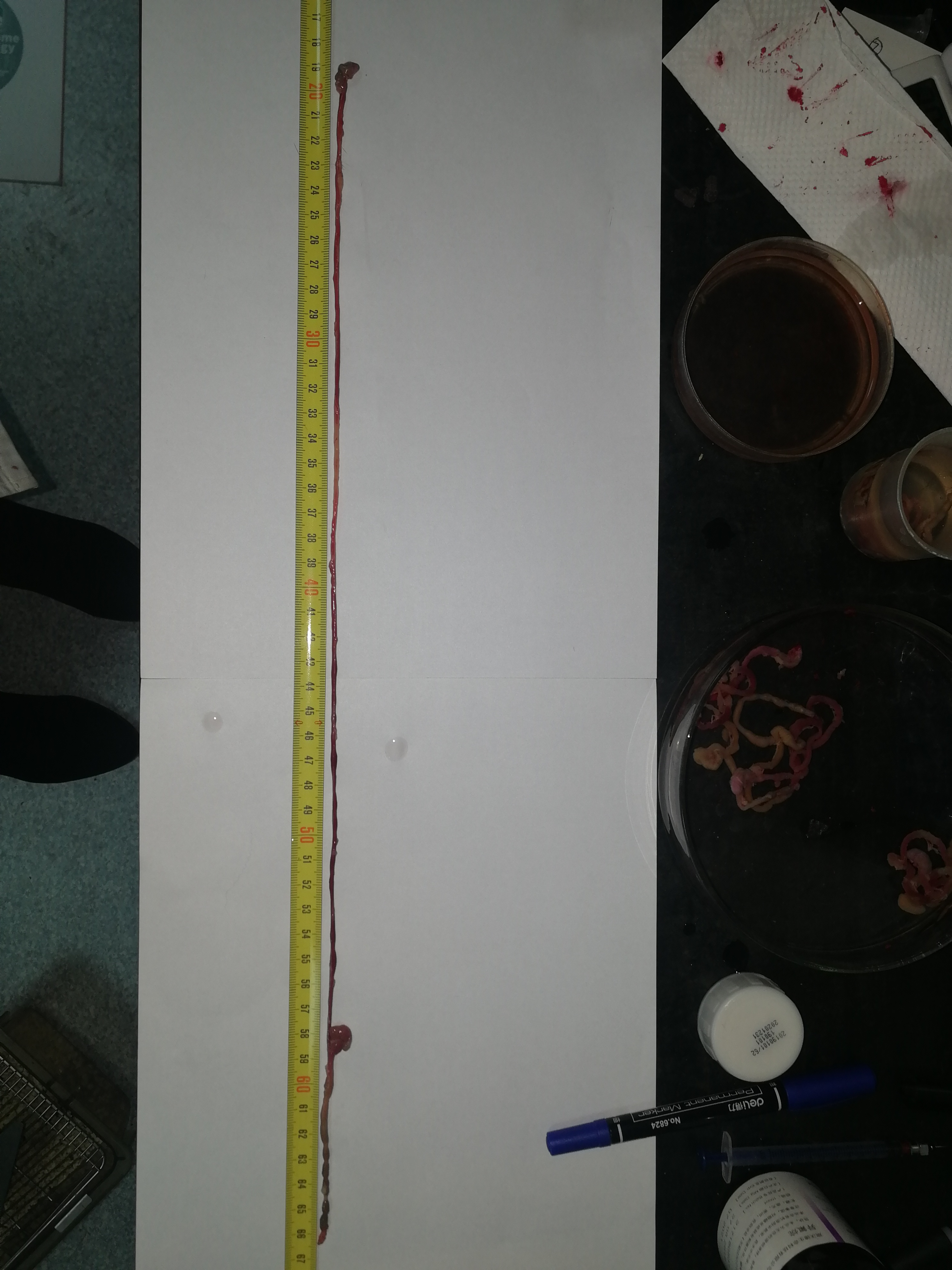

Supplement: Supplementary file 7 [file DataSheet2.zip › Fig 2 original data/Fig 2B/NS.jpg]

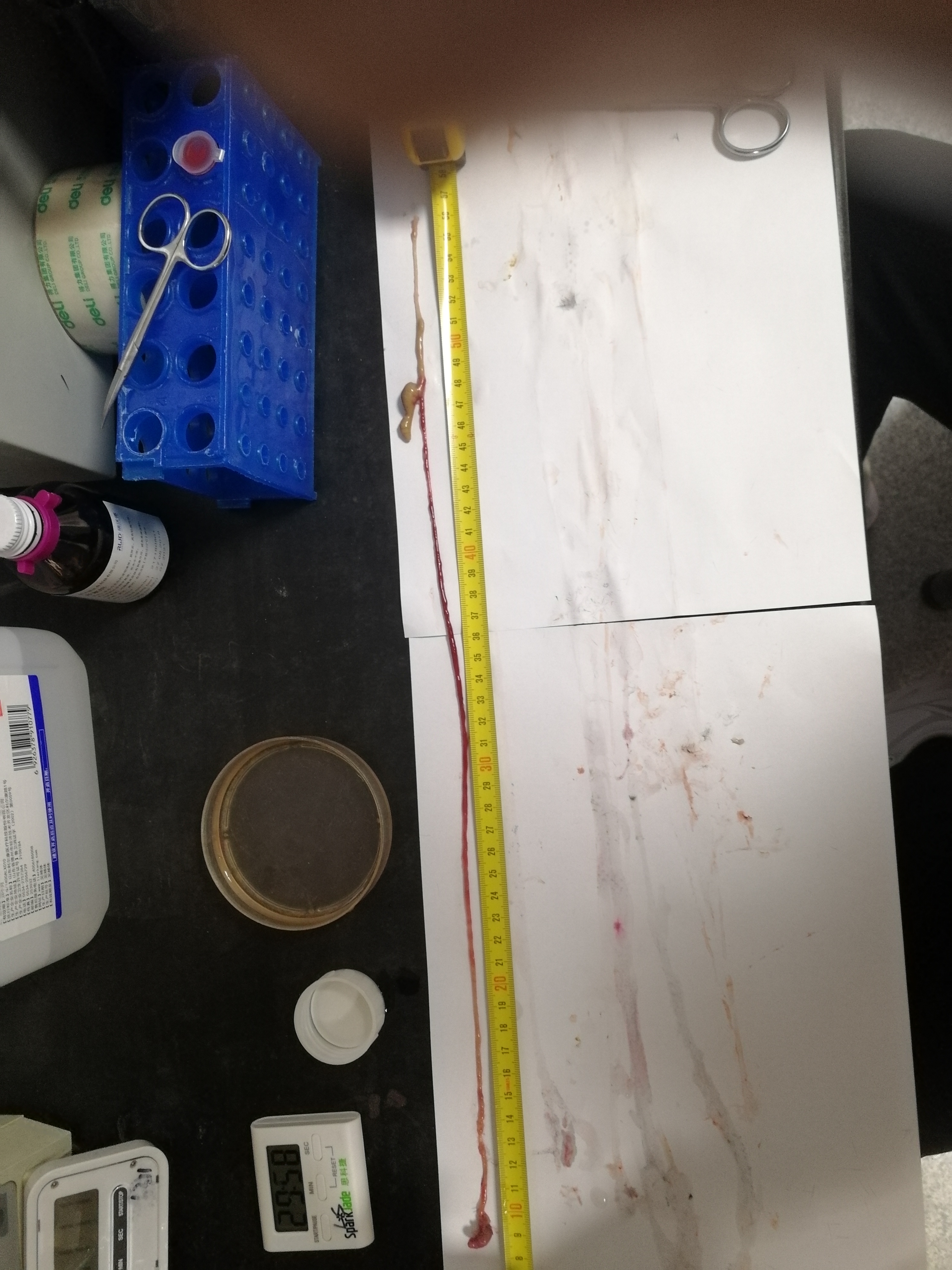

Supplement: Supplementary file 7 [file DataSheet2.zip › Fig 2 original data/Fig 2B/OT+VCR.jpg]

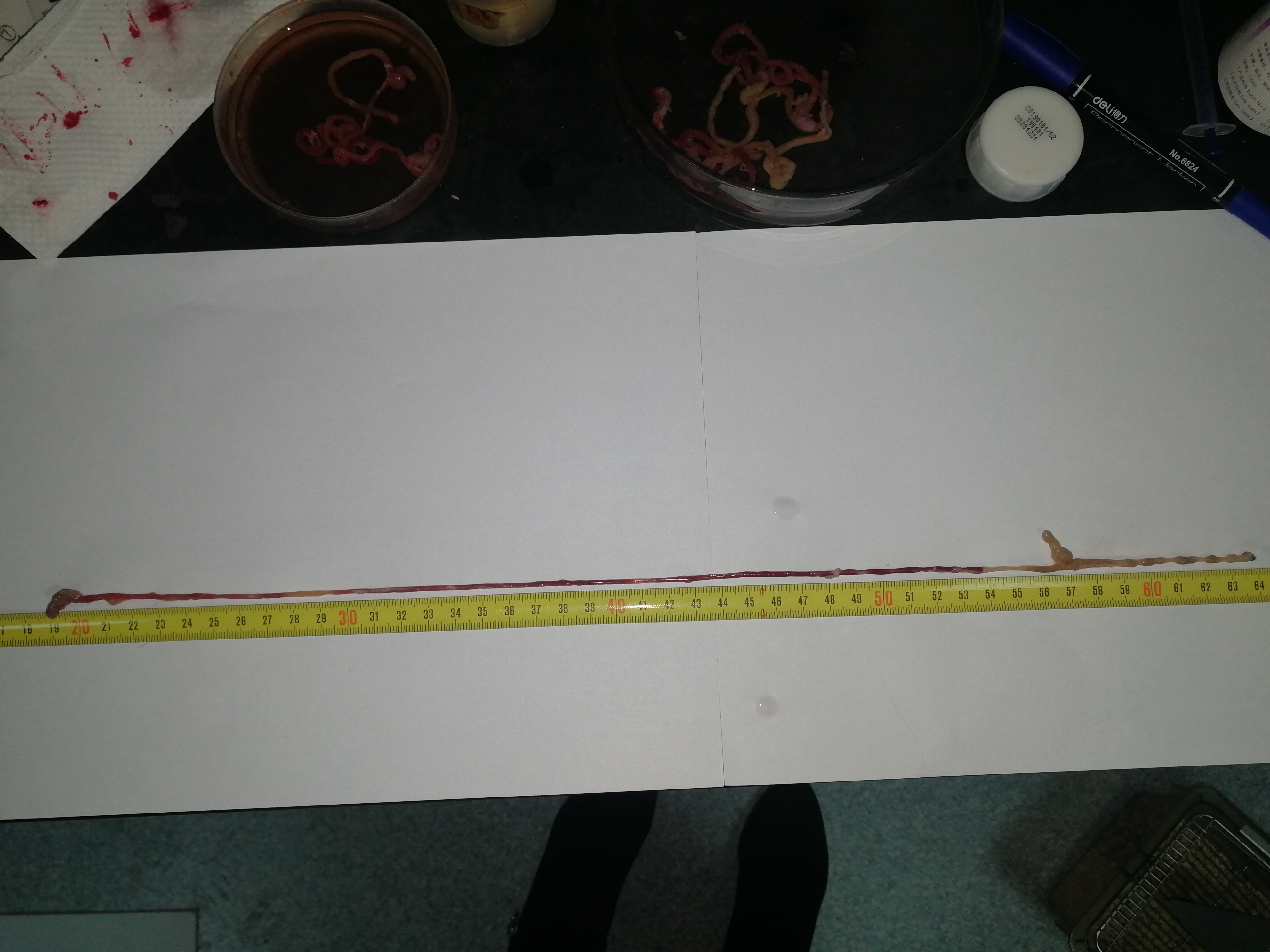

Supplement: Supplementary file 7 [file DataSheet2.zip › Fig 2 original data/Fig 2B/OT.jpg]

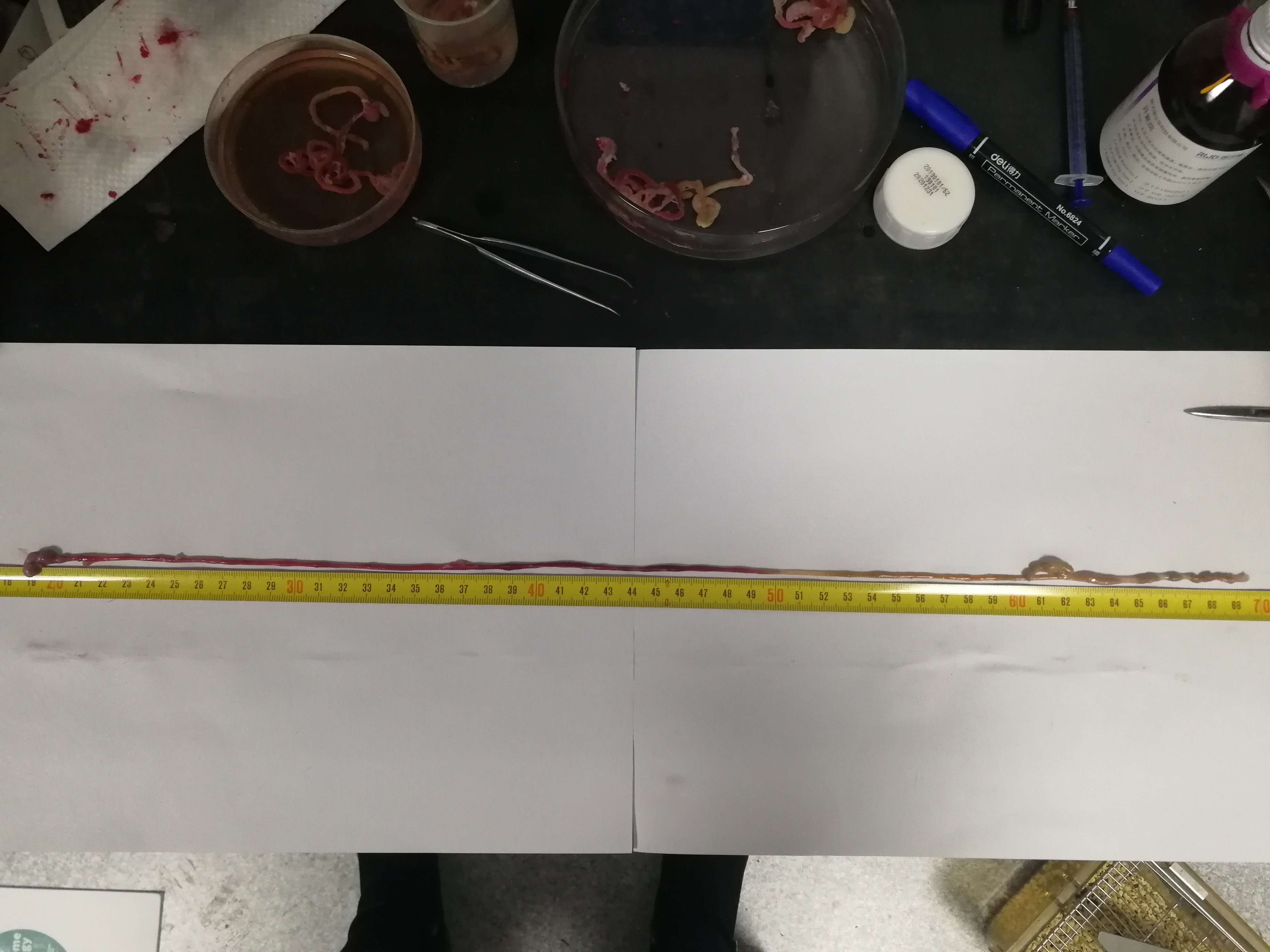

Supplement: Supplementary file 7 [file DataSheet2.zip › Fig 2 original data/Fig 2B/VCR.jpg]

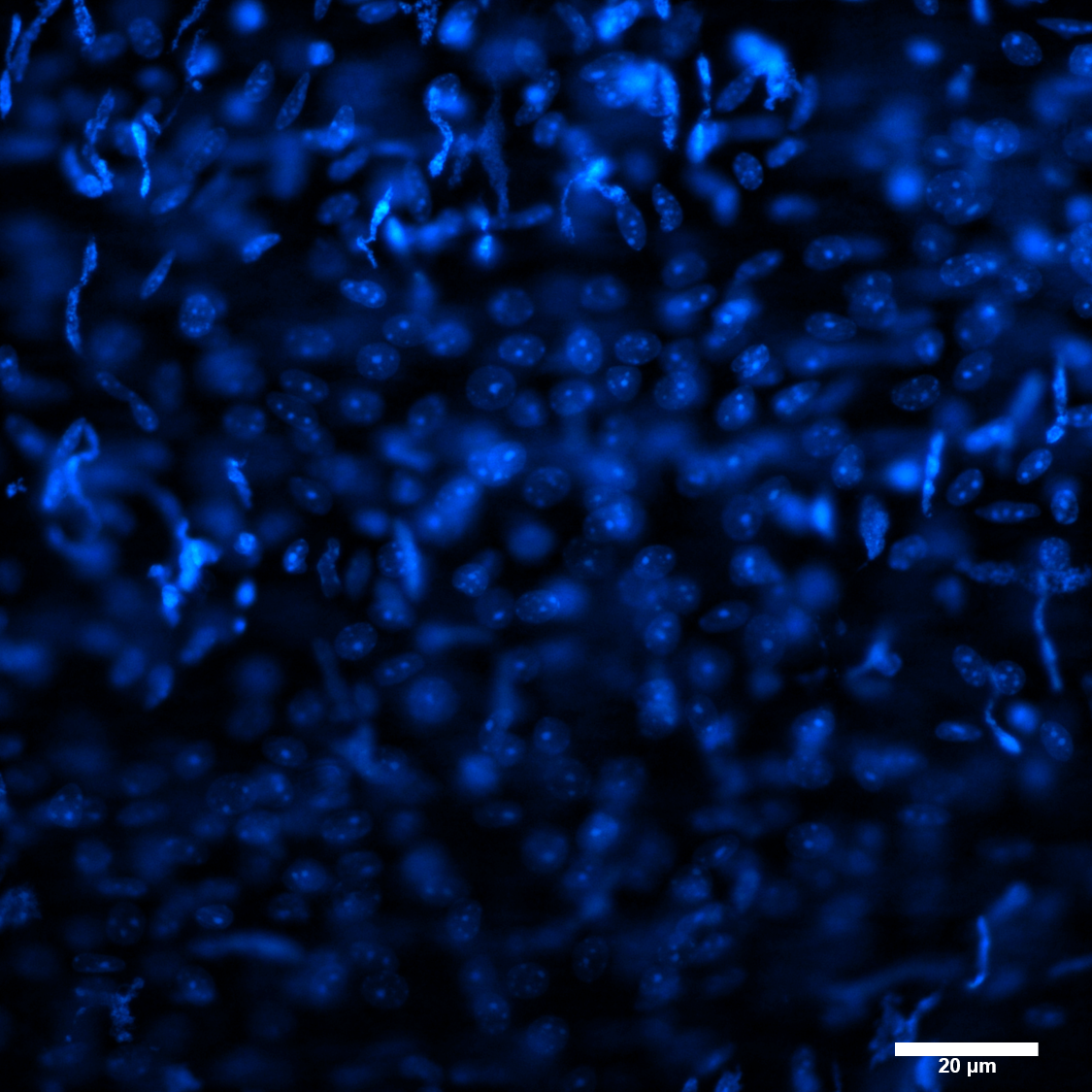

Supplement: Supplementary file 8 [file DataSheet5.zip › Fig 5 original data/Fig 5A/NS-DAPI.tif]

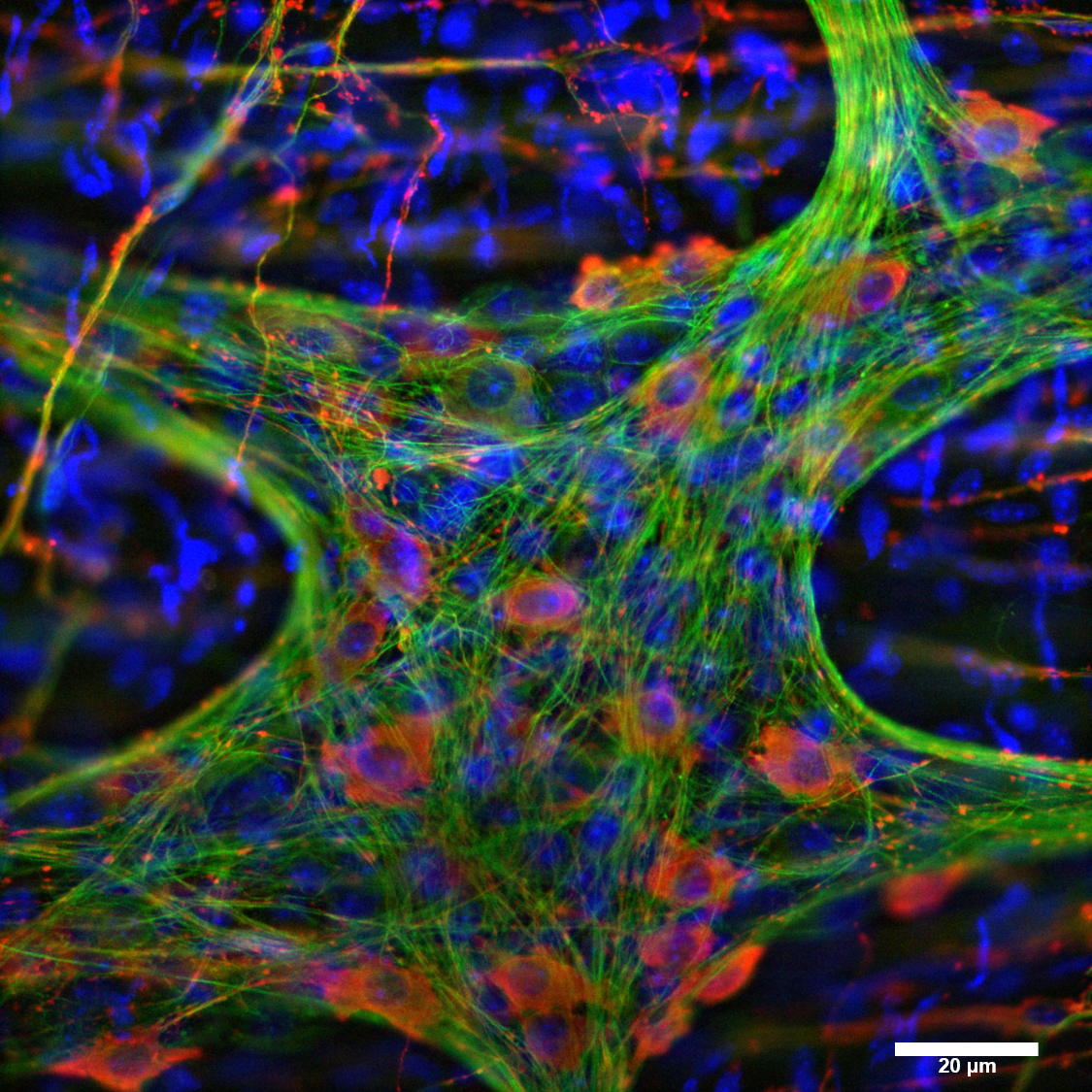

Supplement: Supplementary file 8 [file DataSheet5.zip › Fig 5 original data/Fig 5A/NS-Merge.tif]

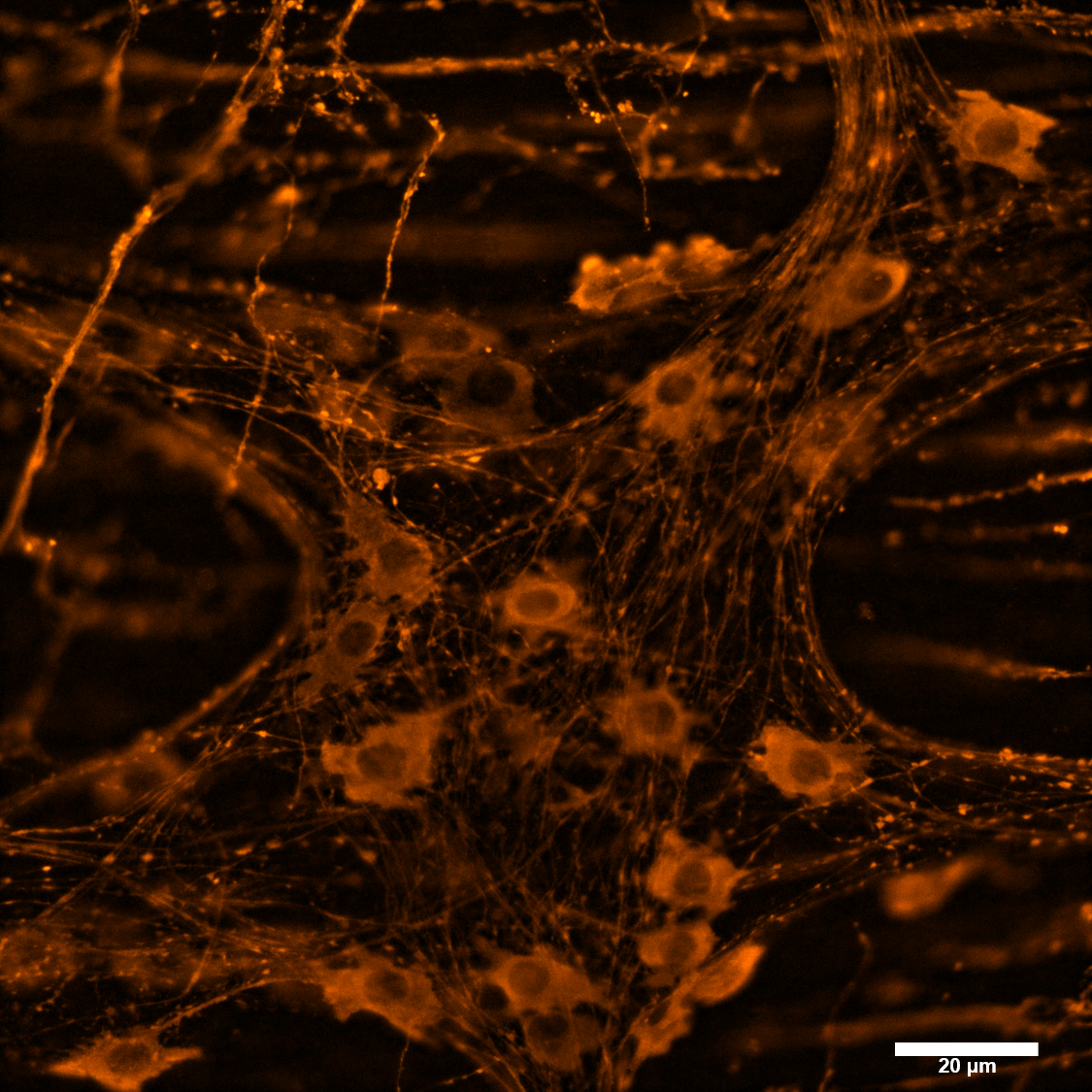

Supplement: Supplementary file 8 [file DataSheet5.zip › Fig 5 original data/Fig 5A/NS-nNOS.tif]

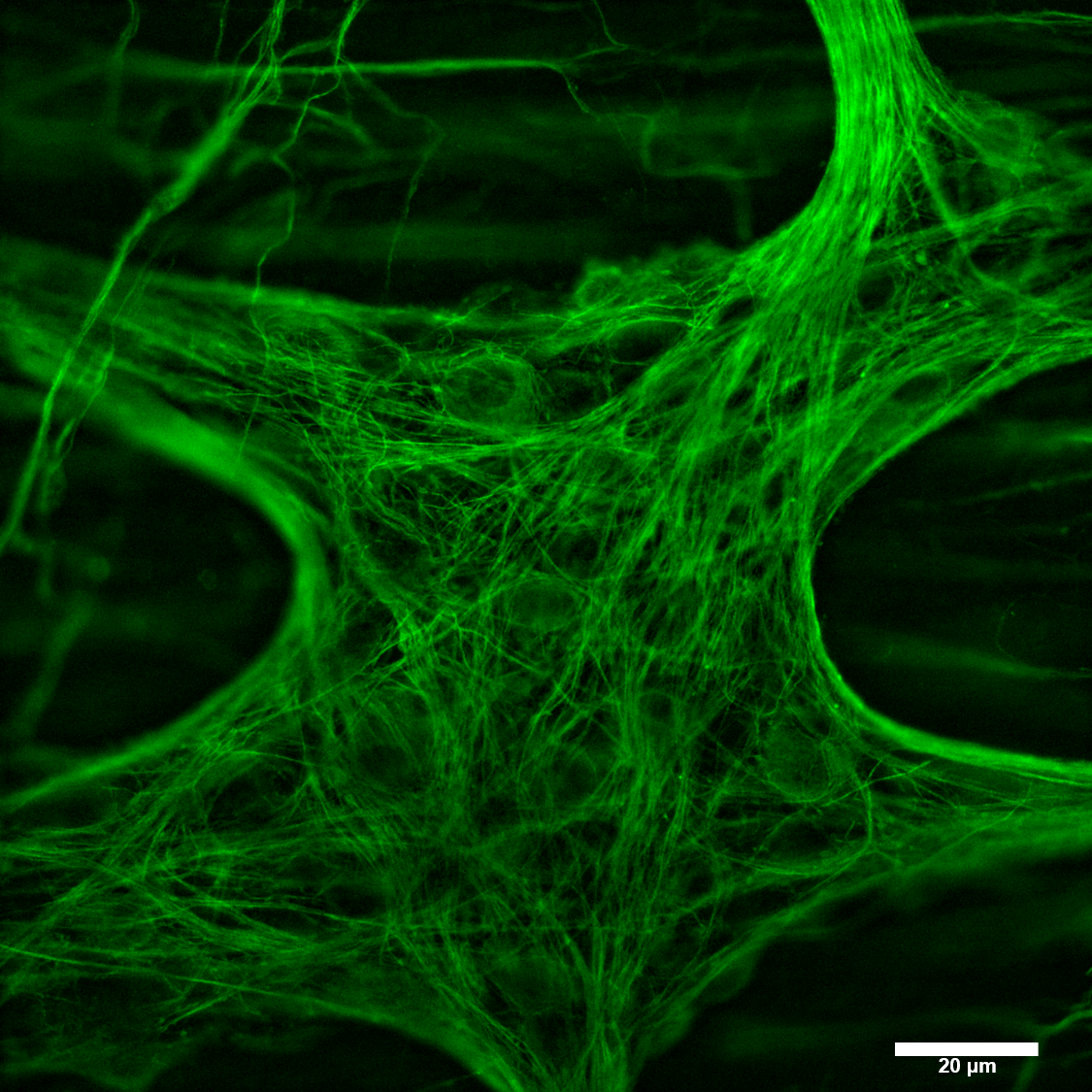

Supplement: Supplementary file 8 [file DataSheet5.zip › Fig 5 original data/Fig 5A/NS-β III Tubulin.tif]

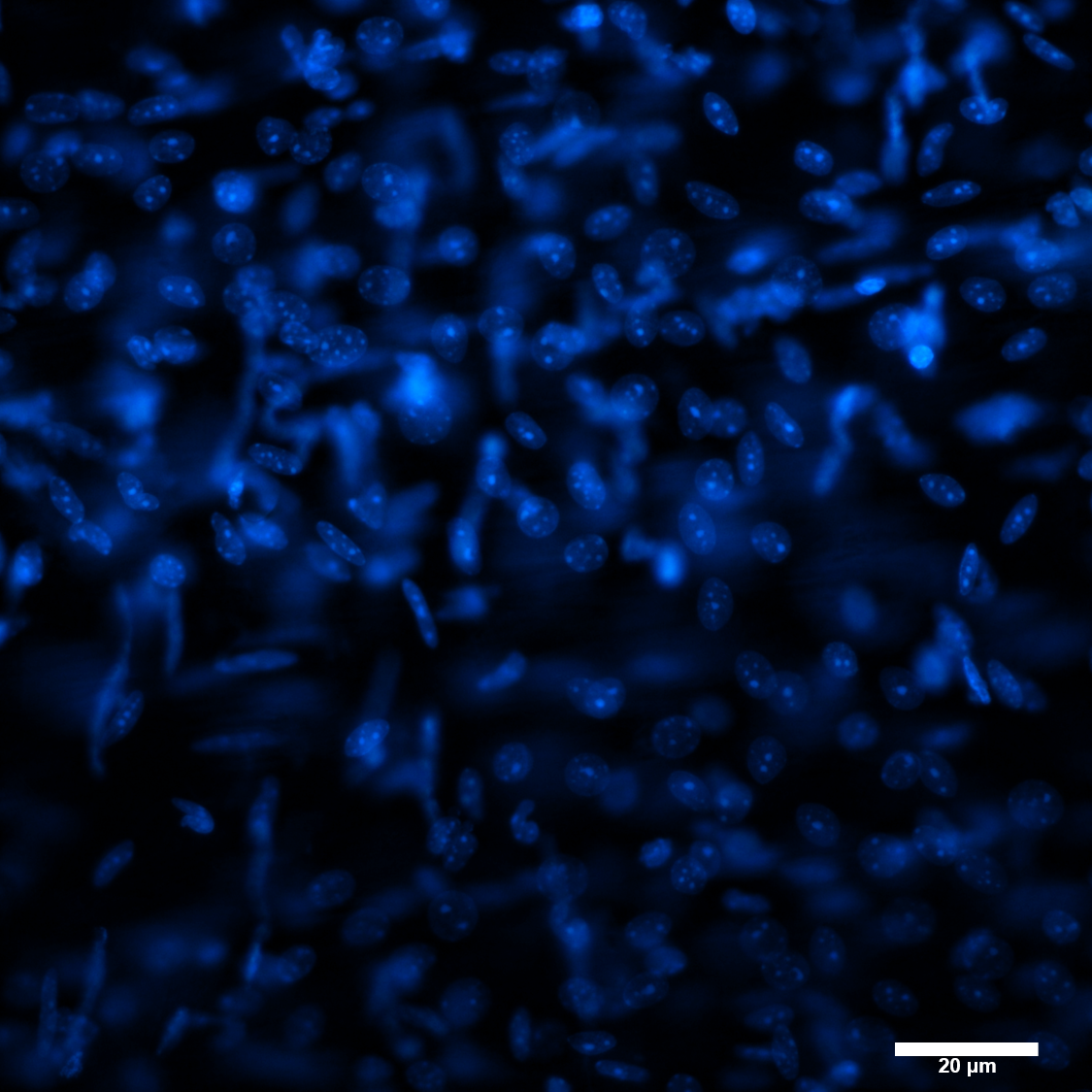

Supplement: Supplementary file 8 [file DataSheet5.zip › Fig 5 original data/Fig 5A/OT+VCR-DAPI.tif]

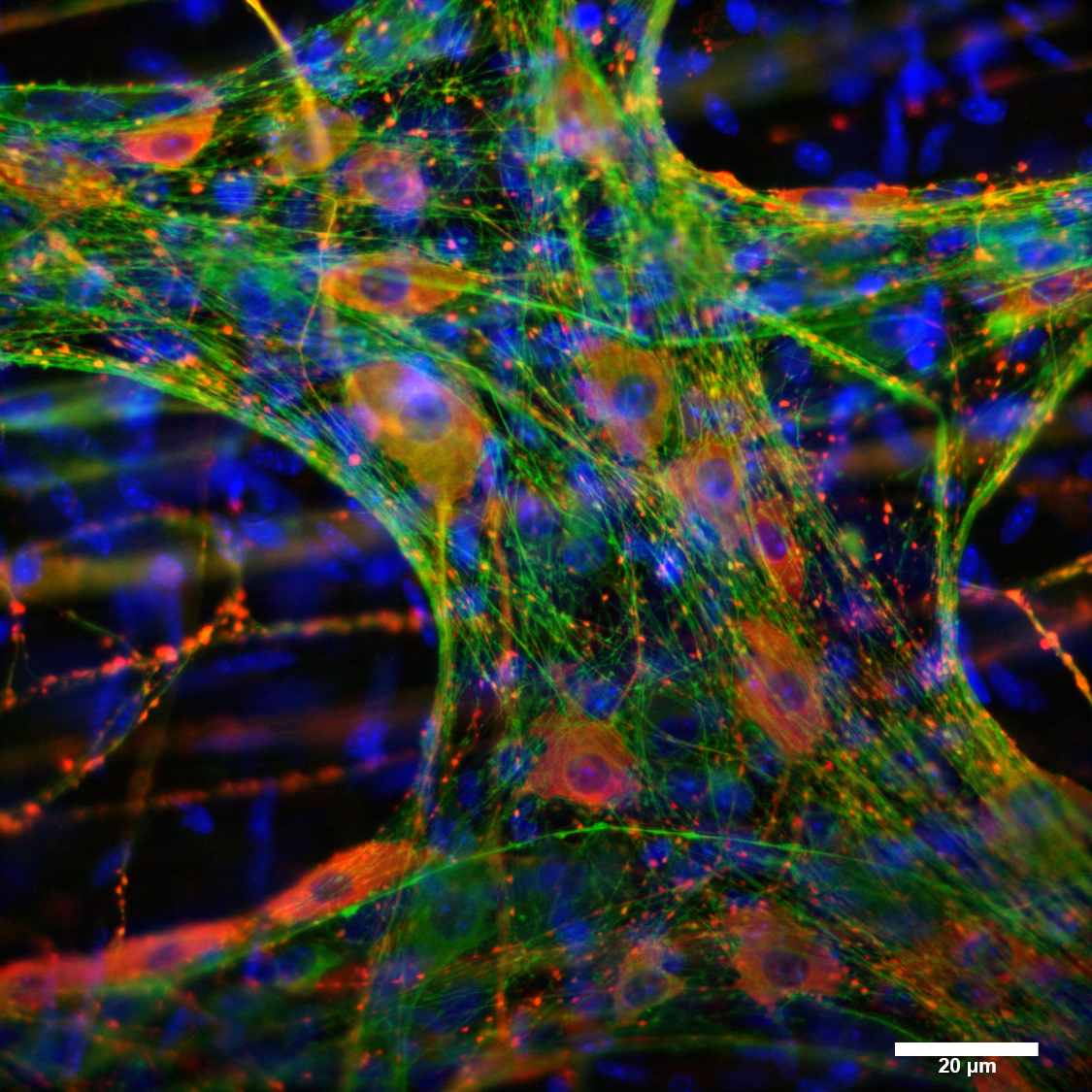

Supplement: Supplementary file 8 [file DataSheet5.zip › Fig 5 original data/Fig 5A/OT+VCR-Merge.tif]

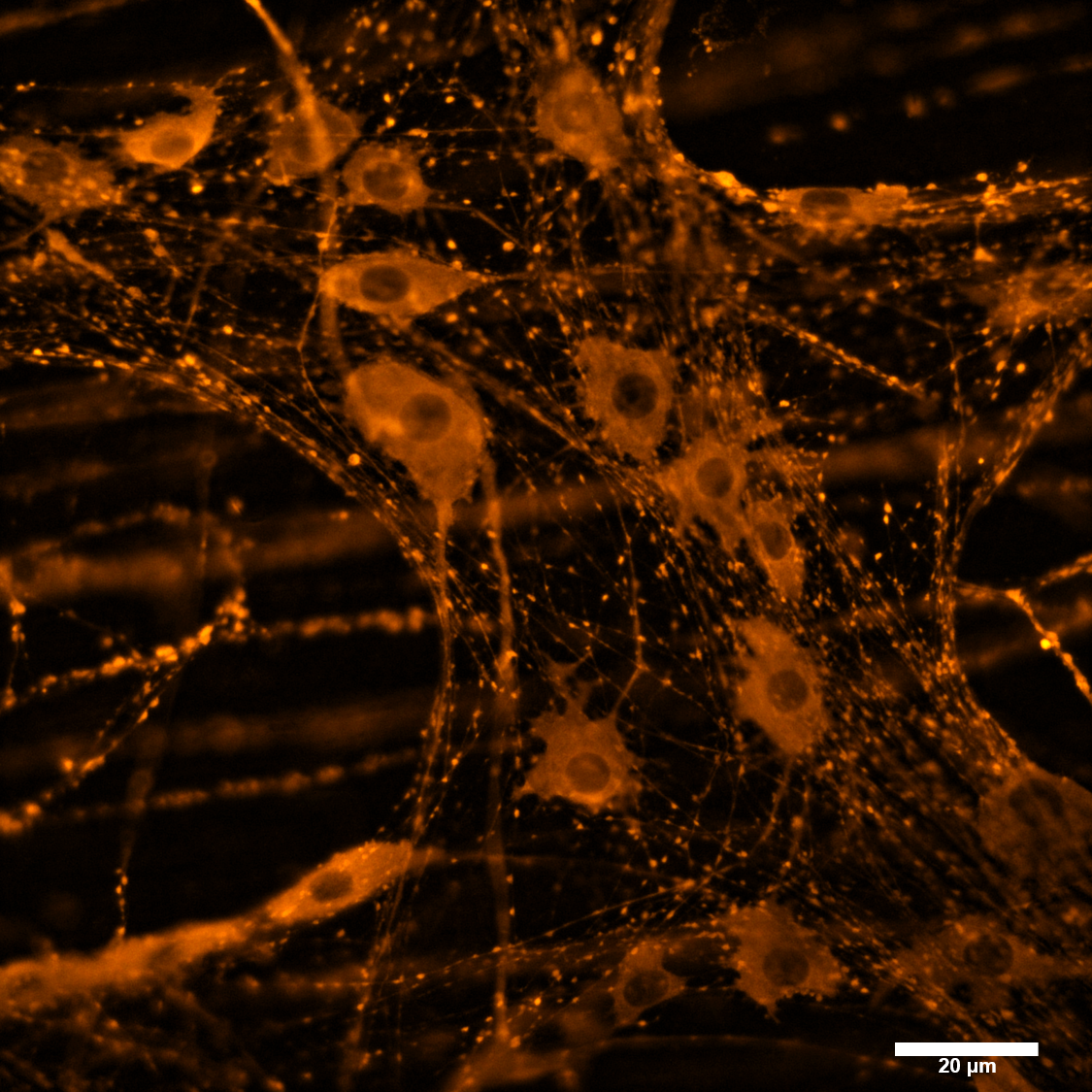

Supplement: Supplementary file 8 [file DataSheet5.zip › Fig 5 original data/Fig 5A/OT+VCR-nNOS.tif]

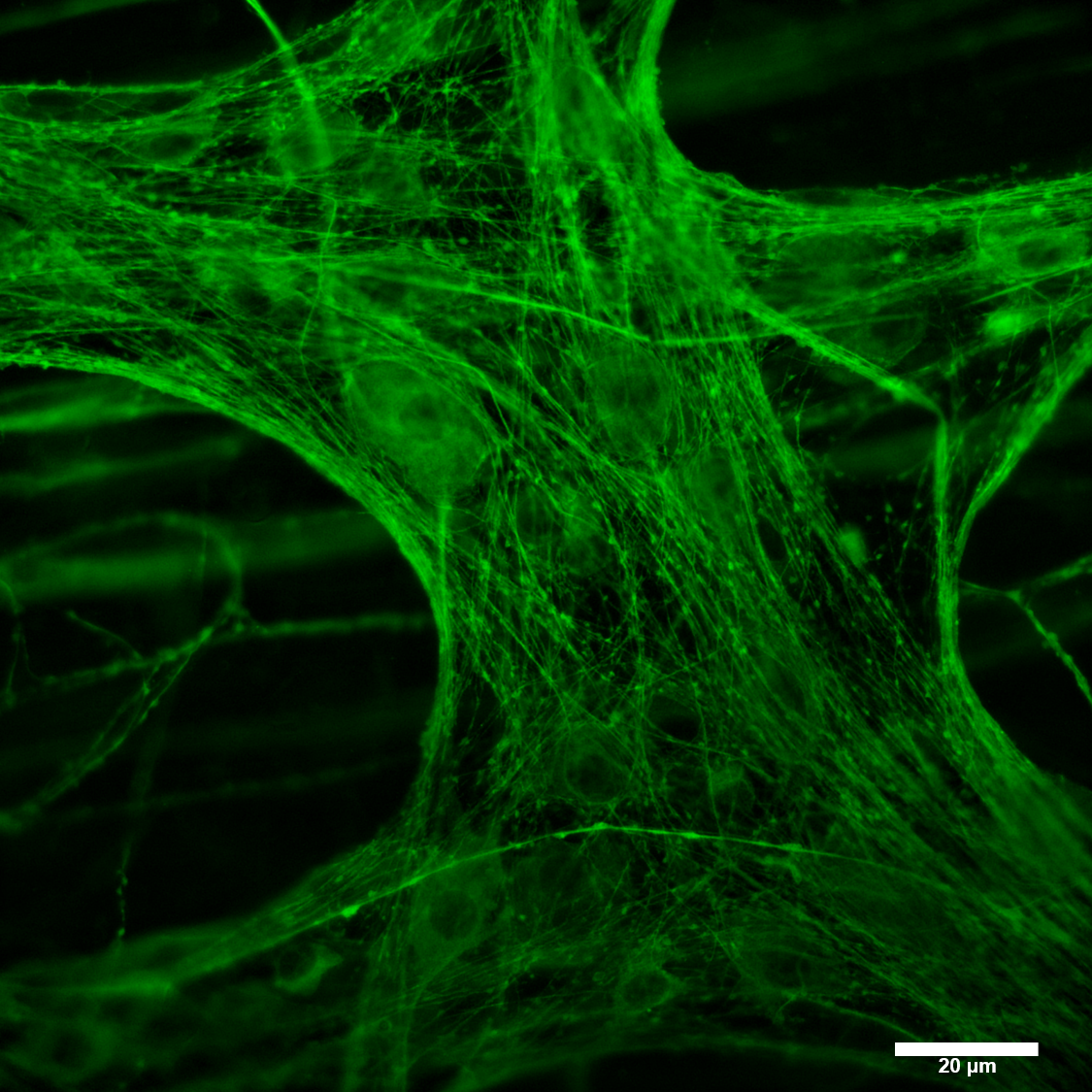

Supplement: Supplementary file 8 [file DataSheet5.zip › Fig 5 original data/Fig 5A/OT+VCR-β III Tubulin.tif]

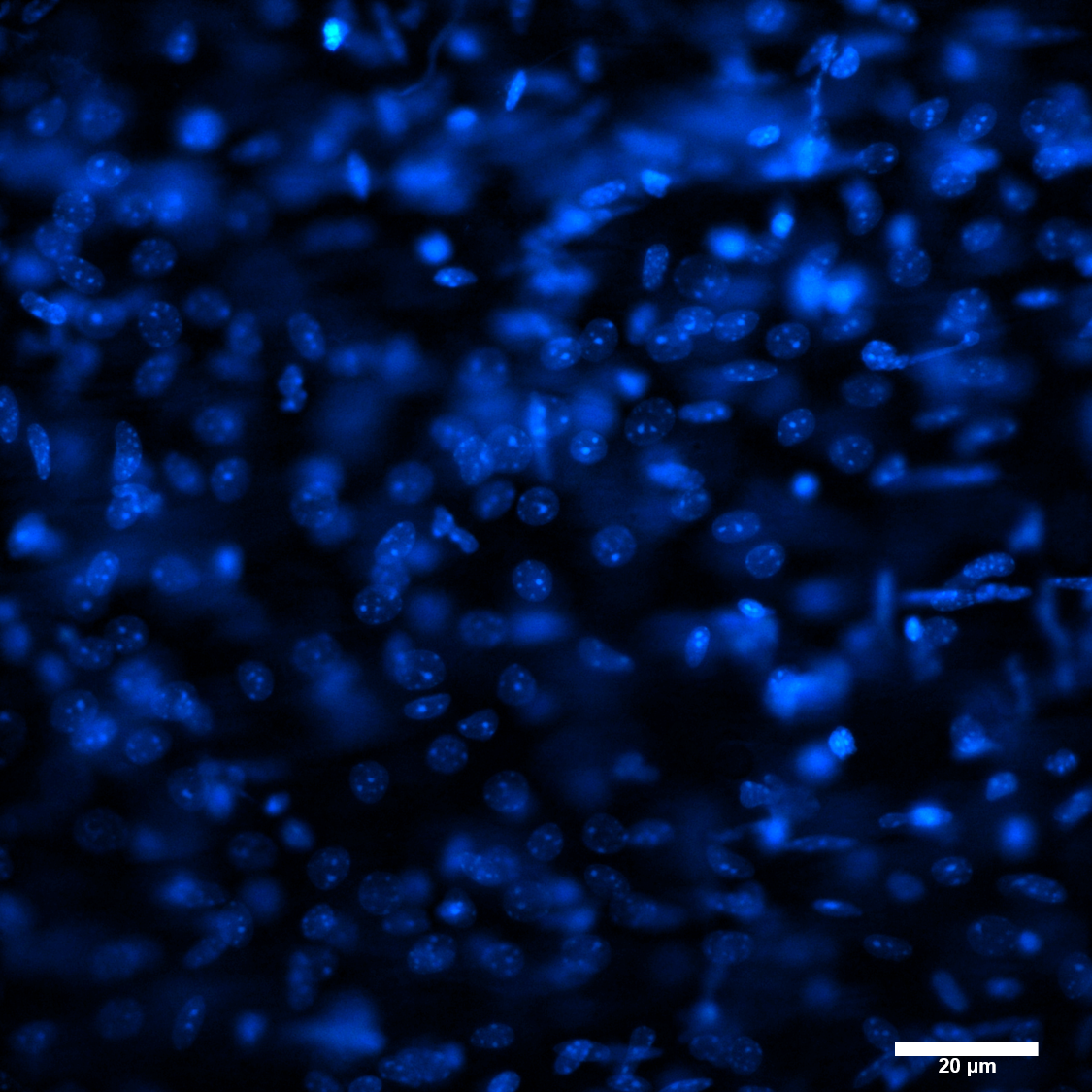

Supplement: Supplementary file 8 [file DataSheet5.zip › Fig 5 original data/Fig 5A/OT-DAPI.tif]

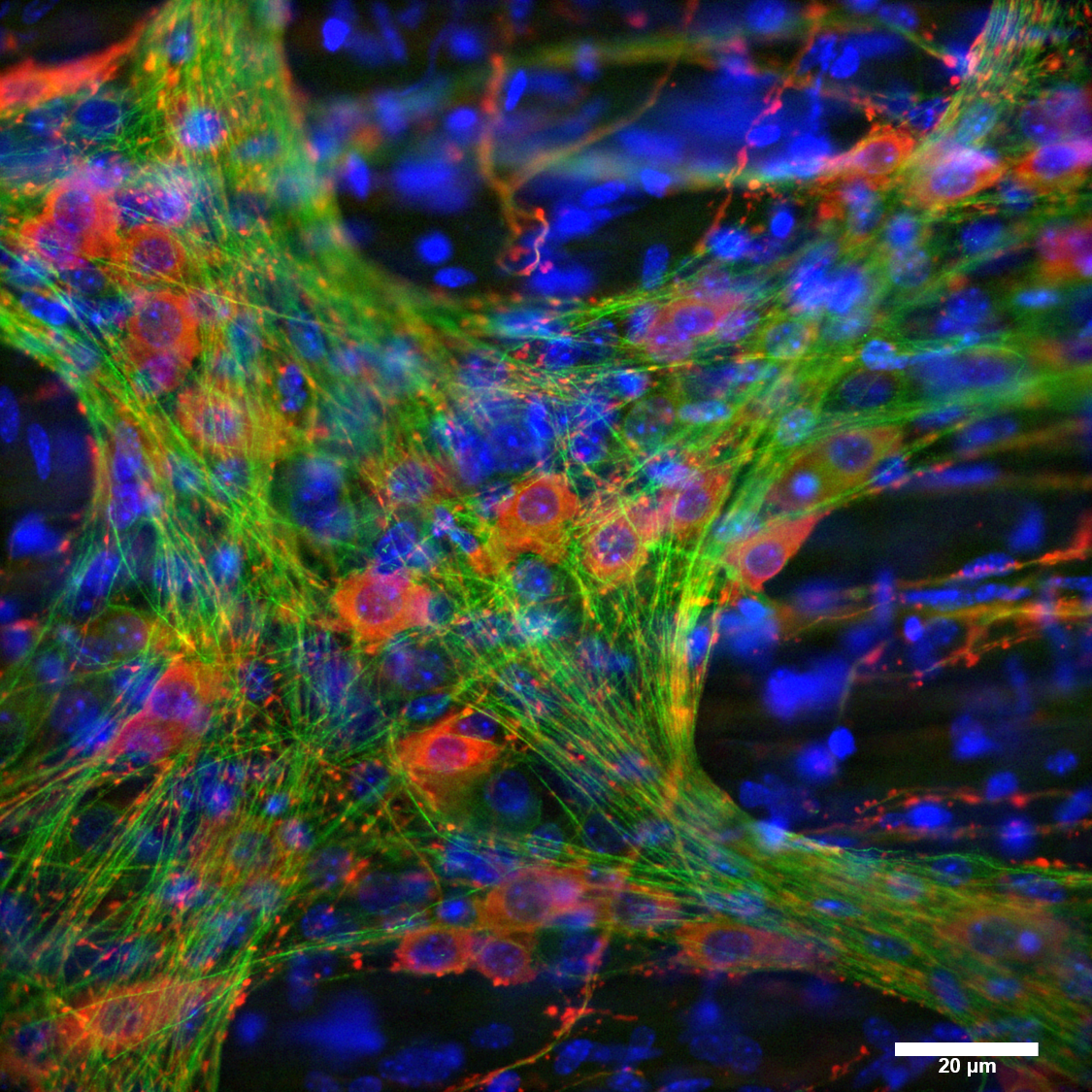

Supplement: Supplementary file 8 [file DataSheet5.zip › Fig 5 original data/Fig 5A/OT-Merge.tif]

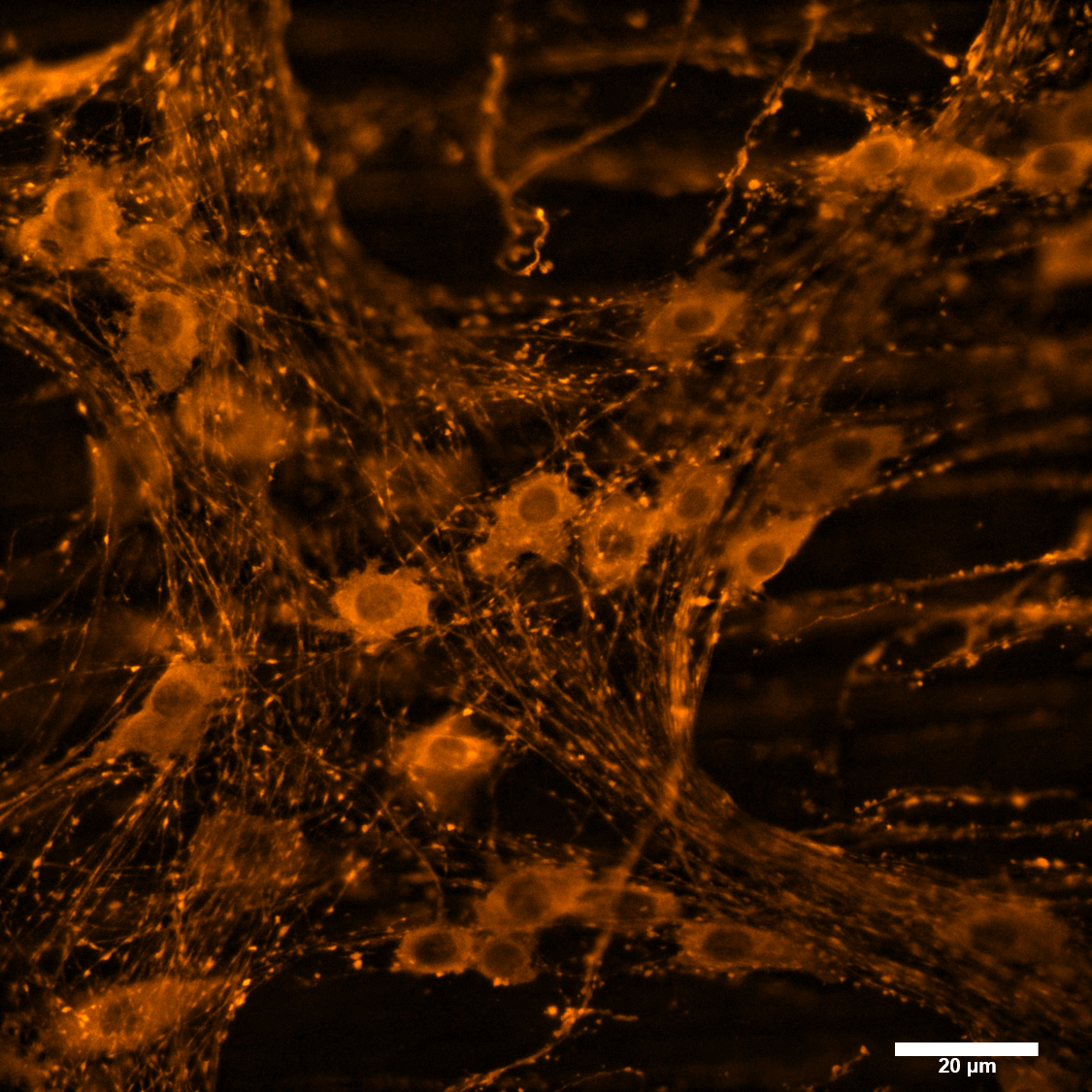

Supplement: Supplementary file 8 [file DataSheet5.zip › Fig 5 original data/Fig 5A/OT-nNOS.tif]

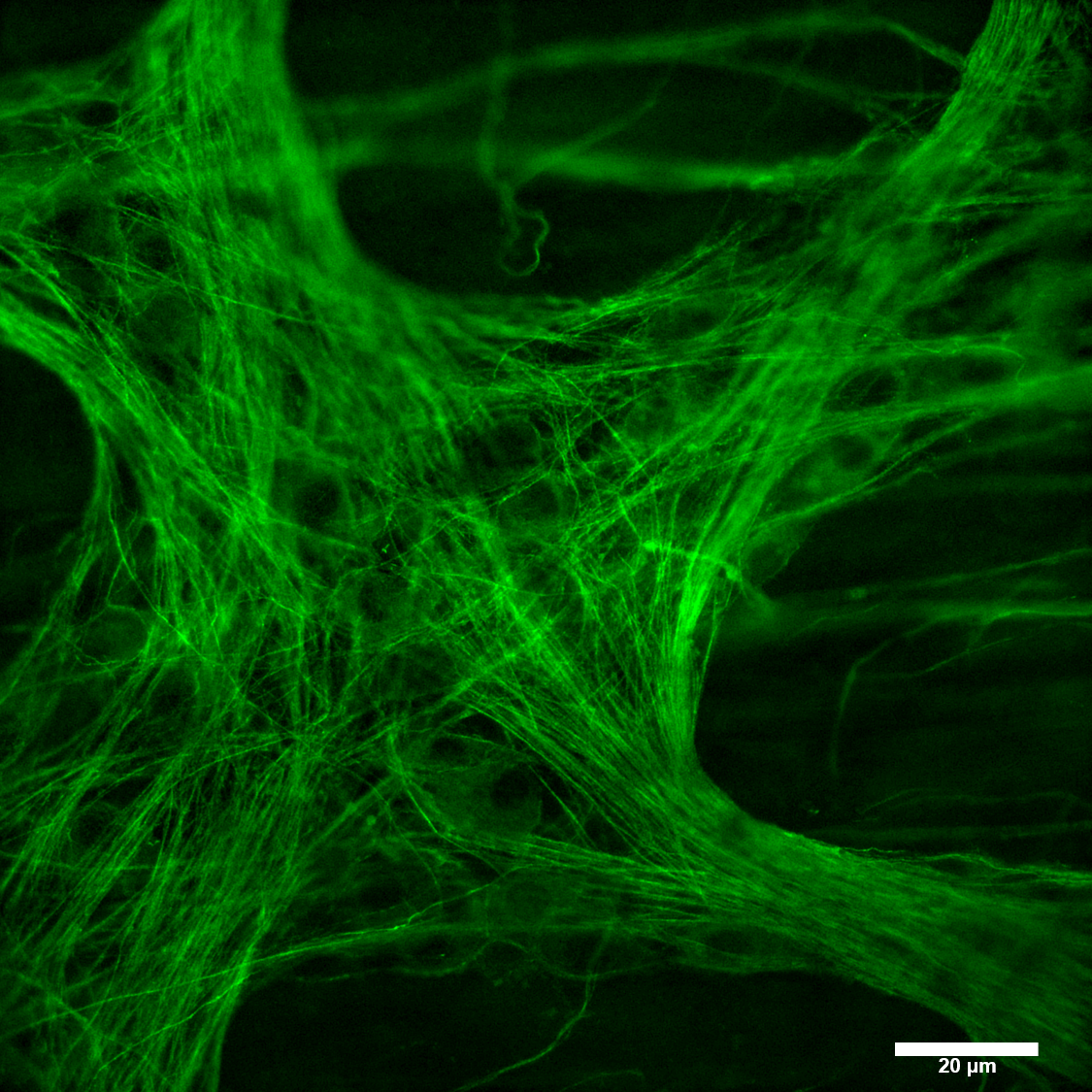

Supplement: Supplementary file 8 [file DataSheet5.zip › Fig 5 original data/Fig 5A/OT-β III Tubulin.tif]

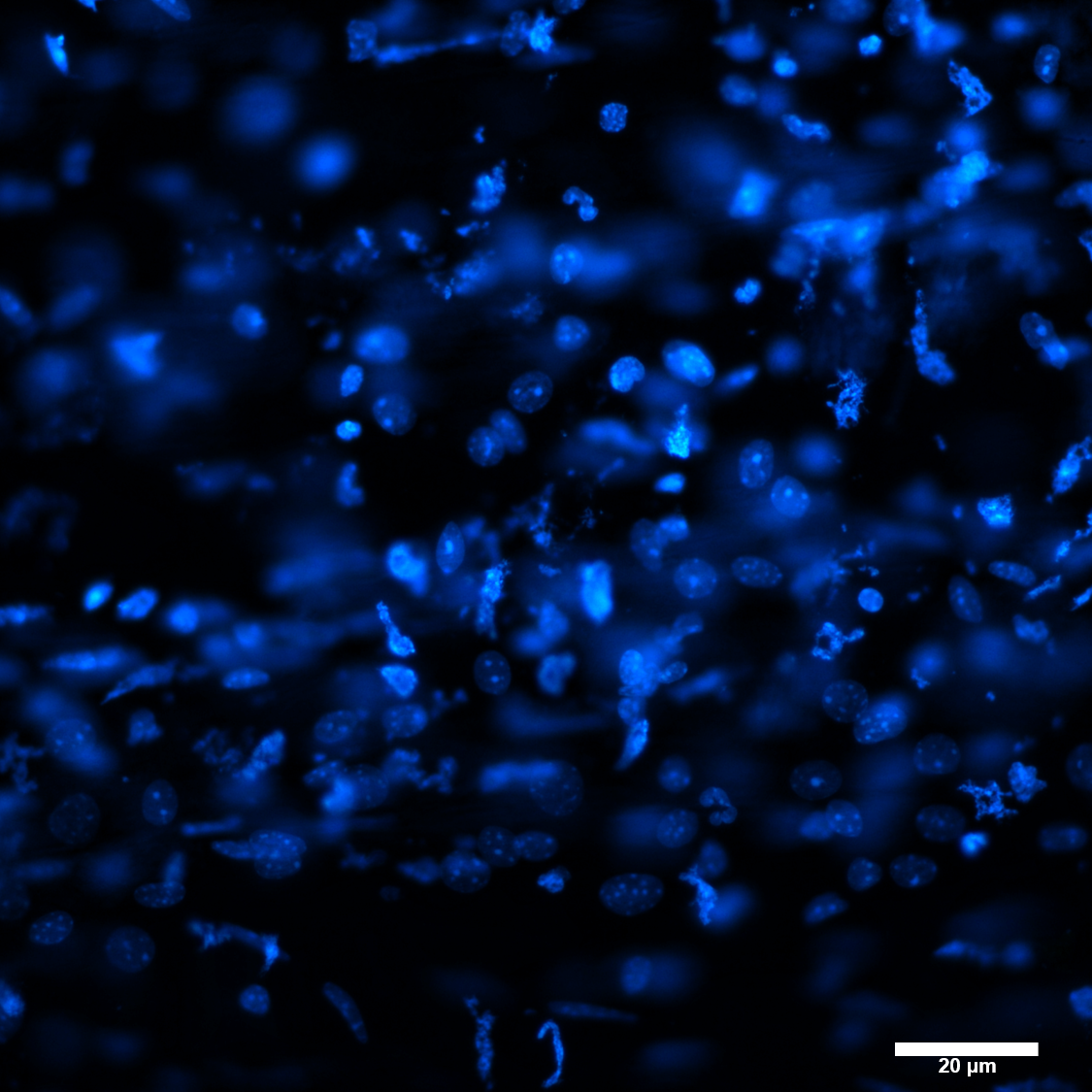

Supplement: Supplementary file 8 [file DataSheet5.zip › Fig 5 original data/Fig 5A/VCR-DAPI.tif]

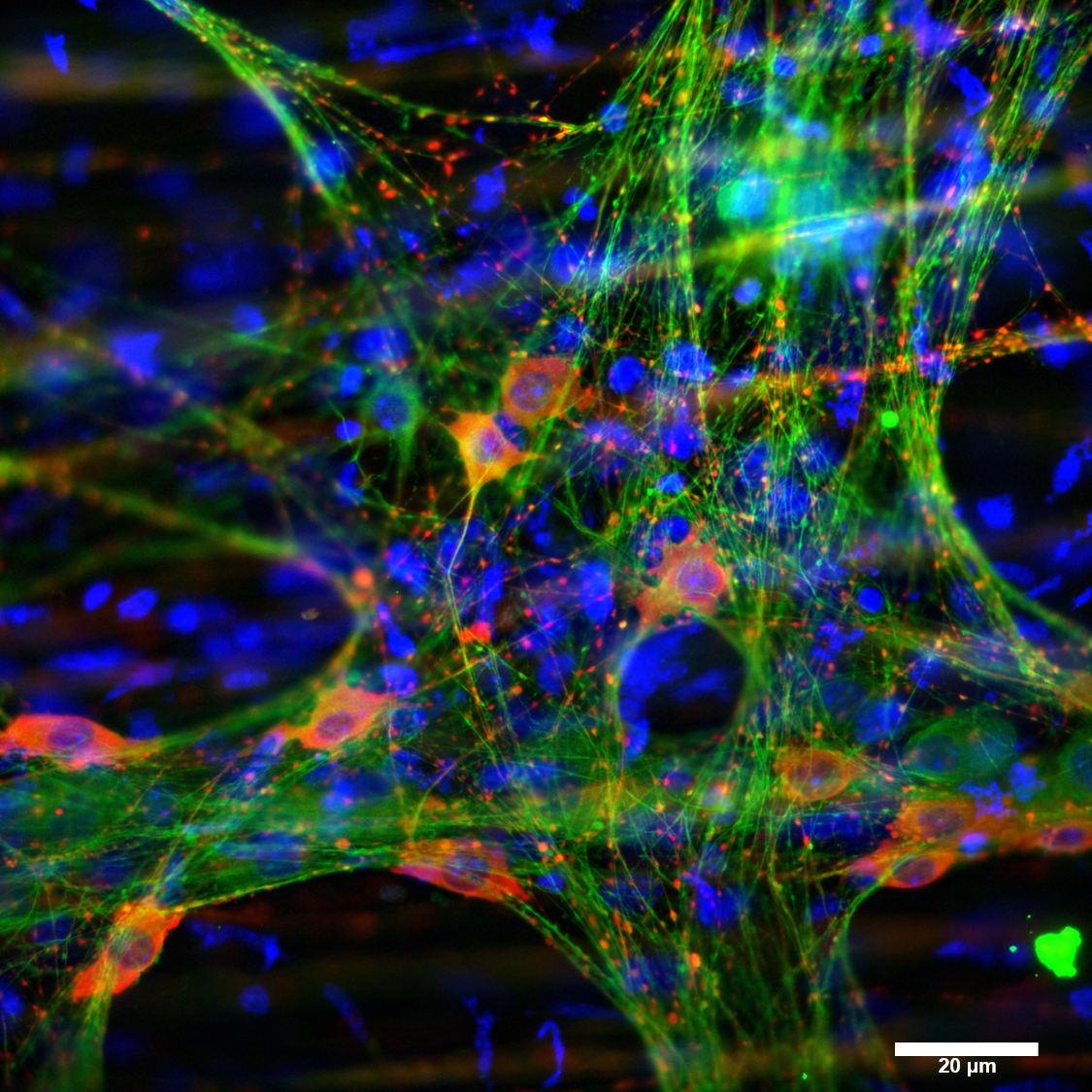

Supplement: Supplementary file 8 [file DataSheet5.zip › Fig 5 original data/Fig 5A/VCR-Merge.tif]

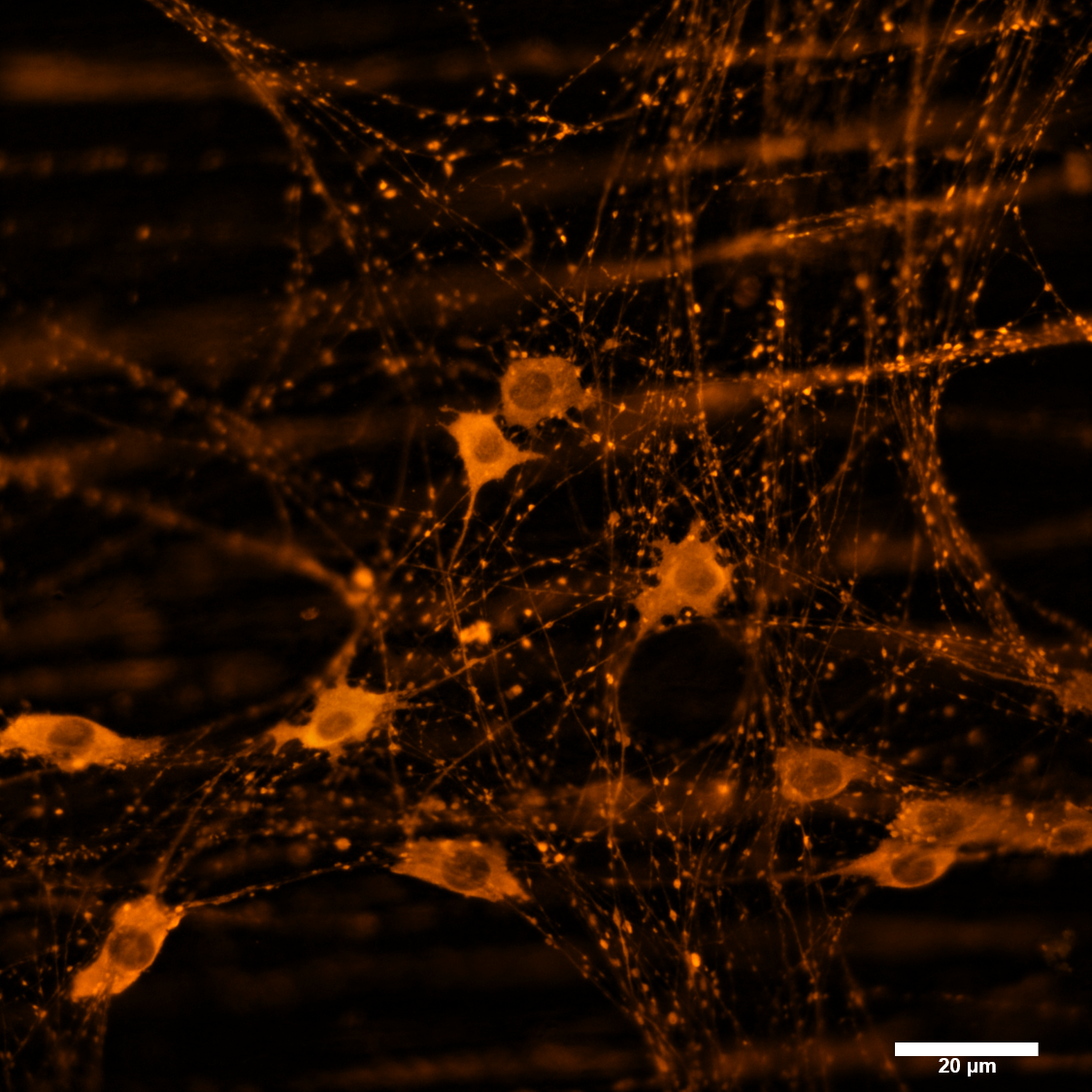

Supplement: Supplementary file 8 [file DataSheet5.zip › Fig 5 original data/Fig 5A/VCR-nNOS.tif]

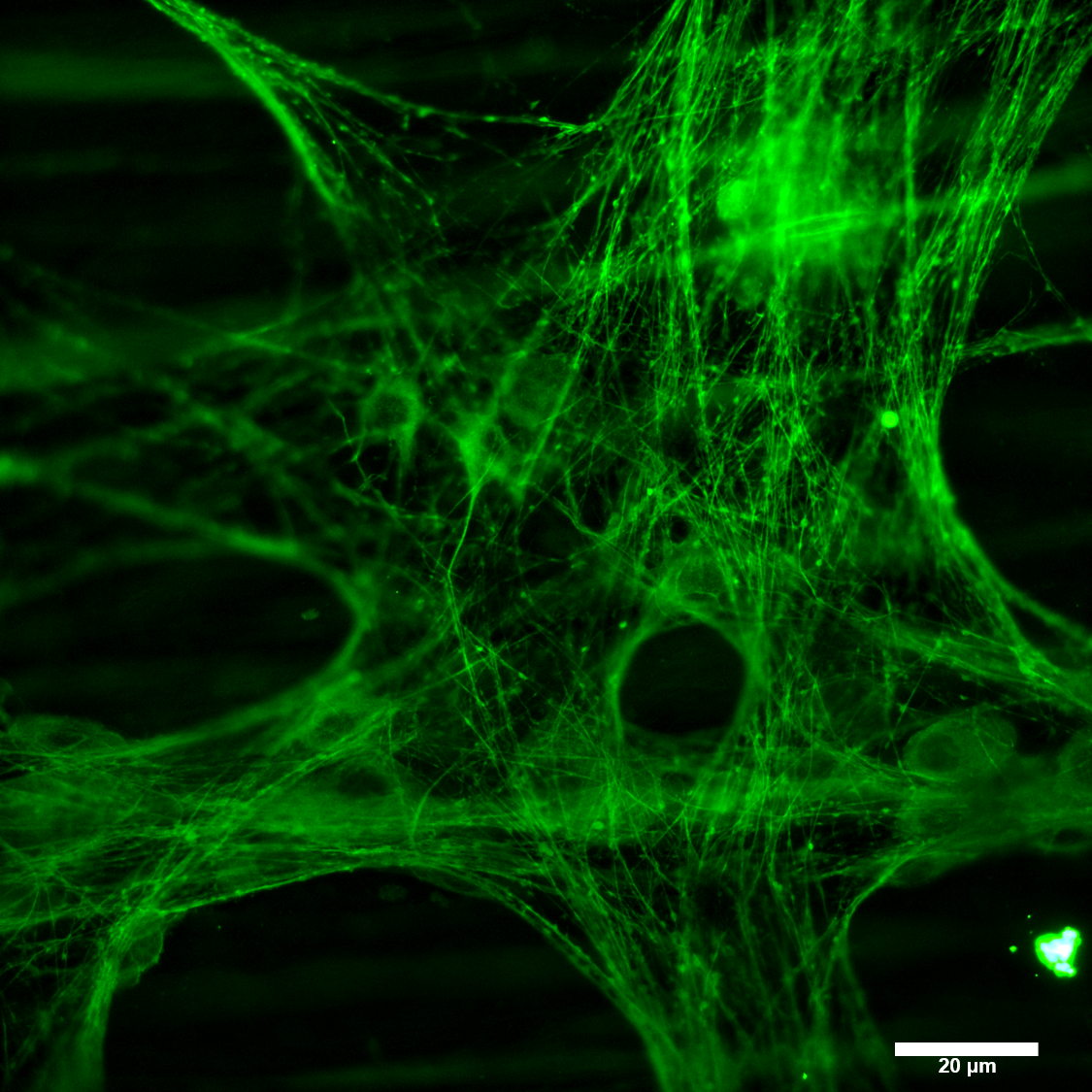

Supplement: Supplementary file 8 [file DataSheet5.zip › Fig 5 original data/Fig 5A/VCR-β III Tubulin.tif]

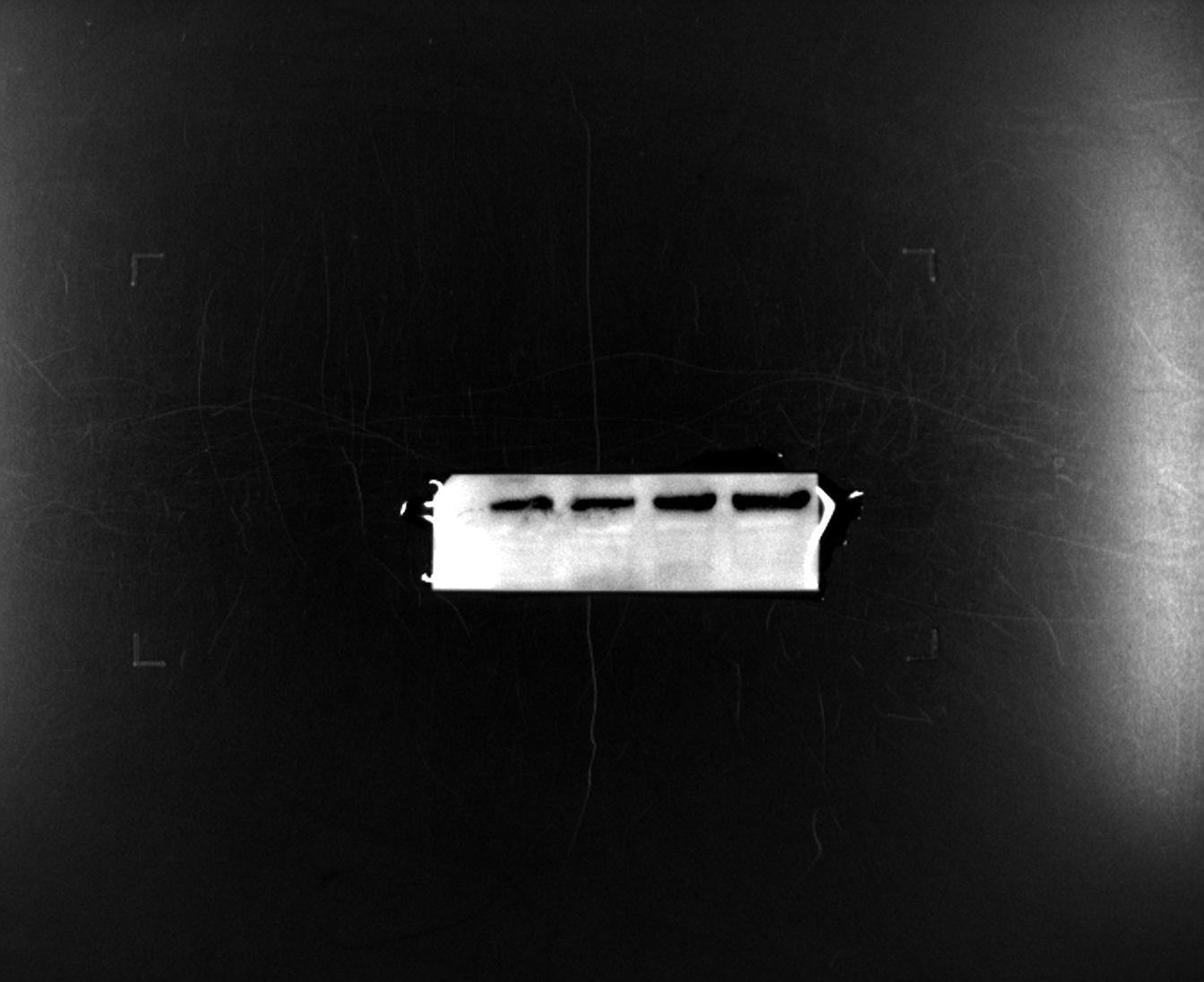

Supplement: Supplementary file 9 [file DataSheet7.zip › Fig 7 original data/Fig 7D/GAPDH.Tif]

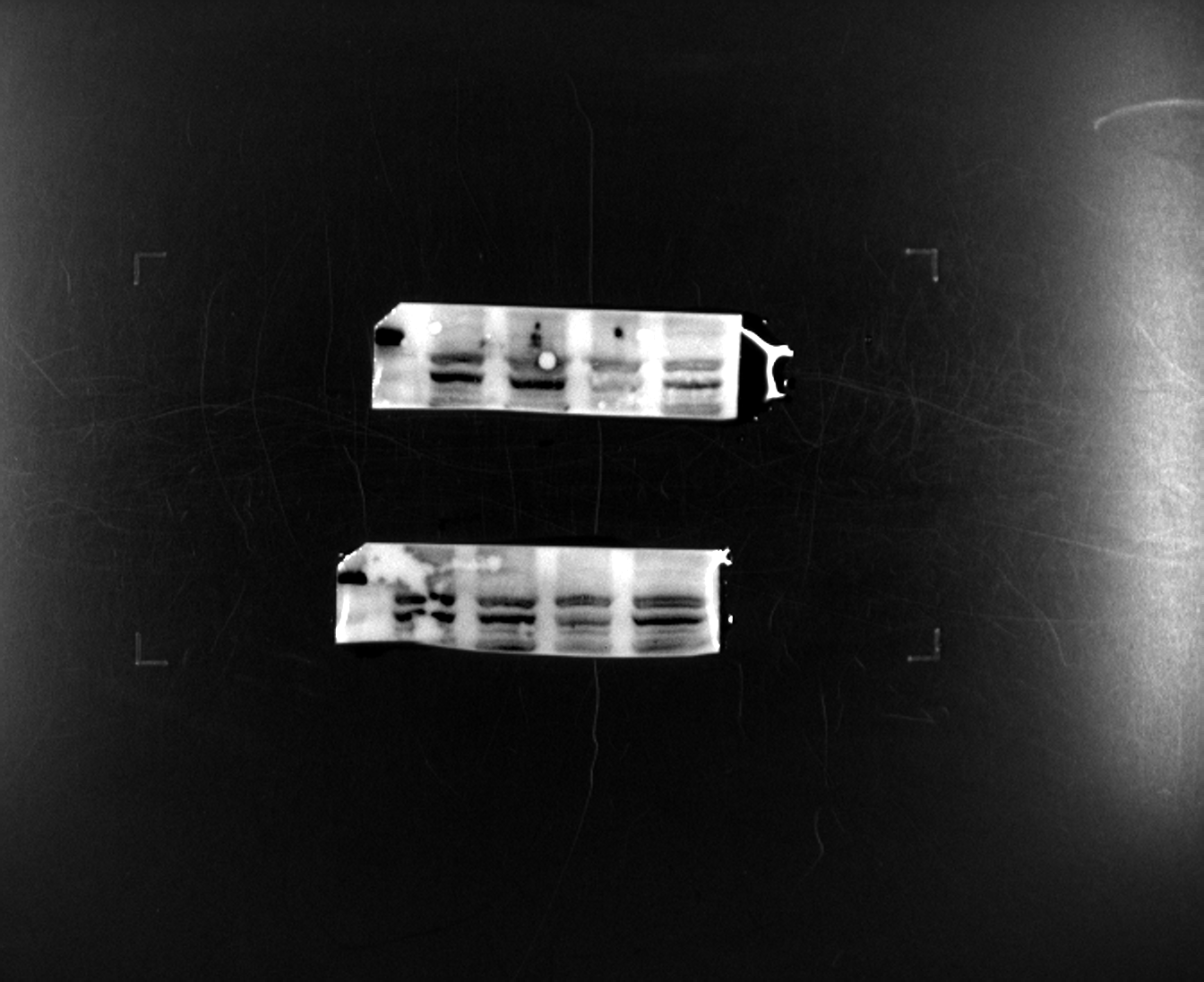

Supplement: Supplementary file 9 [file DataSheet7.zip › Fig 7 original data/Fig 7D/Nrf2.Tif]

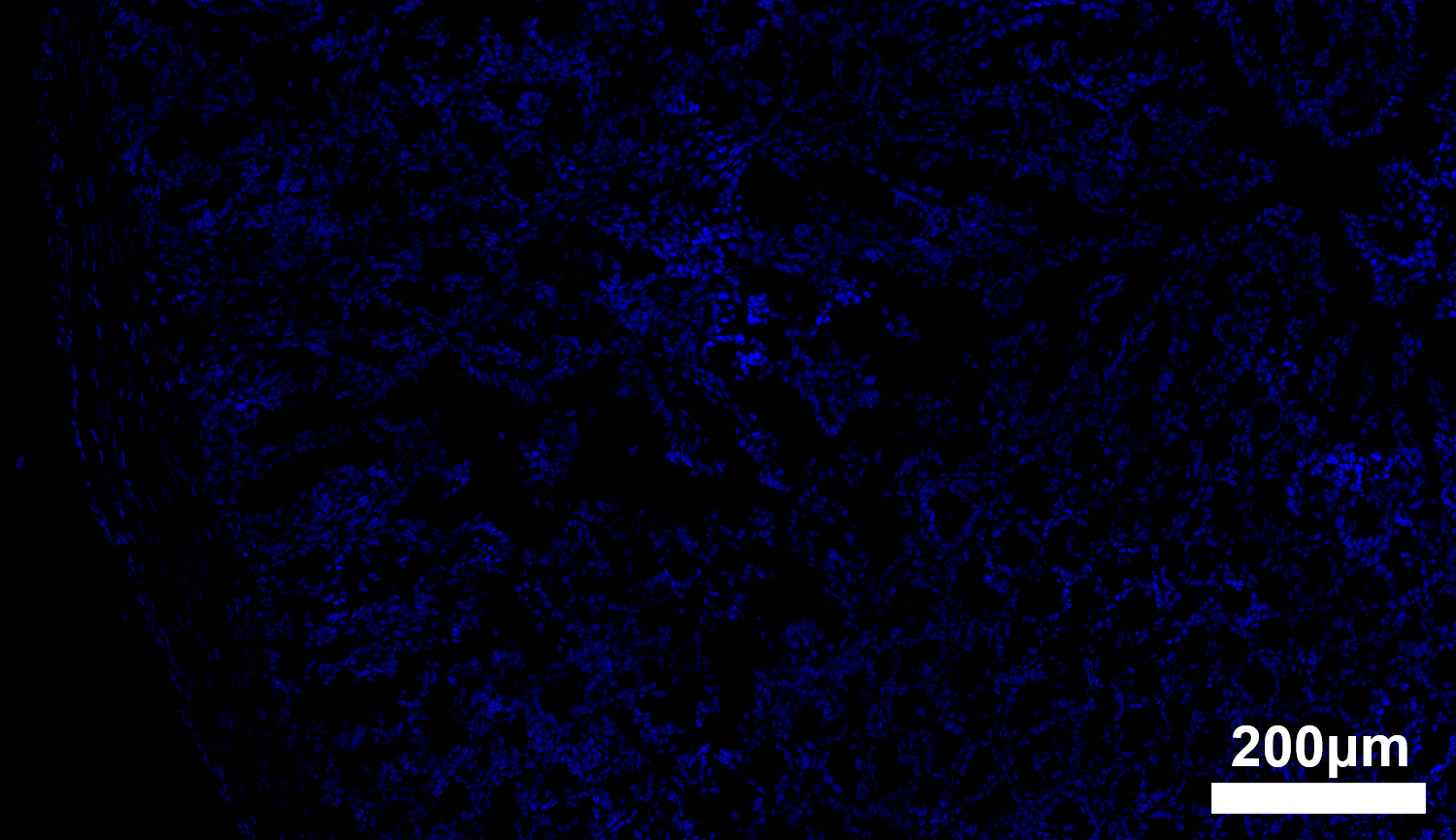

Supplement: Supplementary file 9 [file DataSheet7.zip › Fig 7 original data/Fig 7F/NS-DAPI.tif]

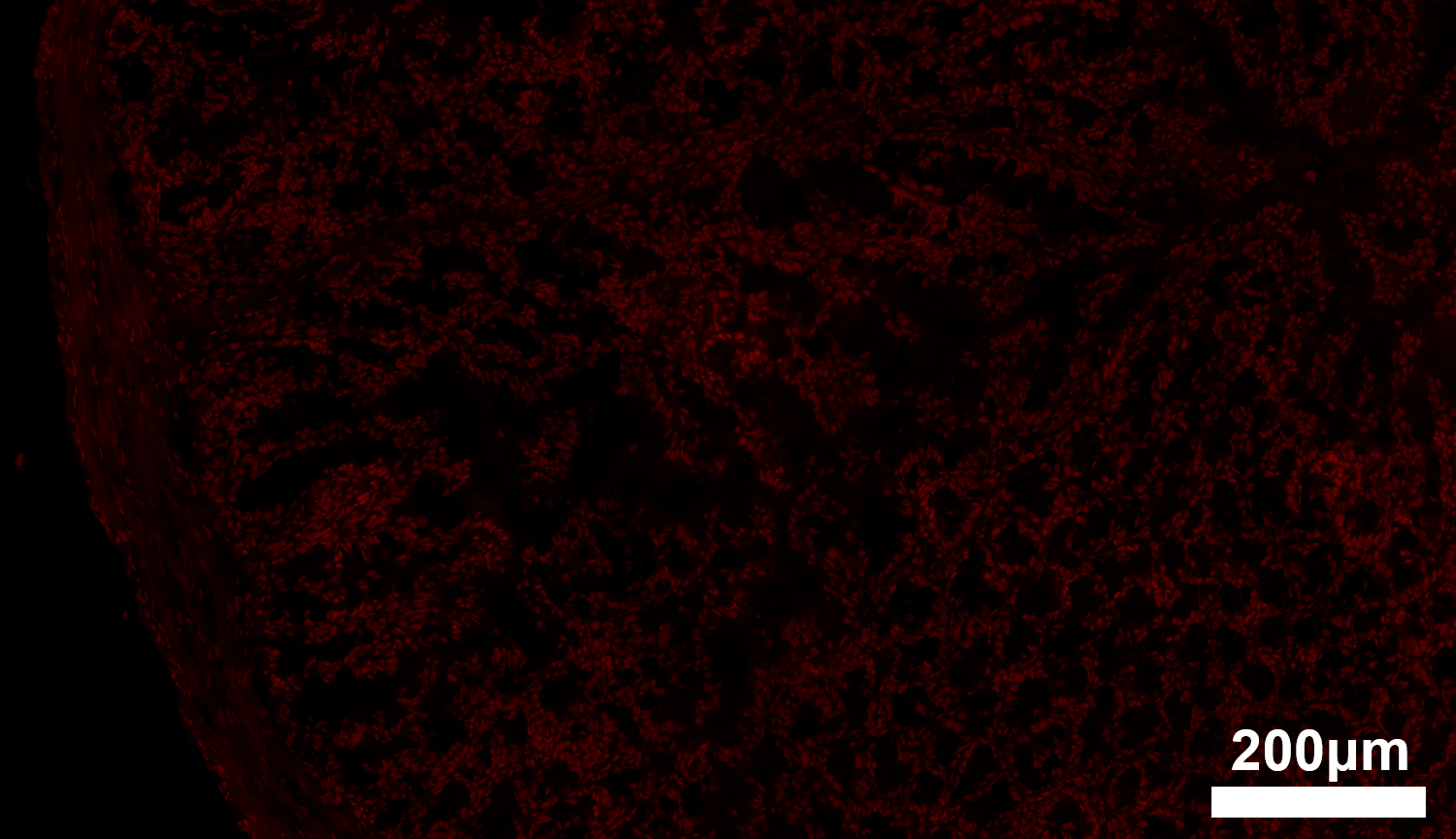

Supplement: Supplementary file 9 [file DataSheet7.zip › Fig 7 original data/Fig 7F/NS-DHE.tif]

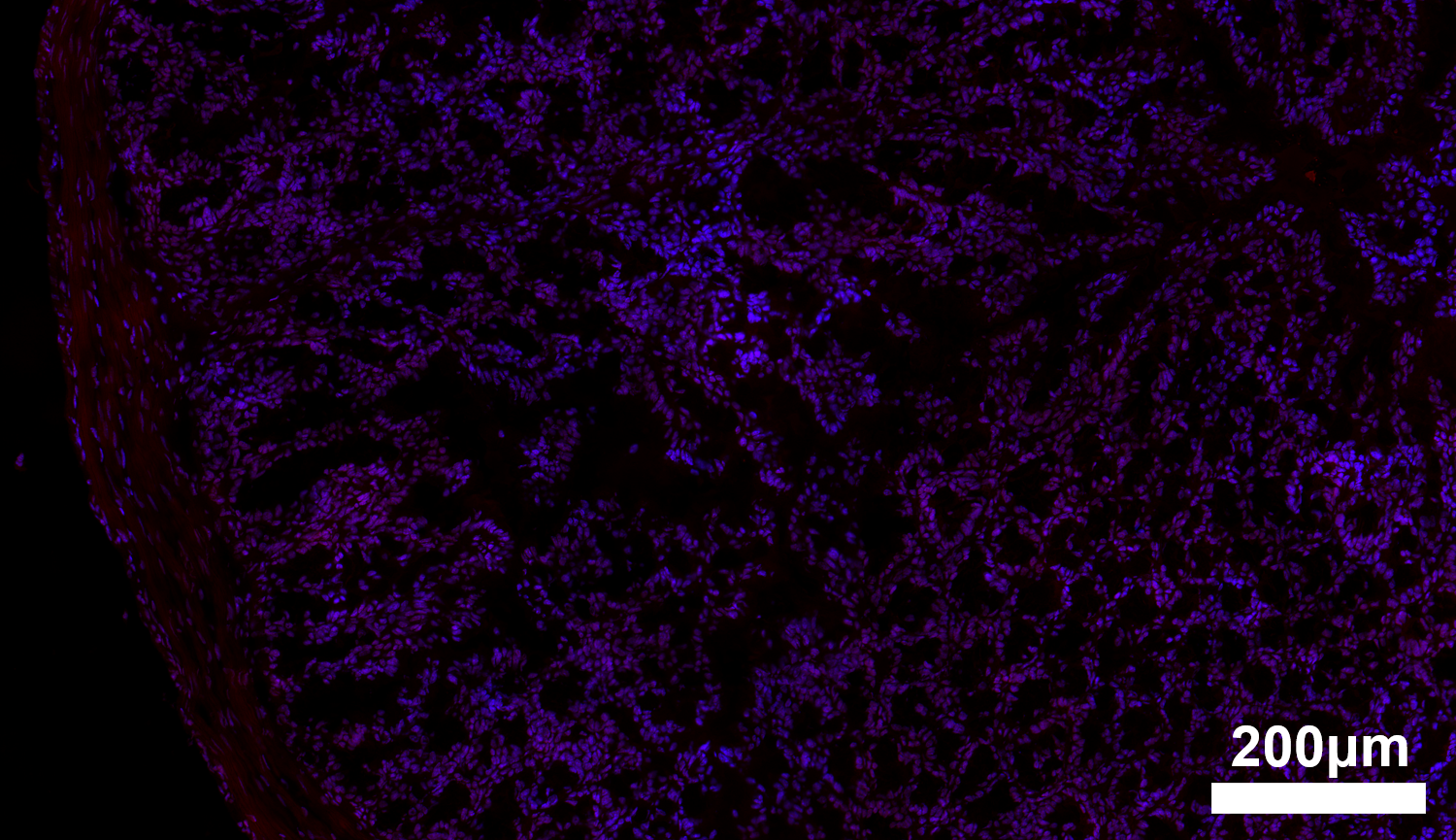

Supplement: Supplementary file 9 [file DataSheet7.zip › Fig 7 original data/Fig 7F/NS-Merge.tif]

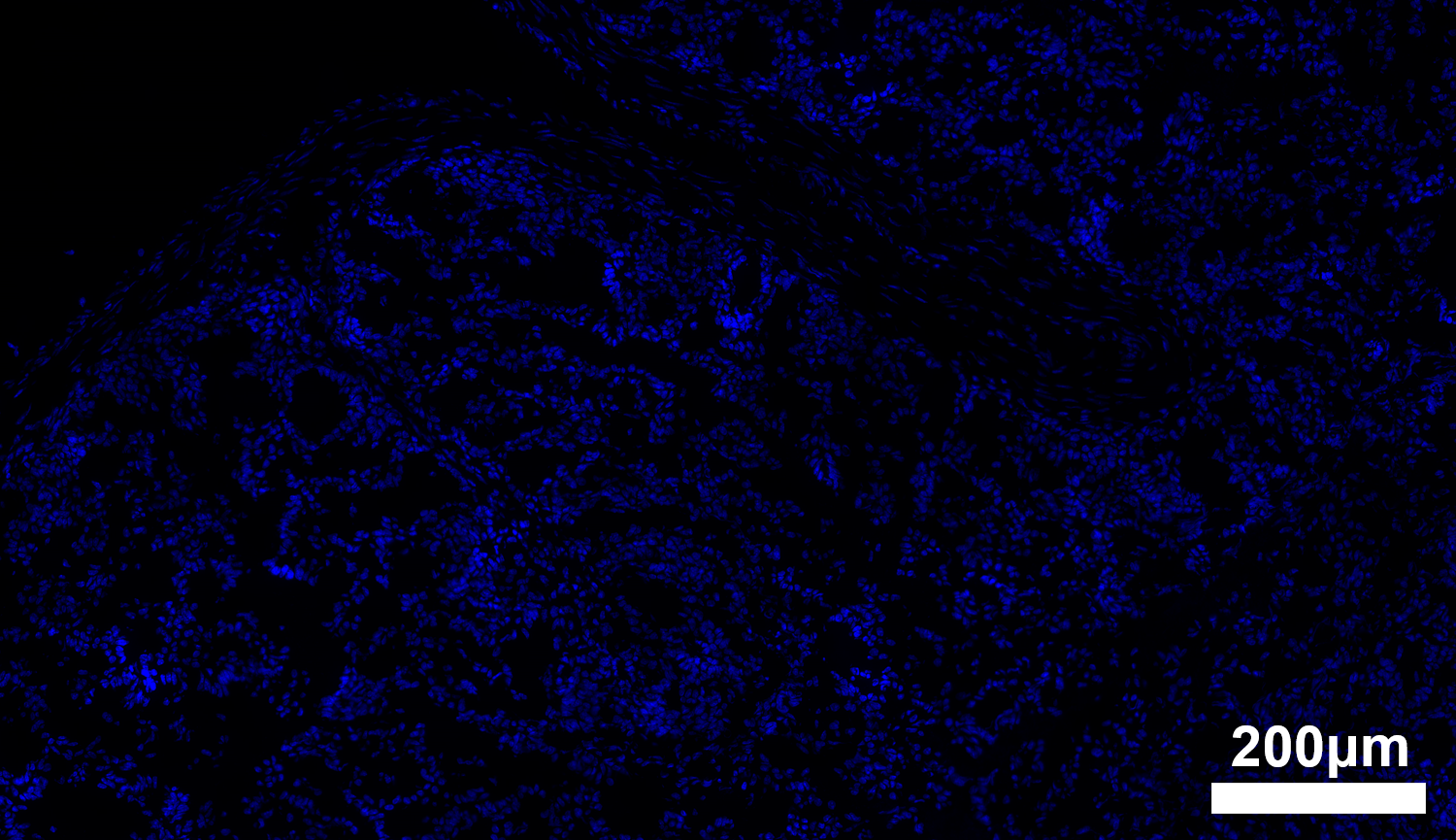

Supplement: Supplementary file 9 [file DataSheet7.zip › Fig 7 original data/Fig 7F/OT+VCR-DAPI.tif]

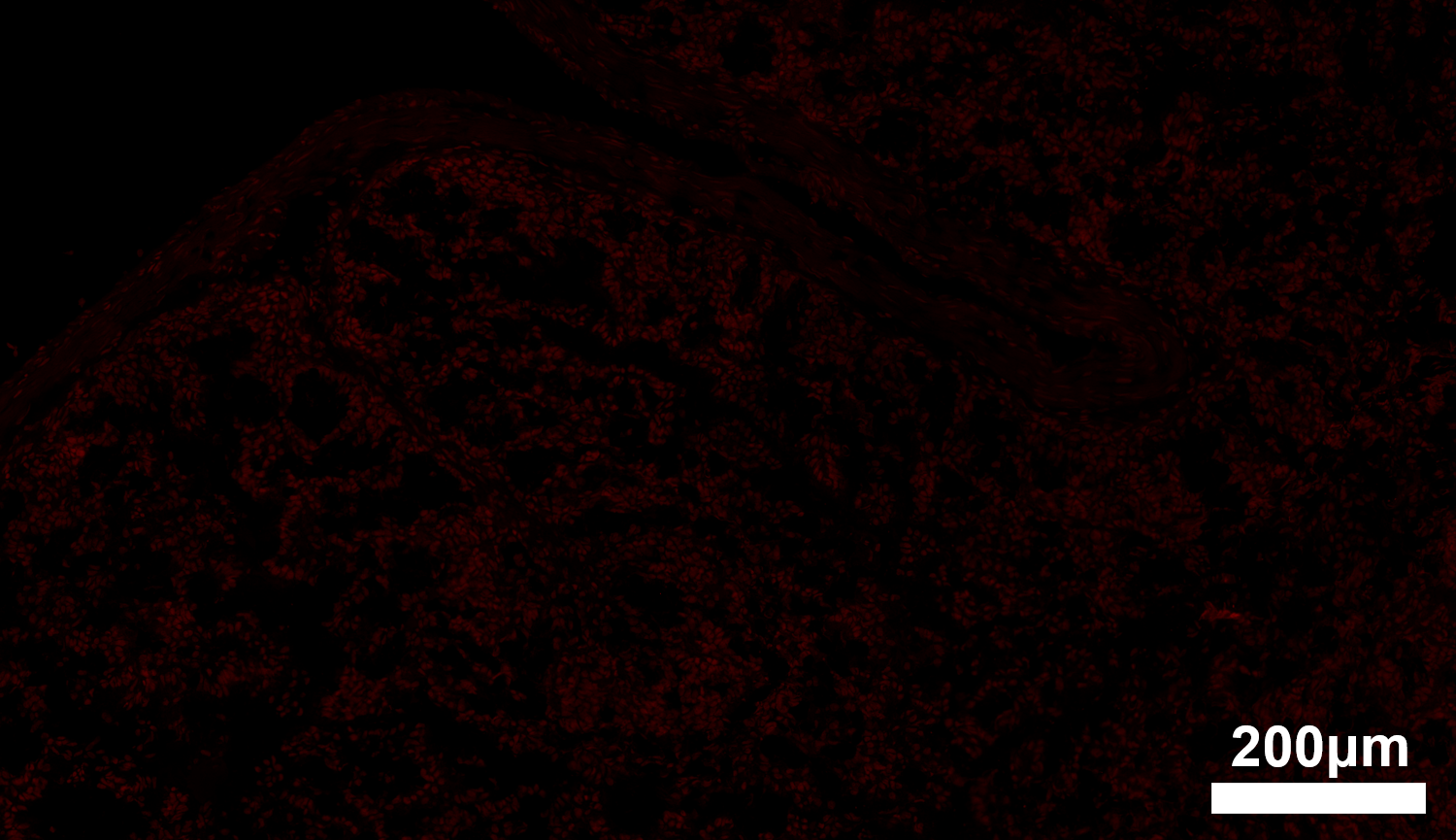

Supplement: Supplementary file 9 [file DataSheet7.zip › Fig 7 original data/Fig 7F/OT+VCR-DHE.tif]

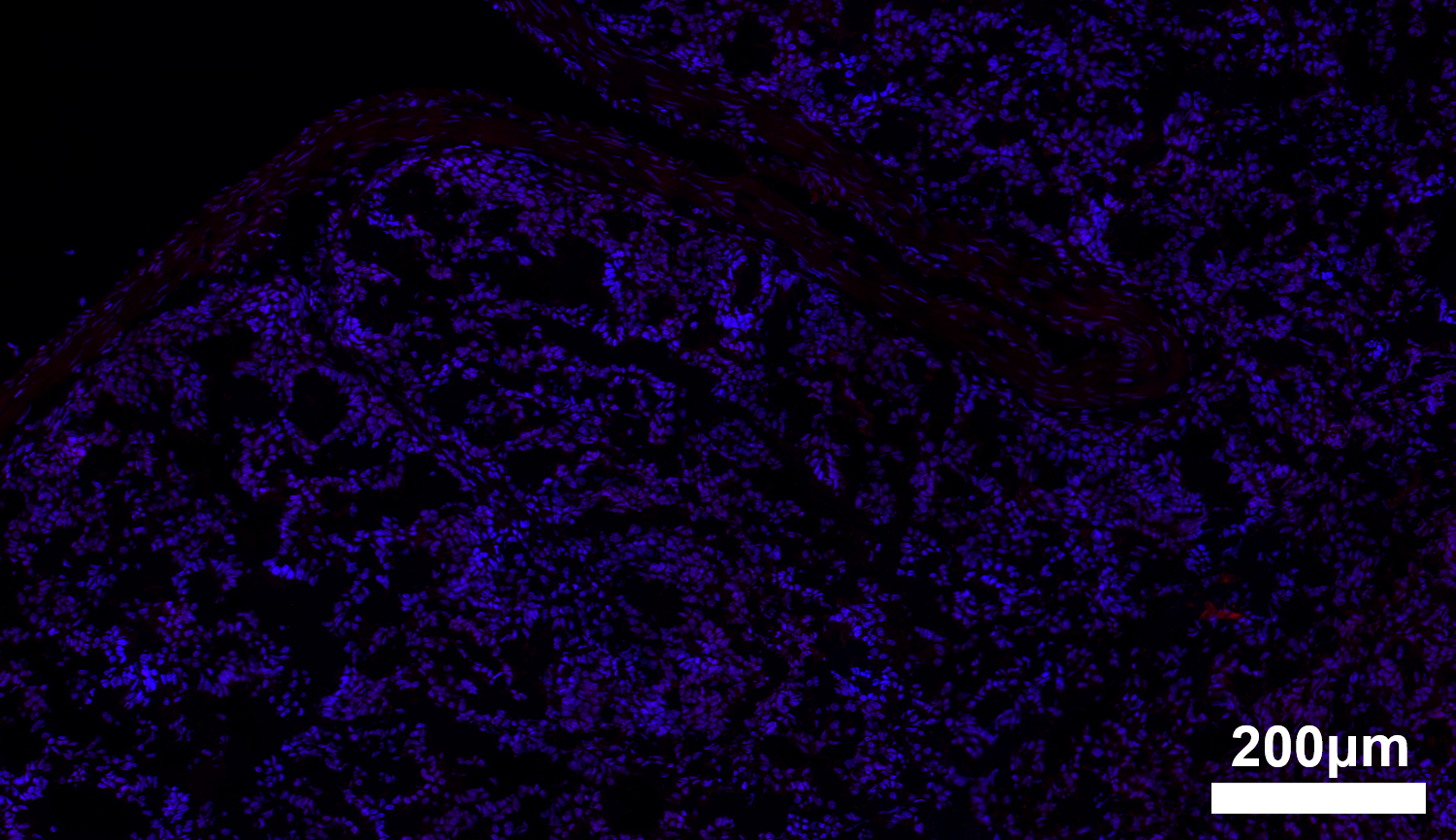

Supplement: Supplementary file 9 [file DataSheet7.zip › Fig 7 original data/Fig 7F/OT+VCR-Merge.tif]

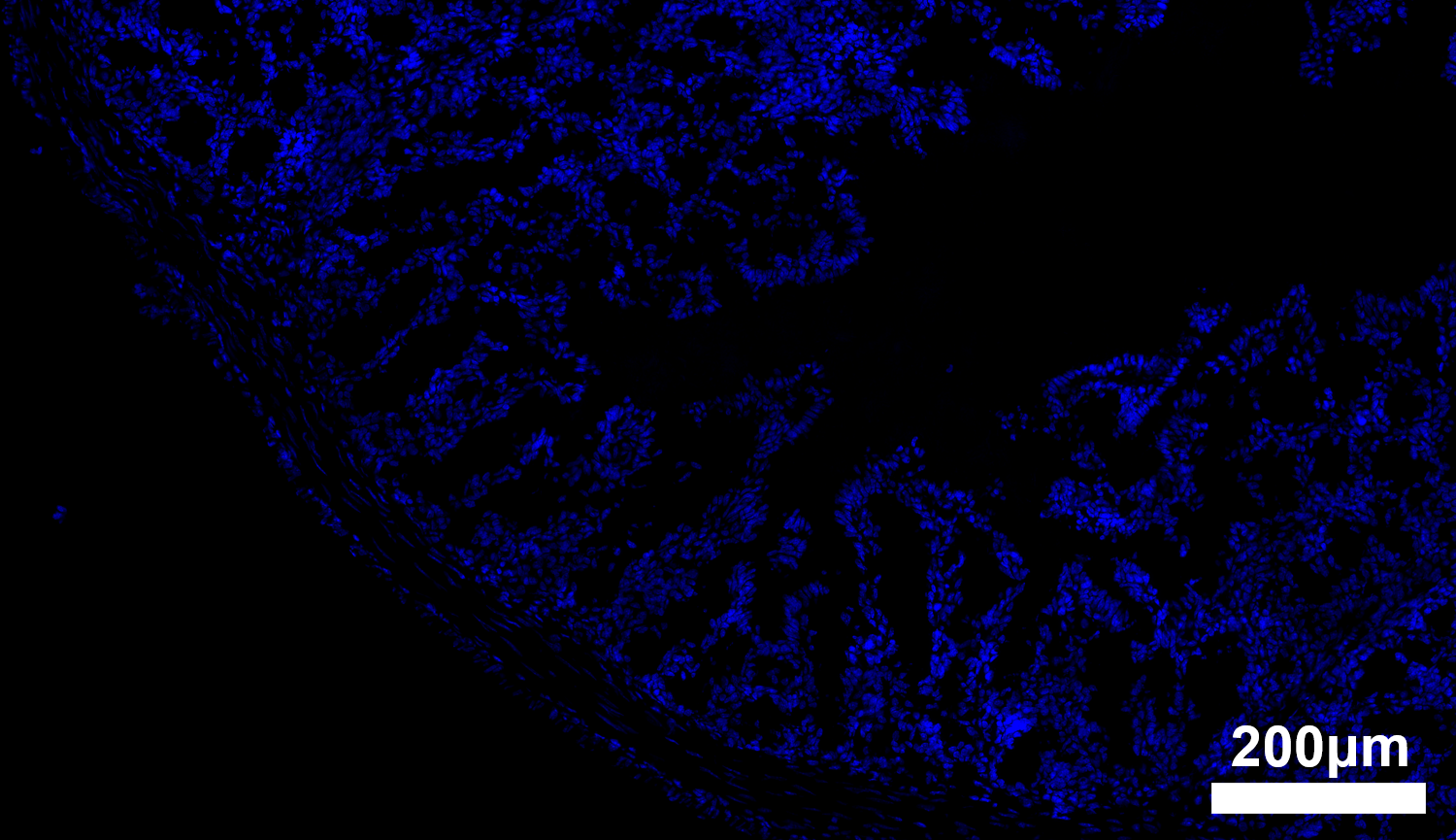

Supplement: Supplementary file 9 [file DataSheet7.zip › Fig 7 original data/Fig 7F/OT-DAPI.tif]

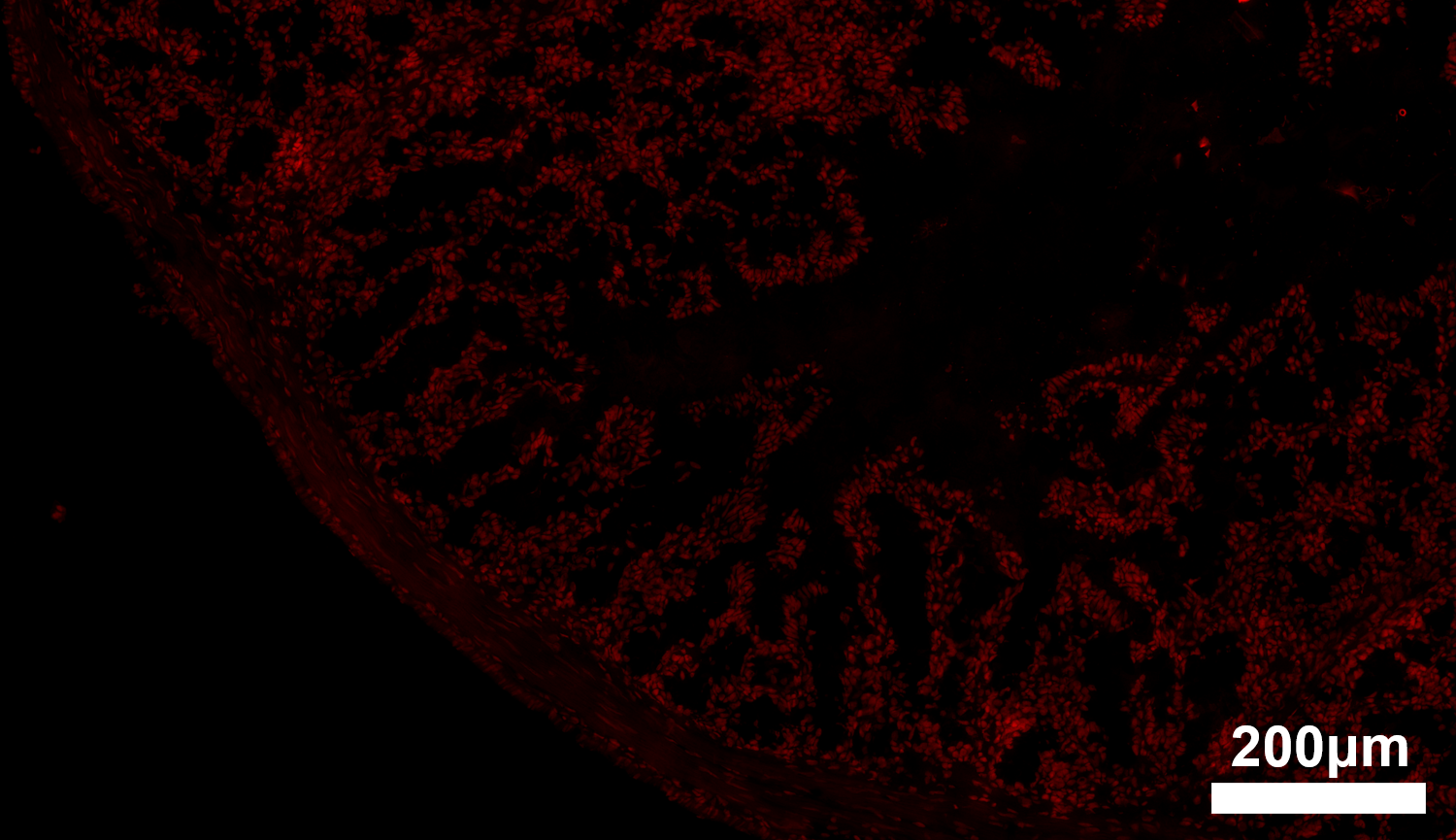

Supplement: Supplementary file 9 [file DataSheet7.zip › Fig 7 original data/Fig 7F/OT-DHE.tif]

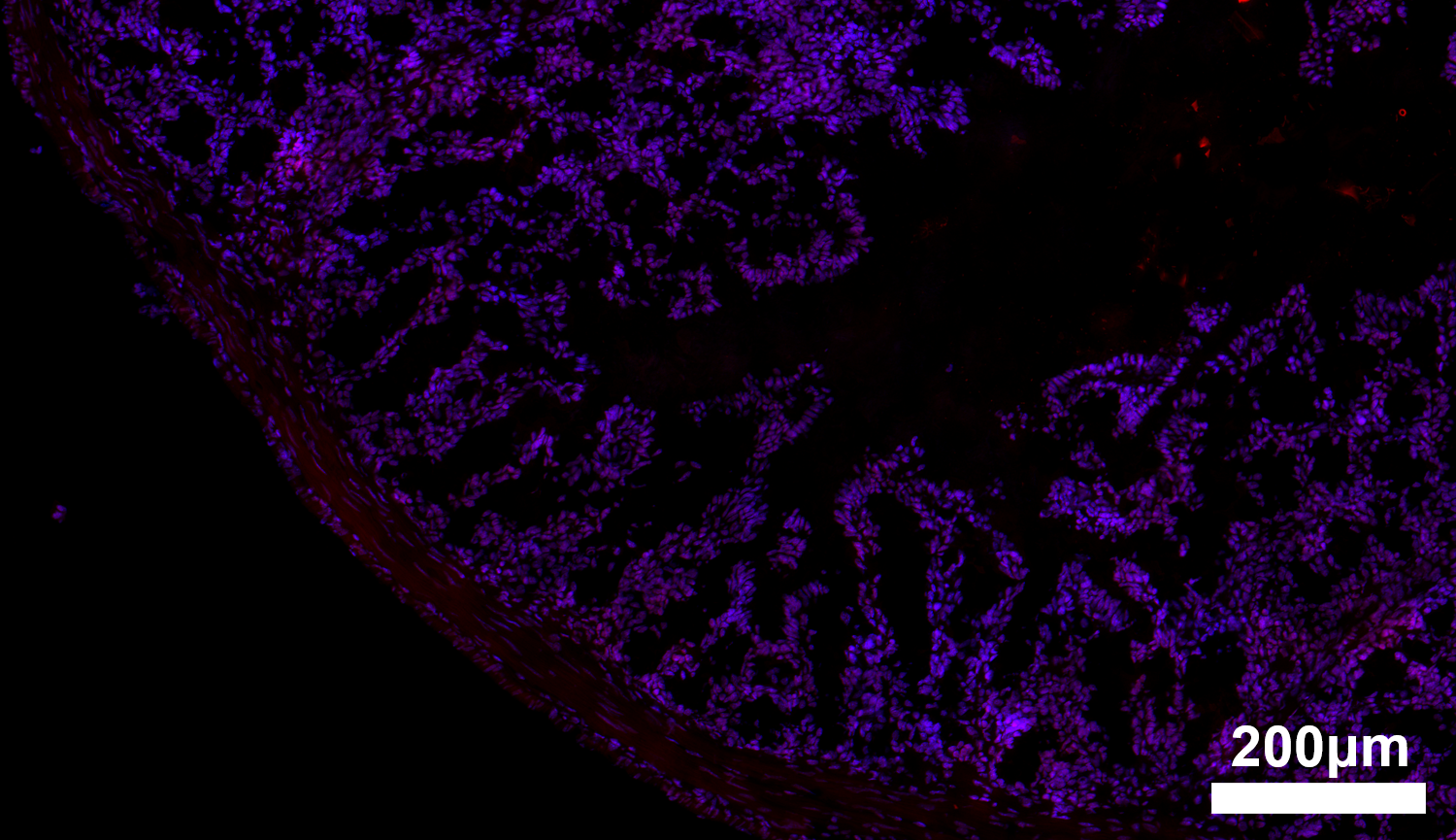

Supplement: Supplementary file 9 [file DataSheet7.zip › Fig 7 original data/Fig 7F/OT-Merge.tif]

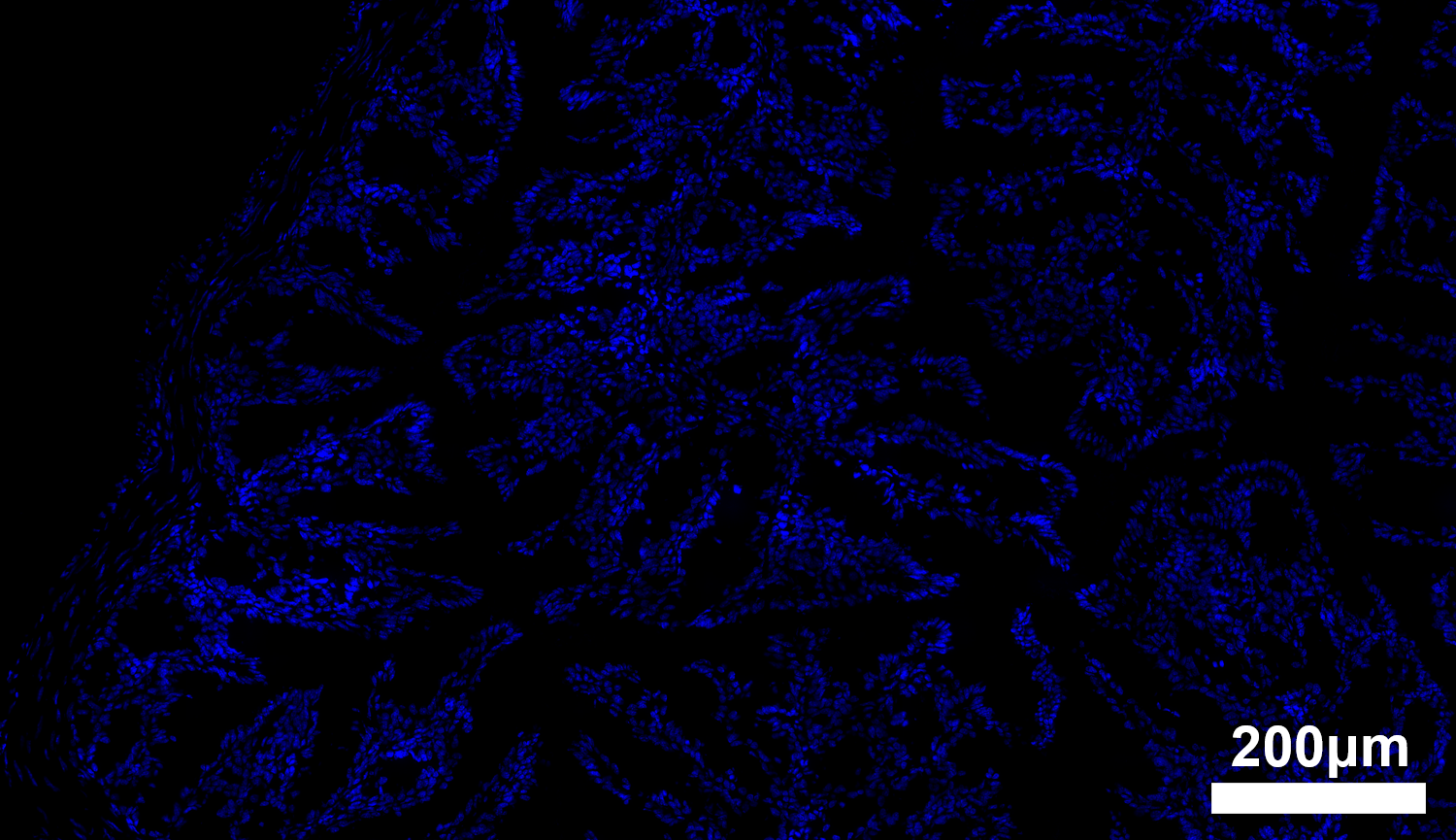

Supplement: Supplementary file 9 [file DataSheet7.zip › Fig 7 original data/Fig 7F/VCR-DAPI.tif]

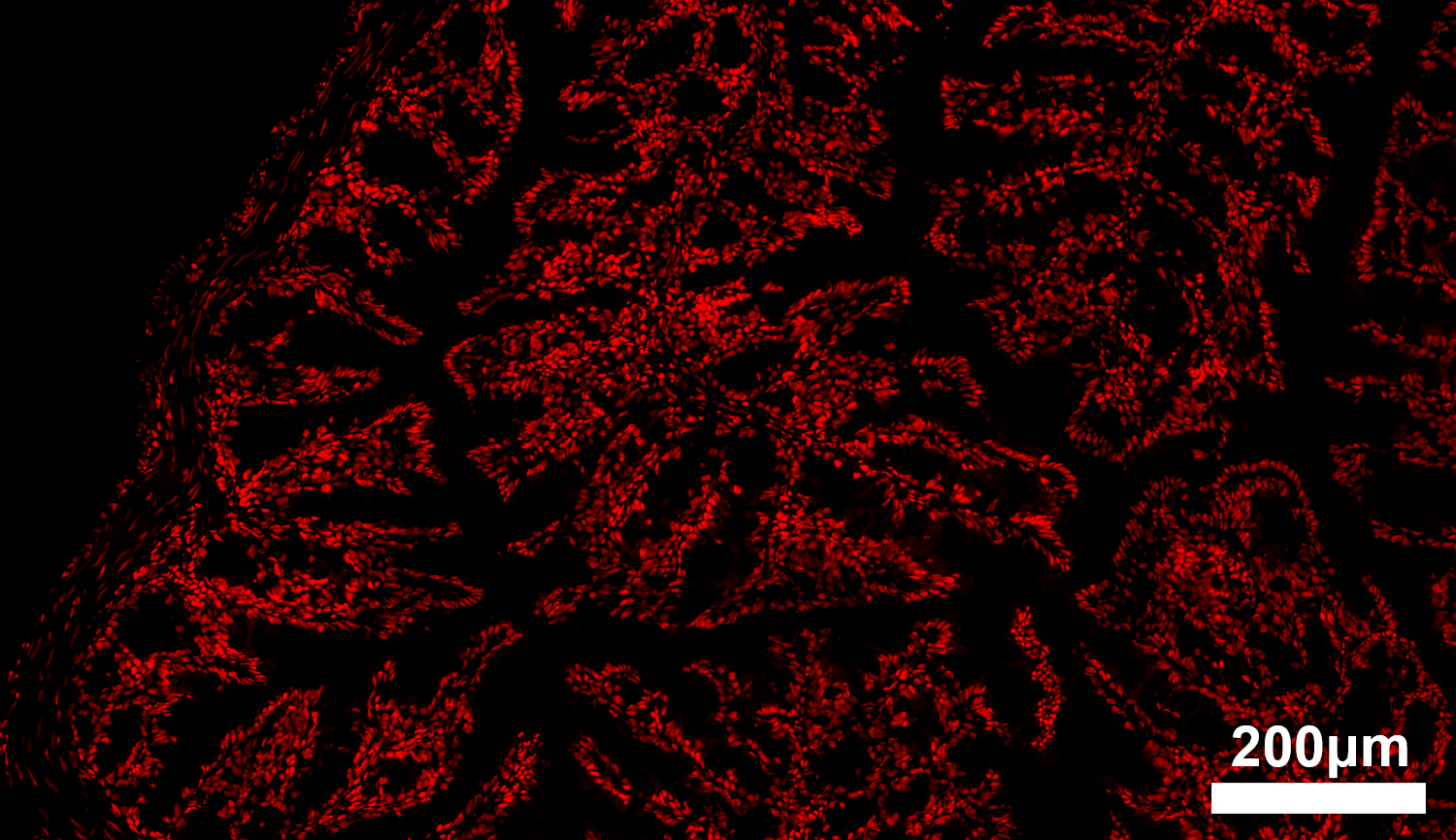

Supplement: Supplementary file 9 [file DataSheet7.zip › Fig 7 original data/Fig 7F/VCR-DHE.tif]

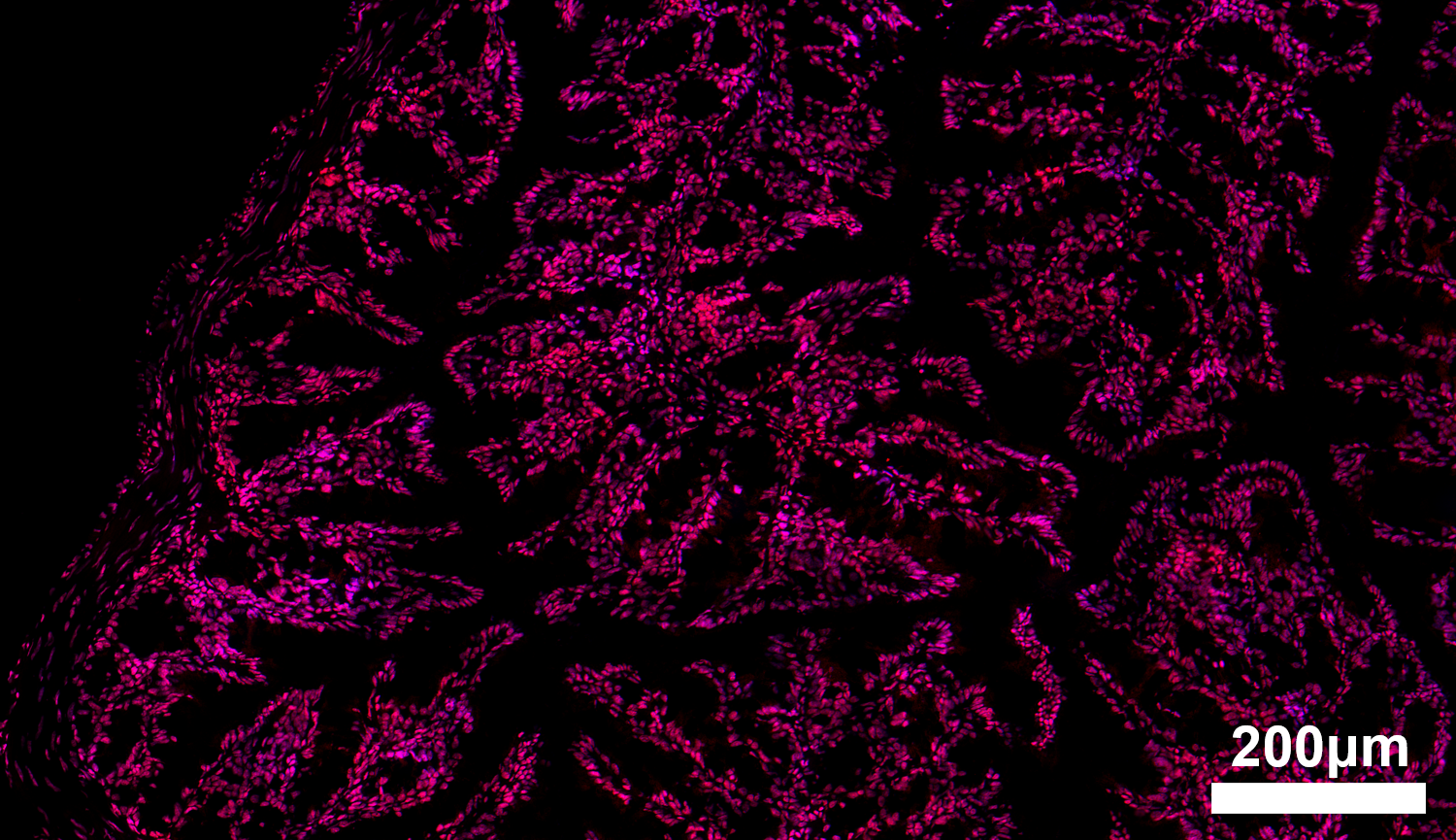

Supplement: Supplementary file 9 [file DataSheet7.zip › Fig 7 original data/Fig 7F/VCR-Merge.tif]
