# Supplementary material for: Protective effect of oxytocin on vincristine-induced gastrointestinal dysmotility in mice
Source: Front Pharmacol. 2024 Apr 9;15:1270612. doi: 10.3389/fphar.2024.1270612 (PMC11037254; doi:10.3389/fphar.2024.1270612)

Full scan of the original blots of cropped images shown in Figure 7D.

Lane1: marker

Lane2: NS

Lane3: OT

Lane4: VCR

Lane5: OT+VCR

Nrf2 (110kDa)


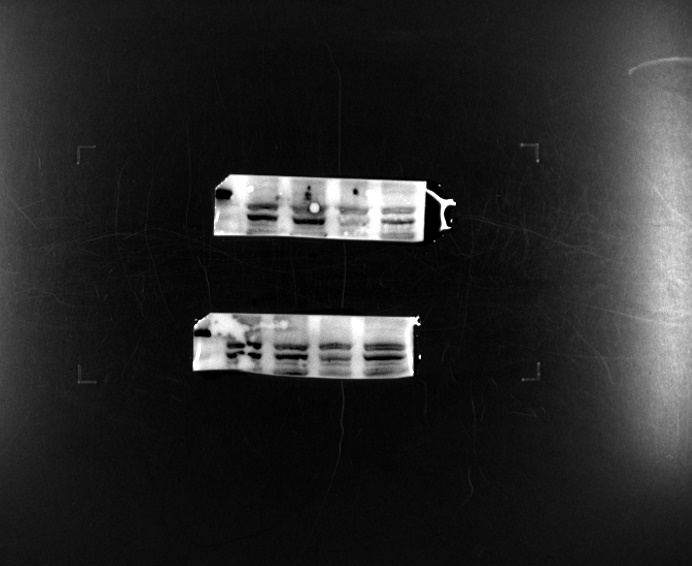


130kDa

100kDa

GAPDH (36kDa)


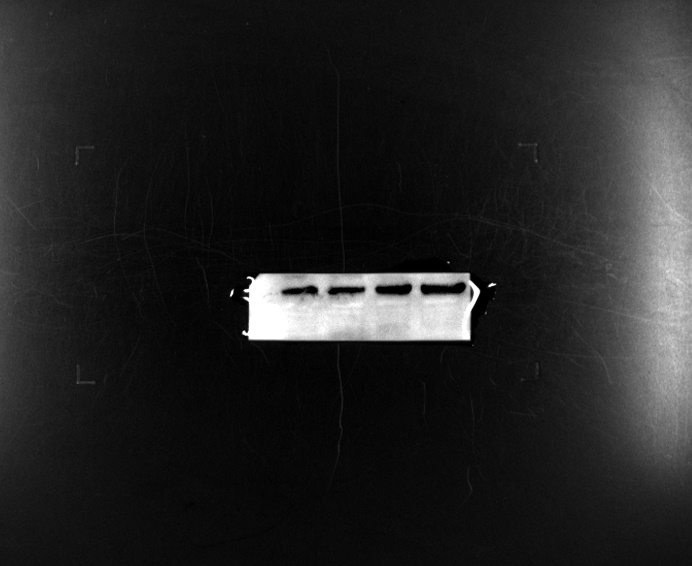

Supplement: Supplementary file 9 [file DataSheet7.zip › Fig 7 original data/Fig 7D/Fig 7D.docx]
