# Supplementary material for: Protective effect of oxytocin on vincristine-induced gastrointestinal dysmotility in mice
Source: Front Pharmacol. 2024 Apr 9;15:1270612. doi: 10.3389/fphar.2024.1270612 (PMC11037254; doi:10.3389/fphar.2024.1270612)
Supplement: Supplementary file 10 [file DataSheet1.docx]

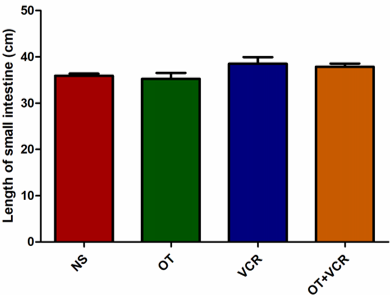


**Figure S1.** **The length of small intestine in the four groups indicates that there is no significant difference (n = 8).** The data are expressed as the means ± SEM, and one-way ANOVA combined with Newman–Keuls is used to compare the differences among multiple groups.


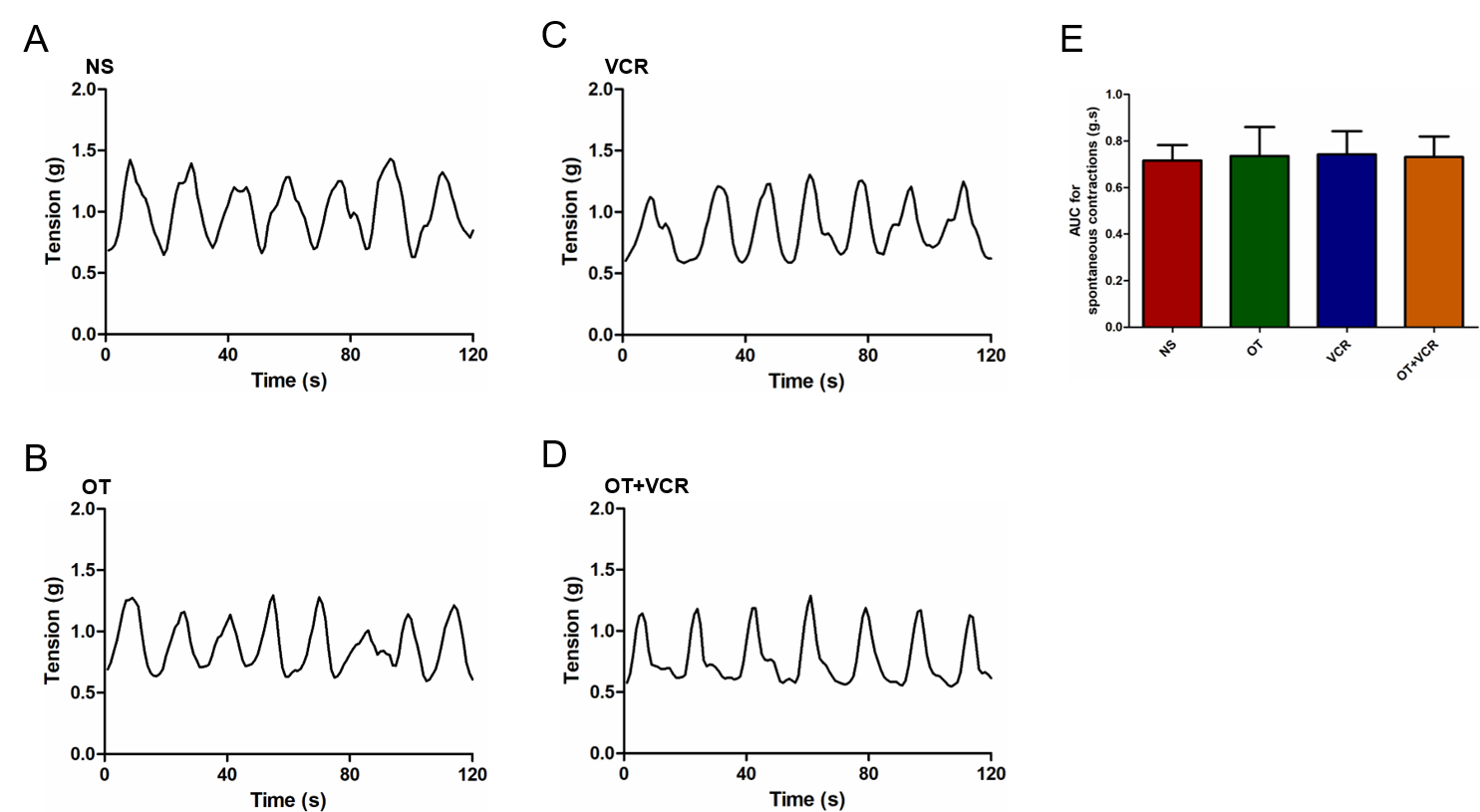


**Figure S2.** **There is no significant difference in the spontaneous contraction of isolated colonic segments among the four groups. (A-D)** Representative tension recordings shows the basal contractile activity in the four groups of mice: NS **(A)**, OT **(B)**, VCR **(C)**, and OT+VCR **(D)**. **E** The area under the curve (AUC) of spontaneous contraction within 1 min in the four groups indicates that there is no significant difference (n = 5–6). The data are expressed as the means ± SEM and one-way ANOVA combined with Newman–Keuls is used to compare the differences among multiple groups.
